# Spatiotemporal delivery of multifunctional nanozymes for neuroinflammation alleviation via autophagy modulation in spinal cord injury

**DOI:** 10.1016/j.mtbio.2025.102734

**Published:** 2025-12-23

**Authors:** Hongyi Jiang, Liting Jiang, Tian Xia, Jiachen Yu, Yitian Bu, Hanting Shen, Liang Zhu, Chihao Lin, Yumeng Wang, Yituo Chen, Rongjie Liu, Junfeng Shi, Jilong Wang, Junjie Deng, Haixiao Liu, Xiaoyun Pan

**Affiliations:** a Department of Orthopedics, The Second Affiliated Hospital and Yuying Children's Hospital of Wenzhou Medical University, Wenzhou, Zhejiang Province, China; b Key Laboratory of Orthopedics of Zhejiang Province, Wenzhou, Zhejiang Province, China; c The Second Clinical School of Medicine, Wenzhou Medical University, Wenzhou, Zhejiang Province, China; d Wenzhou Institute, University of Chinese Academy of Sciences, Wenzhou, Zhejiang, China; e The Affiliated XiangTan Central Hospital of Hunan University, School of Biomedical Sciences, Hunan University, China

**Keywords:** Nanozyme, Autophagy, Neuroinflammation, Spinal cord injury, Targeted delivery

## Abstract

While nanozymes effectively mitigate neuroinflammation by scavenging reactive oxygen species (ROS), they often fail to address the continuous ROS generation from dysfunctional cells within the spinal cord injury (SCI) microenvironment. Herein, we introduce a “root-cause” therapeutic strategy using a biomimetic nanoplatform (NMm-pPB-siRNA^TRAF6^ that integrates Prussian blue (PB) nanozymes with TRAF6-silencing siRNA to synergistically scavenge ROS and restore cellular homeostasis. Mechanistically, by restoring the impaired autophagic flux, this system effectively inhibits neuronal pyroptosis and reprograms pro-inflammatory M1 macrophages into the reparative M2 phenotype, thereby eradicating ROS at their source. Notably, inspired by the sequential infiltration of immune cells following SCI, we propose the concept of “spatiotemporal delivery”. Achieved through the camouflage of a hybrid neutrophil-macrophage membrane, this mechanism enables nanoparticles to continuously access at the lesion site throughout the critical window from Day 1 to Day 7 (temporal dimension) while precisely targeting the inflammatory microenvironment (spatial dimension). This biomimetic strategy significantly promoted functional recovery in SCI mice, providing a new paradigm for treating neuroinflammation-related diseases by simultaneously neutralizing oxidative stress and correcting upstream cellular dysfunction. The findings of this study not only deepen the understanding of disease-targeted delivery but also offer new insights for the construction and application of composite biomaterials.

## Graphical abstract

**Figure:**
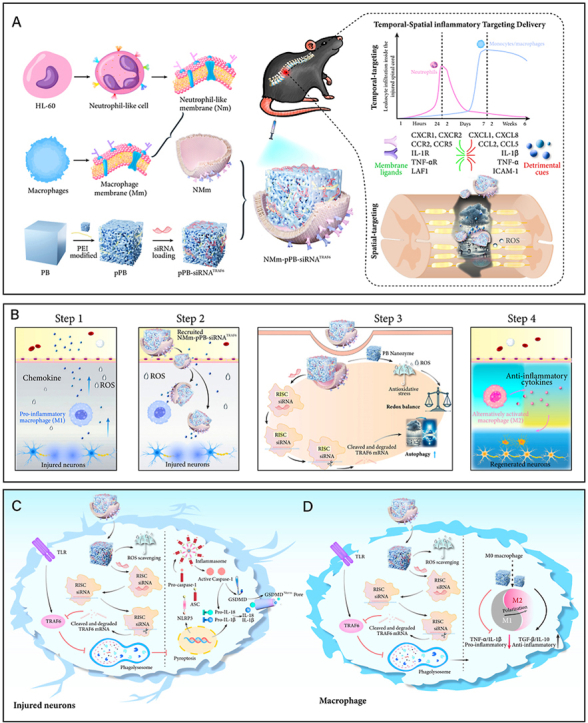

## 1 Introduction

Spinal cord injury (SCI) is a severe dysfunction of the central nervous system (CNS), leading to sensory or motor dysfunctions [1], affecting 250,000 to 500,000 individuals annually [2]. SCI treatment presents a significant challenge, imposing a heavy burden on patients, families and society [3]. From the perspective of the characteristics of the pathophysiological process, SCI can be categorized into primary or secondary injury [4,5]. Primary injury refers to nerve damage caused directly by external forces (e.g., vehicle accidents, falls, sports injuries, or violent trauma) to the spinal cord [4], which disrupts the blood-spinal cord barrier, eventually triggering inflammatory responses [6,7], ischemia/reperfusion injury [8], and oxidative stress [[9], [10], [11]]. Subsequently, the resultant ischemic/reperfusion injuries, inflammation, and reactive oxygen species (ROS) may further mediate secondary injury, commonly resulting in damage more severe than the initial trauma [12]. Particularly, ROS can accelerate neuronal pyroptosis and inflammation to exacerbate neural damage, highlighting the development of strategies to regulate ROS for managing secondary SCI [9].

Current therapeutic agents for regulating ROS show obvious anti-inflammatory mechanisms for treating secondary SCI [13]. However, these medications, necessitating prolonged usage, are accompanied by significant systemic adverse effects, such as inducing gastrointestinal bleeding and arthritis. Furthermore, antioxidants are another therapeutic options for exerting neuroprotective effects in secondary SCI [14]. For instance, superoxide dismutase (SOD) has been utilized in combination with nanocarriers to control ROS, which, however, fail in the dynamically complex neuroinhibitory microenvironment, attributable possibly to their limited operational stability, nonspecific targeting, and singular functionality [[15], [16], [17]]. Therefore, there is an urgent need to develop novel antioxidant alternatives to control the oxidative stress of secondary SCI. Significantly, nanozyme platform with enzyme-mimicking activities have been extensively investigated in treating oxidative stress-associated diseases, such as diabetic wounds [18,19], osteoarthritis [20,21], and SCI [9,10,22]. Among them, Prussian blue (PB) nanozymes stand out given their excellent efficiency of mimicking key anti-oxidative enzymes, such as peroxidase (POD), catalase (CAT), and SOD, facilitating the neutralization of a broad range of ROS involving superoxide radicals (O_2_•^-^), hydrogen peroxide (H_2_O_2_), and hydroxyl radicals (•OH) [23,24]. Consequently, they are instrumental in reducing oxidative stress and influencing inflammatory pathways. Currently, it has been recognized that nanozymes exhibit promise by neutralizing ROS in the extracellular matrix and cytoplasm, Nevertheless, such nanozymes possess transient effects, offering only symptomatic relief without addressing underlying cellular dysfunction issues [25]. Hence, the continued presence of dysfunctional cells [pyroptosis neurons and inflammatory (M1) macrophages] will still induce rapid recurrence of ROS, negating the initial therapeutic effects. Therefore, comprehensive strategies are required to control ROS levels and correct cellular dysfunction [25].

Autophagy is a critical cellular mechanism for clearing damaged organelles and proteins [26]. It is a fundamental cellular process involving lysosome-dependent degradation and recycling of cytoplasmic components (e.g., misfolded proteins and organelles) [27]. Considering its effect of keeping internal equilibrium by eliminating damaged cells, autophagy is a key player for maintaining cellular homeostasis and hence pivotal in secondary SCI [28,29]. Therefore, we propose a concept of integrating nanozymes with the coordination of autophagy to clear ROS from the source and reshape the microenvironment of secondary SCI. Recently, special attention has attached to the role of tumor necrosis factor receptor-associated factor 6 (TRAF6) in the response of the CNS to traumatic injuries [30], with the detection of significant increase in TRAF6 levels in traumatic brain injury and ischemic stroke models, which markedly inhibited autophagy and accelerated disease progression [[31], [32], [33], [34]]. Therefore, rejuvenating autophagy by suppressing TRAF6 expression may offer valuable reference for formulating a combined therapeutic approach to secondary SCI.

**Scheme. 1:**
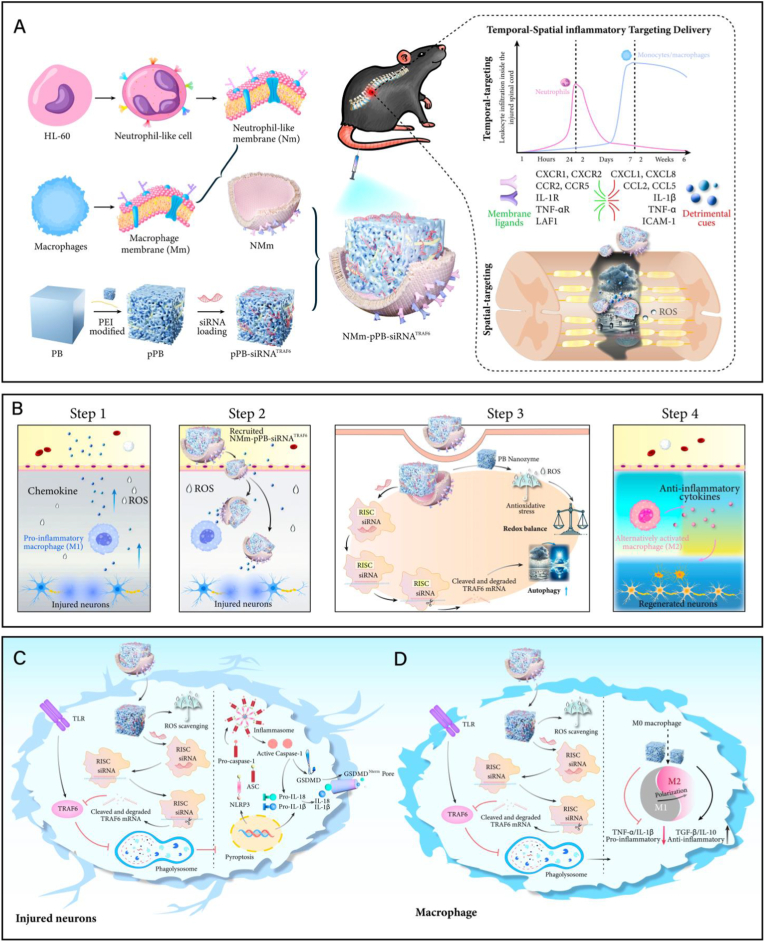
Spatiotemporal delivery of NMm-pPB-siRNA^TRAF6^ for ROS detoxification and autophagy regulation in SCI. (A) Schematic diagram of the preparation process for NMm-pPB-siRNATRAF6 and “spatiotemporal delivery” ability of NMm-pPB-siRNA^TRAF6^. PEI is used to modify PB, forming pPB. Then, siRNA^TRAF6^ is loaded into pPB to generate pPB-siRNA^TRAF6^. A hybrid membrane of Nm and Mm is further used to camouflage pPB-siRNA^TRAF6^, forming NMm-pPB-siRNA^TRAF6^. NMm-pPB-siRNA^TRAF6^ can both retain the sequential recruitment characteristics of neutrophils-macrophages after SCI, exhibiting “temporal” targeting ability, and also maintain the inflammatory chemotaxis capability of these cells in response to inflammatory factors, achieving “spatial” delivery. (B) After SCI, there may be persist pyroptotic neurons and inflammatory (M1) macrophages, promoting the release of inflammatory factors and continuous generation of ROS, presenting a “rainy day” (Step1). NMm-pPB-siRNA^TRAF6^ exerts “spatiotemporal delivery” ability, targeting the inflammatory region of SCI from both “temporal” and “spatial” dimensions (Step2). NMm-pPB-siRNA^TRAF6^ can synergistically alleviate oxidative stress and promote autophagy. NMm-pPB-siRNA^TRAF6^ can hold up an umbrella to shield against ROS “raindrops”, and the delivered siRNA^TRAF6^ can also form RISC to silence TRAF6, further promoting autophagy, clearing dysfunctional cells, and achieving a transition from “rainy” to “sunny” (Step3). NMm-pPB-siRNA^TRAF6^ achieves SCI regeneration landscape (“sunny day”) through synergistic ROS detoxification and autophagy. (C) NMm-pPB-siRNA^TRAF6^ can alleviate neuronal pyroptosis by promoting neuronal autophagy and regulate macrophage polarization through enhancing macrophage autophagy.

Herein, we developed a new combined therapeutic strategy for SCI, which had been proven to be highly effective in clearing ROS and promoting autophagy (Scheme 1). Our multifaceted approach includes: (1) the synthesis of engineered PB nanozymes that initiate efficient catalytic reactions, mimicking the activity of several anti-oxidative enzymes to detoxify ROS; (2) the pioneering design and application of siRNA^TRAF6^ sequences, loaded into PB modified with the cationic transfection agent polyethylenimine (PEI), named pPB-siRNA^TRAF6^. Upon release, it could form RNA-induced silencing complexes (RISC) in neurons to facilitate the alleviation of neuronal pyroptosis by effectively promoting neuronal autophagy (Scheme. 1C); and in macrophages, it could effectively advance macrophage autophagy to further regulate macrophage polarization (Scheme. 1D), ultimately reprogramming dysfunctional neurons and macrophages to clear ROS from the source (Scheme. 1B); (3) an innovatively composite membrane coating strategy in targeted delivery therapy for SCI. By employing a hybrid membrane (NMm) of neutrophil-like membrane (Nm) and macrophage membrane (Mm), this approach introduces a new concept in SCI treatment: “spatiotemporal delivery”. Specifically, on the “temporal” dimension, the use of single-cell RNA sequencing (scRNA-seq) has unveiled the characteristics of sequential recruitment of neutrophils and macrophages. The NMm coating grants nanoparticles the ability for “temporal” targeting. Simultaneously, on the “spatial” dimension, this NMm coating can preserve the inflammatory chemotaxis and homing responsiveness of neutrophils and macrophages, directing them towards the inflammatory region of SCI, thus achieving “spatial” targeting (Scheme. 1A). Lastly, we draw an evocative analogy, envisioning the spinal cord as the “sky”, with the occurrence of secondary SCI undoubtedly symbolizing a “rainy day” and ROS akin to “raindrops”. Our novel biomimetic nanoparticles (NMm-pPB-siRNA^TRAF6^) can both hold up an “umbrella” (control ROS), and also clear ROS from the source, transitioning the recovery of SCI from a “rainy” to a “sunny” state (promoting autophagy). Eventually, it may offer new therapeutic avenues for the treatment of SCI and other traumatic injuries of the CNS (Scheme 1).

## 2 Results and discussion

### 2.1 Synthesis and characterization of PB

**Fig. 1:**
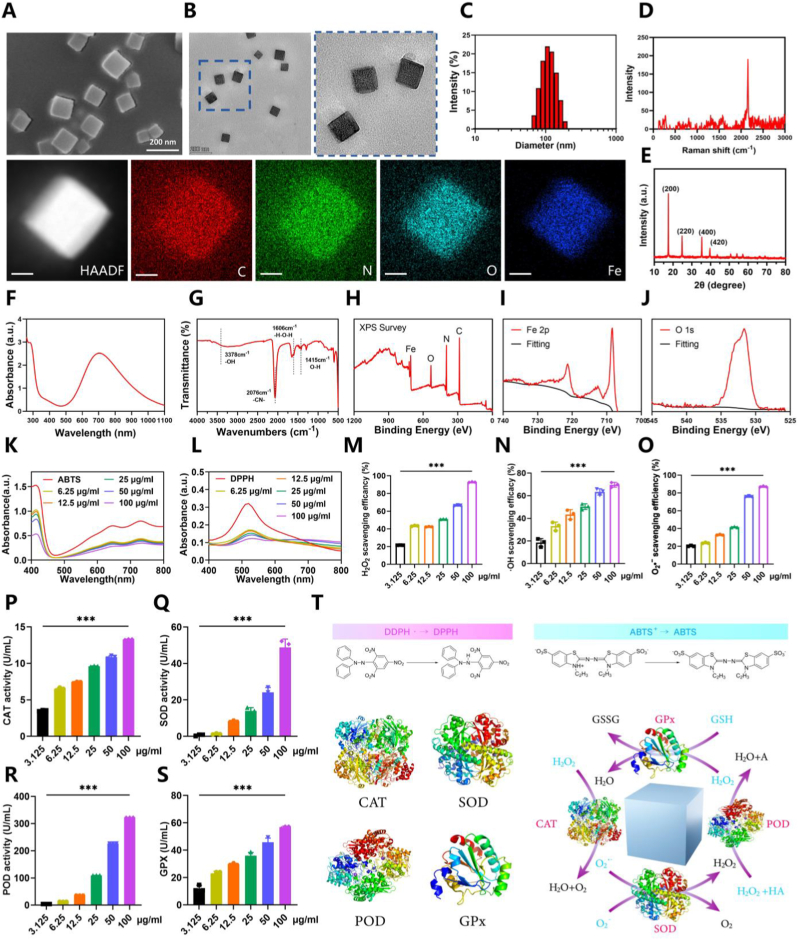
Preparation and characterization of PB. (A) SEM images of PB. (B) TEM images with corresponding elemental mappings of PB. (C) Diameters of PB. (D) Raman spectra of PB. (E) X-ray diffraction spectrum of PB. (F) Ultraviolet–visible–near-infrared (UV–vis–NIR) of PB. (G) FTIR spectra of PB. (H–J) XPS analysis of PB. (K) ABTS scavenging capability after incubation with PB at different concentrations (n = 3, mean ± SD). (L) DPPH scavenging capability after incubation with PB at different concentrations (n = 3, mean ± SD). (M–O) H_2_O_2_, •OH, O_2_•^-^ scavenging efficiency of PB at different concentrations (n = 3, mean ± SD). (P–S) CAT, SOD, POD, GPx-like activities of PB at different concentrations (n = 3, mean ± SD). (T) Schematic diagram of the scavenging processes for DPPH, ABTS, and the simulation of CAT, SOD, POD, GPx enzymes in ROS removal by PB. n represents the number of biologically independent samples. P values are shown in graphs with significance levels denoted as ∗P < 0.05, ∗∗P < 0.01, and ∗∗∗P < 0.001. Scale bars, 200 nm (A), and 200 nm (B).

This experiment synthesized PB nanozymes, coupled with corresponding characterization by various techniques, according to previously reported methods [35]. Based on the images of scanning electron microscopy (SEM) (Fig. 1A) and transmission electron microscopy (TEM) (Fig. 1B), the fabricated PB exhibited regular cubic structures, with good monodispersity and uniform siz of approximately 100–120 nm in diameter on average. Elemental mapping (Fig. 1B) indicated a uniform distribution of C, N, O, and Fe elements within the PB structure. The average hydrodynamic size of PB was around 110 nm (Fig. 1C), consistent with those revealed in SEM and TEM (Fig. 1A and B), with a zeta potential of about −32.9 mV (Fig. S1A). Raman spectroscopy of PB displayed a strong and sharp singlet at 2150 cm^−1^, indicative of Raman active modes of -CN- stretching in the cellular Raman silent region, without the observation of significant Raman signals of biomaterials (Fig. 1D). X-ray Diffraction (XRD) (Fig. 1E) showed good alignment of the characteristic peaks of the prepared PB with those of Fe_4_ [Fe(CN)_6_]_3_ (JCPDS #73–0687). UV–Vis–NIR absorption spectra of PB demonstrated broad absorption in the near-infrared region, peaking at 700 nm, which was attributable to the intermetallic charge transfer band from Fe(II) to Fe(III) within the PB structure (Fig. 1F). Furthermore, FTIR spectra revealed a principal peak at 2076 cm^−1^, characteristic of -C ≡ N- stretching, owing to Fe(II)-CN-Fe(III) bonds, while a broad peak near 3378 cm^−1^ related to hydroxyl (O-H) stretching, and a peak at 1606 cm^−1^ to H-O-H bending (Fig. 1G). According to X-ray Photoelectron Spectroscopy (XPS) (Fig. 1H–J, and Fig. S1, B and C), the full XPS spectrum of PB (Fig. 1H) indicated the presence of C, N, O, and Fe elements. The binding energies for N1s, C1s, and O1s were 397.6 eV, 296.7 eV, and 531.7 eV, respectively. The XPS spectrum of Fe2p (Fig. 1I) revealed peaks at 712.66 eV for Fe2p^3/2^ and 721.3 eV for Fe2p^1/2^, corresponding to Fe(III) in Fe_4_ [Fe(CN)_6_]_3_, with a peak at 708.4 eV representing Fe2p^3/2^ in [Fe(CN)_6_]^4-^. In addition, for subsequent evaluation of the stability of PB, the hydrodynamic diameter and polydispersity index (PDI) were assessed over 7 days of storage in phosphate buffered saline (PBS) and Dulbecco's modified Eagle medium (DMEM), showing no significant changes (Fig. S1, D and E). This result suggested excellent dispersion stability of the synthesized PB in PBS and DMEM, meeting the requirement for intravenous injection.

### 2.2 Antioxidant properties and enzyme-like activity test of PB

Post-SCI tissue may undergo a series of complex pathological changes, among which substantial ROS generation stands out [36]. Besides direct damage to neurons, ROS can also activate numerous inflammatory pathways to promote the accumulation of inflammatory cells at the site of injury, releasing more ROS and inflammatory factors, thereby creating a vicious cycle [36,37]. Therefore, it highlights the significance of regulating ROS levels in treating the secondary effect of SCI. PB nanozymes, with unique electron transfer properties, have been demonstrated to efficiently catalyze the decomposition of ROS [38,39], especially superoxide radicals (O_2_•^-^) and hydrogen peroxide (H_2_O_2_), enabling the transformation of these harmful molecules into relatively harmless H_2_O and O_2_. Given their scavenging ability of ROS, PB nanozymes play a fair role in various oxidative stress-induced diseases, including neurodegenerative diseases [23], osteoporosis [40], and ischemic brain damage [39]. On this basis, the present experiment delved into the study of the mimicry of PB of multi-enzyme activity and its efficacy in clearing various ROS. An investigation on the cytotoxic effect of PB was initiated to establish a reasonable range for its concentration. The results of CCK-8 assay indicated that PB was biocompatible and exhibited no significant impact on the viability of BMDMs over observation periods of 24, 48, and 72 h, even at a high concentration of 100 μg/ml (Fig. S2, A to C). Subsequently, our experiment set a gradient of PB concentrations from 3.125 μg/ml to 100 μg/ml to further evaluate its effect of scavenging ROS. Our study initially validated the anti-oxidative potential of PB through the ABTS radical scavenging assay. Co-incubation with PB significantly reduced the ABTS absorption at 734 nm (Fig. 1K), highlighting a positive correlation of ABTS radical scavenging capacity with the concentration of PB quantitatively (Fig. 1K, and Fig. S2D). Particularly, PB at 100 μg/ml achieved a maximum scavenging efficiency of 56.3 % ± 3.14 % (Fig. 1K, and Fig. S2D). Meanwhile, as revealed by the DPPH radical scavenging assay, there was a significant decrease in DPPH absorption at 517 nm post-PB treatment (Fig. 1L), with a scavenging capacity of 87.03 % ± 2.40 % at 100 μg/ml (Fig. 1L, and Fig. S2E). Then, biologically relevant ROS species, including H2O2, •OH, and O_2_•^-^, were employed to explore the ROS scavenging efficiency of PB at different concentrations. Consequently, PB could effectively eliminate these ROS species, and had enhanced efficiency with the increase of concentrations. At 100 μg/ml, PB showed the scavenging rates for H_2_O_2_, •OH, and O_2_•^-^ of 92.87 % ± 0.37 % (Figs. 1M), 69.66 % ± 2.16 % (Figs. 1N), and 87.20 % ± 0.57 % (Fig. 1O), respectively. Such remarkable scavenging ability might be attributed to its mimicry of natural enzymes (Fig. 1T), such as catalase (CAT), superoxide dismutase (SOD), peroxidase (POD), and glutathione peroxidase (GPx), rendering PB a potent tool for researching and treating oxidative stress-related diseases. As an SOD mimetic, PB could alleviate oxidative stress by converting superoxide anion radicals (O_2_•^-^) into O_2_ and H_2_O_2_. Furthermore, PB's mimicry of POD and CAT enzyme activities facilitated a decomposition of H_2_O_2_ into H_2_O and O_2_ to realize mitigated oxidative stress. In general, vascular loss and blood-spinal cord barrier disruption may occur during SCI, which may trigger inflammation, ischemia, and hypoxia to induce further damage to spinal cord tissues [41]. Therefore, the healing of SCI may be accelerated through a mechanism that simultaneously releases O_2_ and significantly scavenges ROS [10]. Supported by its POD and CAT enzyme mimetic activities releasing O_2_, PB may stimulate angiogenesis and potentially promote vascularization at the site of SCI. Furthermore, the GPx mimetic effect of PB may enable an effective conversion of harmful H_2_O_2_ into H_2_O and O_2_, further enhancing the defense against post-SCI oxidative stress. Thus, our study continued to clarify the multi-enzyme (e.g., CAT, SOD, POD, and GPx enzymes) mimetic activities of PB thoroughly. As a result, the activities of these mimetic enzymes correspondingly enhanced with an increase in PB concentration (Fig. 1, P to S). Especially at 100 μg/ml of PB, the mimetic activities of CAT, SOD, POD, and GPx were 13.28 ± 0.06 U/ml (Figs. 1P), 48.82 ± 4.57 U/ml (Figs. 1Q), 321.90 ± 0.17 U/ml (Figs. 1R), and 56.83 ± 0.77 U/ml (Fig. 1S), respectively. Overall, PB, possessing multi-enzyme mimetic activities, could effectively scavenge various types of ROS, potentially playing a protective role in the highly oxidative stress microenvironment of SCI.

### 2.3 Recruitment roles of different cells after SCI indicated by scRNA-seq

**Fig. 2:**
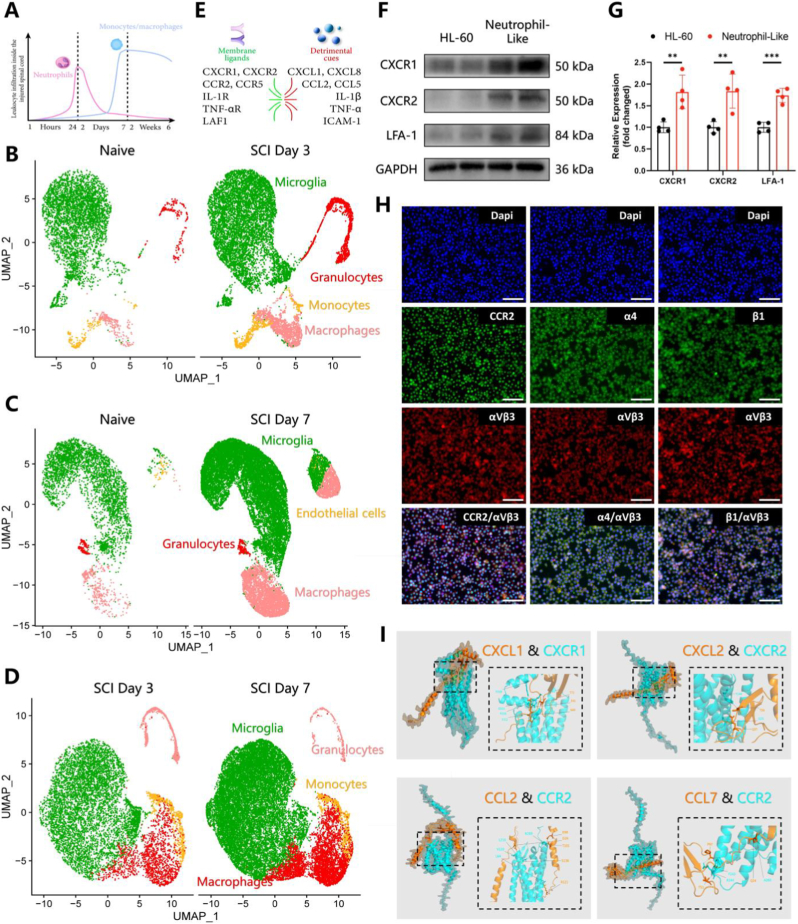
Spatiotemporal inflammatory targeting concepts-sequential recruitment of neutrophils and macrophages to the inflamed sites of SCI. (A) Schematic diagram of the timing for post-SCI sequential recruitment of neutrophils and macrophages, demonstrating “temporal” targeting capability. (B to D) The quantity and proportion of different cells at 0, 3, 7 days after SCI (Naive, post-SCI Day 3, and post-SCI Day 7) by scRNA-seq. (E) Schematic diagram illustrating the “spatial” targeting capability through the pairing of receptors (CXCR1, CXCR2, CCR2, CCR5, etc.) on neutrophils and macrophages with inflammatory factors (CXCL1, CXCL8, CCL2, CCL5, etc.) at the site of SCI. (F and G) Western blot of key inflammatory chemotactic proteins CXCR1, CXR2 and LFA-1. Human promyelocytic leukemia (HL-60) cells treated with DMSO (n = 4, mean ± SD). (H) Immunofluorescence of surface markers on macrophages related to recruitment and inflammatory chemotaxis (CCR2, Integrins α4, Integrins β1 and Integrins αvβ3) (n = 4, mean ± SD). (I) Computer simulation image of protein docking between receptor-ligand. n represents the number of biologically independent samples. P values are shown in graphs with significance levels denoted as ∗P < 0.05, ∗∗P < 0.01, and ∗∗∗P < 0.001. Scale bars, 200 μm (H).

It is a great challenge to apply targeted therapy for SCI due to its complex pathological process [42]. To address this, this study proposed “spatiotemporal delivery”, a novel concept aiming at achieving precise treatment for SCI. For the first time, this concept underscores the importance of a deep understanding of post-SCI sequential recruitment of different cells for delivery in the “temporal” dimension. Sequential recruitment of peripheral immune cells is particularly crucial in the early inflammatory response following SCI, mainly involving three types [43]: (1) neutrophils, as the first batch of inflammatory cells to reach the site of injury, may peak within 1 day post-injury (Fig. 2A); (2) monocytes/macrophages, following closely, can peak on the 7 days post-injury (Fig. 2A), with significant phagocytic capability; (3) lymphocytes gradually infiltrate the site of injury, arriving alongside macrophages, and secrete cytokines at the damage center. Notably, lymphocytes are relatively fewer in number compared to other cell types. Therefore, post-SCI secondary inflammation primarily involves sequential recruitment and activation of neutrophils and macrophages (Fig. 2A). This view has been supported by additional research on post-SCI sequential recruitment of cells in human [44]. Within 1–3 days post-SCI, neutrophils have been found to enter the spinal cord through hemorrhage or exudation, peaking in number during this period, and can be detected for up to 10 days post-SCI [44]. Simultaneously, the injured tissue may be detected with a significant number of microglia and some monocytes/macrophages within this period [44]. Activated microglia, some monocytes/macrophages, and a large number of phagocytic macrophages persist for weeks to months post-SCI [44]. In our study, with the use of scRNA-seq dataset (GSE205037) [45], this process was further validated by analyzing results at multiple time points, i.e., naive, 3 days post-SCI (post-SCI Day 3), and 7 days post-SCI (post-SCI Day 7). As a result, the proportion of granulocytes increased significantly at post-SCI Day 3 compared to the naive group, with a significant decrease at post-SCI Day 7 (Fig. 2B–D, and Fig. S3, A to C). Meanwhile, the proportion of macrophages increased at post-SCI Day 3 and further remarkably increased at post-SCI Day 7 (Fig. 2B–D, and Fig. S3, A to C). These findings provide crucial evidence for considering cell sequential recruitment in post-SCI targeted delivery therapy. Accordingly, a nanozyme platform camouflaged with a hybrid membrane of Nm and Mm was further developed, inspired by the post-SCI sequential recruitment of neutrophils and macrophages. The hybrid cell membrane strategy has been extended to various combinations of cell types, including erythrocyte-platelet [46], erythrocyte-macrophage [47], macrophage-cancer cell [48], dendritic cell-cancer cell [49], etc. However, this hybrid membrane strategy (i.e., neutrophils and macrophages), to our knowledge, has not yet been used for SCI treatment. By inheriting relevant components (e.g., proteins, antigens, carbohydrates, etc.) from various cell membranes, this hybrid cell membrane can exhibit diverse physicochemical properties and cell-specific functionalities [48,49]. This strategy is expected to preserve the characteristics of post-SCI sequential recruitment of neutrophils and macrophages, thereby achieving long-term targeted delivery on the “temporal” dimension.

### 2.4 Detection of post-SCI inflammatory factors and identification of surface markers on neutrophil-like cells and macrophages

Targeted delivery at the “spatial” dimension is mediated by the unique inflammatory microenvironment perceived by neutrophils and macrophages following SCI (Fig. 2E). In the initial stages of SCI, a plethora of chemokines, including C-X-C motif chemokine (CXCL) 1/2/3/4/5/6/7/8, are massively released to effectively recruit neutrophils as the most sensitive chemotactic cells that rapidly migrate towards the region of inflammation [50]. This mechanism of neutrophil recruitment has been leveraged in targeted treatment for neural and pulmonary inflammation [51]. However, the efficiency of neutrophils in experimental research and clinical application is significantly compromised by their short lifespan, undergoing apoptosis within 6–12 h from isolation, and the minuscule amount of membrane protein extracted from a large number of neutrophils [52]. In response to these limitations, aiming at enhancing the production efficiency, this study selected a cost-effective, high-efficiency, reproducible, and biosafe solution for preparing neutrophil-like membranes, utilizing human promyelocytic leukemia (HL-60) cells treated with dimethyl sulfoxide (DMSO), which can differentiate into neutrophil-like cells and undergo subculture over multiple generations [10,52]. After DMSO stimulation, this experiment detected an obviously increased expression of CXCR1, CXCR2, and LFA-1 (Fig. 2F and G), key inflammatory chemotactic proteins that are crucial in post-SCI chemotactic function of neutrophils. Additionally, RAW264.7 murine macrophage line was utilized to collect Mm. Furthermore, immunofluorescence was employed to investigate CC-chemokine receptor 2 (CCR2), Integrins α4, β1, and αvβ3, related to the aforementioned processes, given the crucial role of macrophage surface proteins in signal transduction and recruitment (Fig. 2H). CCR2 can recognize the highly expressed CC-chemokine ligand 2 (CCL2) in the inflammatory microenvironment of SCI, thus participating in recruiting macrophages to the site of injury, while integrins α4, β1, and αvβ3 are involved in macrophage migration and adhesion that can enter the inflamed tissue [53]. Various chemokines, including members of the CXCL and CCL families, function significantly in recruiting neutrophils and macrophages to the site of SCI. Therefore, this study examined the post-SCI gene expression of the CXCL and CCL families. Consequently, CXCL1/2/3/5/9/10/12/13/14/16/17 from the CXCL family and CCL2/3/4/5/6/7/8/9/11 from the CCL family increased to varying degrees at different time points after injury (Fig. S4, A and B), providing solid *in vivo* evidence for the chemotactic capability of the hybrid membrane of neutrophils and macrophages. Furthermore, through molecular docking, we showcased the pairing outcomes between CXCL1 and CXCR1, CXCL2 and CXCR2, CCL2 and CCR2, CCL7 and CCR2, as well as other receptor-ligand interactions (Fig. 2I, and Fig. S4, C to G). Therefore, our NMm camouflaged system could be precisely driven by inflammation, facilitating targeted delivery on the “spatial” dimension.

### 2.5 Preparation and characterization of NMm-pPB-siRNA^TRAF6^

**Fig. 3:**
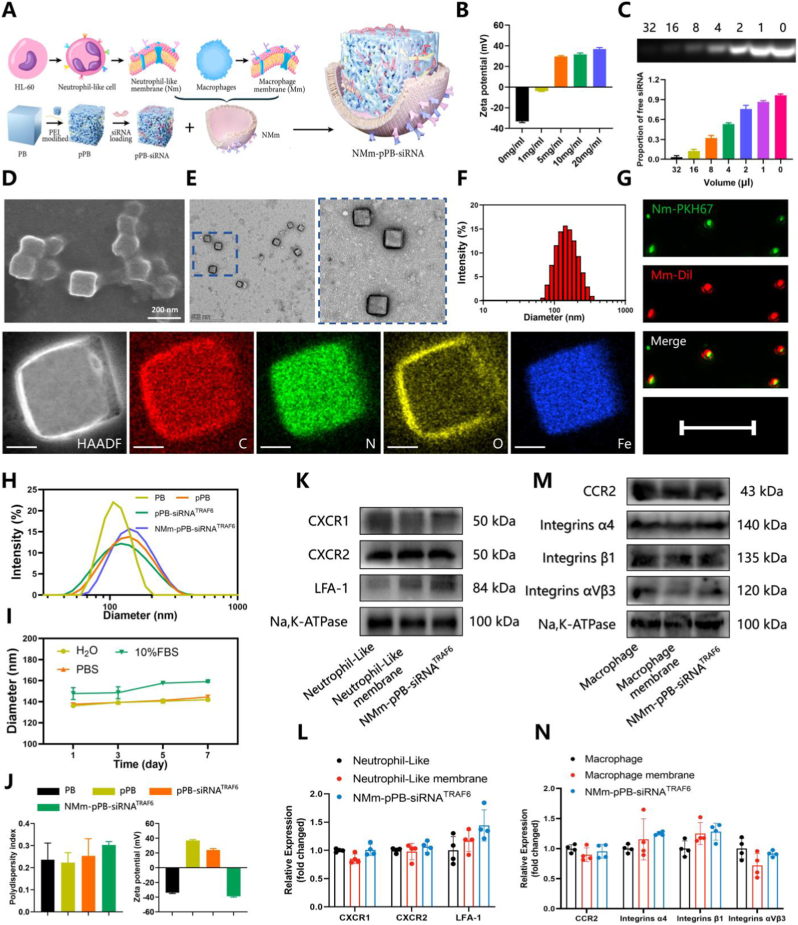
Preparation and characterization of NMm-pPB-siRNA^TRAF6^. (A) Schematic diagram of NMm-pPB-siRNA^TRAF6^ synthesis, including the extraction of Nm and Mm; PEI modification of PB (pPB); pPB loaded with siRNA^TRAF6^ (pPB-siRNA^TRAF6^) and hybrid membrane coated (NMm-pPB-siRNA^TRAF6^). (B) Zeta potential of PB reacted with PEI at different concentrations (n = 3, mean ± SD). (C) Characterization of pPB-siRNA_Cy5_ using agarose gel electrophoresis at different volumes of PB with 5 μL siRNA_Cy5_ (20 μM) (n = 3, mean ± SD). (D) SEM images of NMm-pPB-siRNA^TRAF6^. (B) TEM images with corresponding elemental mappings of NMm-pPB-siRNA^TRAF6^. (C) Diameters of NMm-pPB-siRNA^TRAF6^. (G) Confocal images of the overlapped fluorescence on NMm-pPB-siRNA^TRAF6^ indicating the presence of both Nm-PKH67 and Mm-Dil. (H) Diameters of PB, pPB, pPB-siRNA^TRAF6^ and NMm-pPB-siRNA^TRAF6^ (n = 3, mean ± SD). (I) Stability of NMm-pPB-siRNA^TRAF6^ in H_2_O, PBS, and 10 % FBS for seven days (n = 3, mean ± SD). (J) PDI and zeta potential of PB, pPB, pPB-siRNA^TRAF6^ and NMm-pPB-siRNA^TRAF6^ (n = 3, mean ± SD). (K and L) Western blot analysis and relative quantification for the retention of neutrophil-like cell membrane proteins (CXCR1, CXCR2 and LFA-1) (n = 4, mean ± SD). (M and N) Western blot analysis and relative quantification for the retention of Mm proteins (CCR2, Integrins α4, Integrins β1 and Integrins αvβ3) (n = 4, mean ± SD). n represents the number of biologically independent samples. P values are shown in graphs with significance levels denoted as ∗P < 0.05, ∗∗P < 0.01, and ∗∗∗P < 0.001. Scale bars, 200 nm (D), 500 nm (E), and 10 μm (G).

Fig. 3A illustrates the structure and fabrication of NMm-pPB-siRNA, encompassing a PB core, siRNA, and a hybrid membrane of neutrophil-like and macrophage cells. Through electrostatic self-assembly, cationic PEI adhered to the PB surface, forming PEI-modified PB (pPB). At 20 mg/ml of PEI, the content saturated in the surface-NH2 group, and the total charge approached its maximum positive potential, indicating the optimal binding concentration (Fig. 3B), with the zeta potential of pPB of 36.97 ± 1.44 mV (Fig. 3B). TEM images of pPB revealed its regular cubic structure, indicating no alteration of the original structure of PB by PEI modification (Fig. S5A). Subsequently, pPB loaded with siRNA^TRAF6^ (pPB-siRNA^TRAF6^) was successfully prepared by increasing the amount of pPB. Agarose gel electrophoresis indicated a gradual reduction in free siRNA content (Fig. 3C), with the highest binding capacities of pPB and siRNA being 32 μL (at 1 mg/ml of PB) and 5 μL (20 μM), respectively (Fig. 3C). Then, we fabricated NMm-pPB-siRNA^TRAF6^ nanoparticles encapsulated with Nm and Mm via physical extrusion. SEM (Fig. 3D) and TEM (Fig. 3E) images showed spontaneous organization of the cell membrane onto the surface of pPB-siRNA^TRAF6^ nanoparticles after physical extrusion, forming a core-shell structure. The uniform membrane encapsulation presented a bright halo, thoroughly covering the pPB-siRNA^TRAF6^ nanoparticles. Moreover, both cell membranes were successful loaded onto the NMm-pPB-siRNA^TRAF6^, as confirmed by the overlapping fluorescence of PKH67-labeled Nm and Dil-labeled Mm (Fig. 3G). Dynamic light scattering (DLS) analysis revealed that the average particle sizes of PB, pPB, pPB-siRNA^TRAF6^, and NMm-pPB-siRNA^TRAF6^ were 110.6 ± 4.80, 129.9 ± 10.38, 119.8 ± 2.36, and 153.2 ± 6.68 nm, respectively (Fig. 3, F and H, and Fig. S5, B and C). These data suggested a slight increase in particle size after PEI modification and a slight decrease in average particle size of pPB-siRNA^TRAF6^ compared to pPB, potentially attributable to changes in surface charge leading to reduced strength of ionic interactions in solution. The encapsulation of the cell membrane resulted in larger nanoparticle sizes for NMm-pPB-siRNA compared to pPB and pPB-siRNA (Fig. 3, F and H, and Fig. S5, B and C). The measurement of PDI also ducmented their excellent dispersibility in solution (Fig. 3J). Additionally, the zeta potentials of PB, pPB, pPB-siRNA^TRAF6^, and NMm-pPB-siRNA^TRAF6^ were −33.93 ± 1.16, 36.83 ± 1.50, 24.17 ± 1.86, and −38.77 ± 1.15, respectively (Fig. 3J), confirming the successful coating of various layers onto the PB surface on the basis of changes in positive and negative zeta potentials. Furthermore, the cell membrane, composed of a mixture of lipids, proteins, and carbohydrates, can interact directly with the surrounding environment to mediate cell recognition, signal transduction, and adhesion [53]. In our study, we continued to determine whether the extracted neutrophil-like and macrophage cell membranes as well as their encapsulated nanoparticles can retain essential proteins from the source cells, demonstrating the potential for neutrophil and macrophage “spatiotemporal delivery”. Western blot was also employed to detect the protein expression of CXCR1, CXCR2, and LFA-1, surface molecules of neutrophil-like cells, neutrophil-like cell membrane, and NMm-pPB-siRNA^TRAF6^, all of which were transferred to the surface of NMm-pPB-siRNA^TRAF6^ after cell membrane extrusion (Fig. 3K and L). Similarly, proteins such as CCR2, Integrins α4, Integrins β1, and Integrins αVβ3 on the macrophage cell membrane surface were retained by NMm-pPB-siRNA^TRAF6^ (Fig. 3, M and N). Collectively, cell membrane coating could replicate the composition of natural cell membranes to preserve key proteins, laying a foundation for “spatiotemporal delivery".

### 2.6 In vitro targeting of inflammatory macrophages and nerve cells

**Fig. 4:**
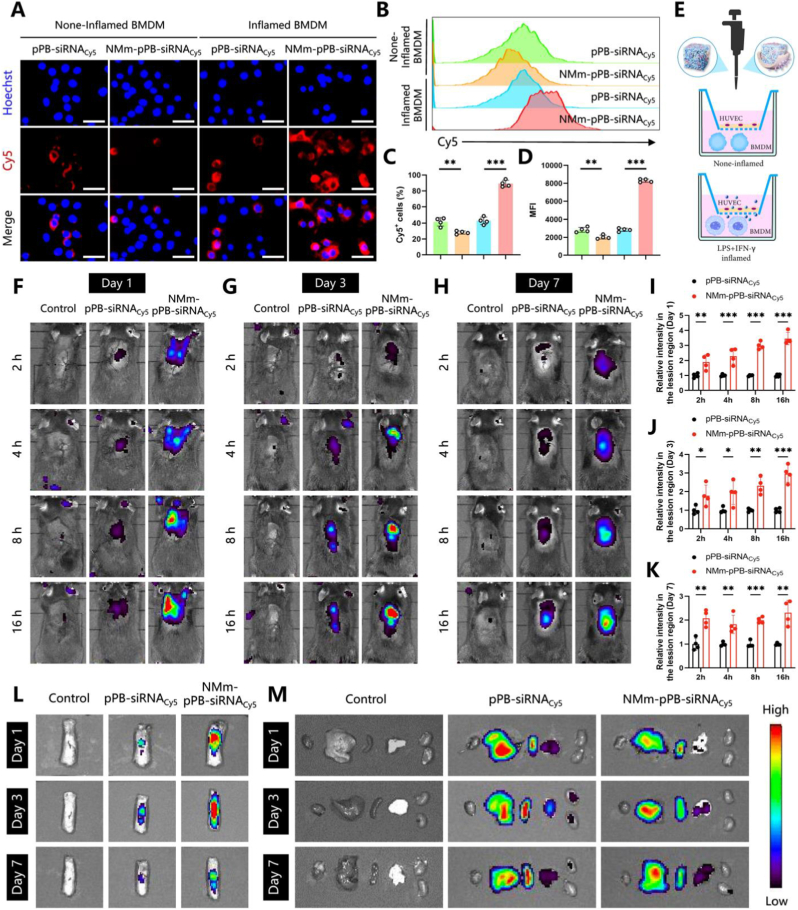
NMm-pPB-siRNA^TRAF6^ targeted inflammatory macrophages and neuronal cells *in vitro* and injured spinal cord tissues *in vivo*. (A) Representative confocal images of BMDM incubated with pPB-siRNA_Cy5_ and NMm-pPB-siRNA_Cy5_ under stimulation using LPS and IFN-γ (inflamed BMDMs) or without stimulation using LPS and IFN-γ (none-inflamed BMDMs). (B–D) Fluorescence of pPB-siRNA_Cy5_ and NMm-pPB-siRNA_Cy5_ under none-inflamed or inflamed BMDMs measured by FCM (n = 4, mean ± SD). (E) Schematic diagram to measure the targeting ability of pPB-siRNA_Cy5_ and NMm-pPB-siRNA_Cy5_ in none-inflamed or inflamed BMDMs. (F–H) Fluorescence images of pPB-siRNA_Cy5_ and NMm-pPB-siRNA_Cy5_ in SCI mice. On Day 1, 3, and 7 post-SCI, pPB-siRNA_Cy5_ and NMm-pPB-siRNA_Cy5_ were injected through the tail vein intravenously, followed by imaging at 2-, 4-, 8-, and 16-h post-injection using the *in vivo* imaging system. (I–K) Quantification of the fluorescence intensity of lesions in SCI mice (n = 4, mean ± SD). (L) Representative IVIS images of the spinal cord in mice treated with pPB-siRNA_Cy5_ and NMm-pPB-siRNA_Cy5_ on post-SCI Day 1,3, and 7., with the results of quantitative analysis of corresponding fluorescence intensity shown in Fig. S7 (n = 4, mean ± SD). (M) Representative IVIS images of the major organs (from left to right: heart, liver, spleen, lungs, and kidneys) in mice treated with pPB-siRNA_Cy5_ and NMm-pPB-siRNA_Cy5_ on post-SCI Day 1,3, and 7, with the results of quantitative analysis of corresponding fluorescence intensity shown in Fig. S7 (n = 4, mean ± SD). n represents the number of biologically independent samples. P values are shown in graphs with significance levels denoted as ∗P < 0.05, ∗∗P < 0.01, and ∗∗∗P < 0.001. Scale bars, 50 μm (A).

We have verified that PB at 100 μg/mL exhibited no significant cytotoxicity in BMDMs (Fig. S2, A to C). To further explore the effects of pPB, pPB-siRNA^TRAF6^, and NMm-pPB-siRNA^TRAF6^ on cell viability *in vitro*, CCK-8 assays revealed that these nanoparticles (with PB at 100 μg/mL) did not significantly affect the cell viability in BMDMs and PC12 cells (a common neuronal cell line) over periods of 24, 48, and 72 h (Fig. S6, A and B). This study further employed Cy5-labeled siRNA (siRNA_Cy5_) to investigate the targeting capability of NMm-pPB-siRNA_Cy5_ towards inflammatory macrophages, with the use of human umbilical vein endothelial cells (HUVECs) as the cell type that should not uptake nanoparticles in a Transwell model simulating SCI [10]. Due to receptor (e.g., CXCR1, CXCR2, and CCR2 on neutrophils and macrophages)-chemokine interaction, NMm-pPB-siRNA_Cy5_ may enable an effective targeting of inflammatory M1-type macrophages. As expected, analyses using confocal laser scanning microscopy (CLSM) and flow cytometry (FCM) showed a significant increase in the uptake of NMm-pPB-siRNA_Cy5_ by inflamed BMDMs compared to none-inflamed BMDMs (Fig. 4A–D). Therefore, the targeting of NMm might be driven by the binding of cell membrane surface receptors to inflammatory chemokines in an inflammatory environment. Moreover, the uptake of NMm-pPB-siRNA_Cy5_ in inflamed BMDMs was significantly higher than that of pPB-siRNA_Cy5_ (Fig. 4A–D), further demonstrating enhanced targeting of NMm coating towards inflamed BMDMs. Interestingly, the uptake of NMm-pPB-siRNA_Cy5_ in none-inflamed BMDMs was lower than that of pPB-siRNA_Cy5_ (Fig. 4A–D). This result may be explained by the cell membrane coating technology preventing the phagocytosis and clearance of nanoparticles by macrophages to some extent [54], as well as the positive charge of PEI modification possibly enhancing the cellular uptake of nanoparticles. Similar results were observed in inflammatory PC12 cells (Fig. S6C). Altogether, NMm-pPB-siRNA^TRAF6^ nanozyme platform designed in our study exhibited excellent inflammatory targeting capability *in vitro*, driven by the inflammatory environment, which can adapt to the need of post-SCI inflammatory microenvironment.

### 2.7 Assessment of the capability of NMm-pPB-siRNA^TRAF6^ targeting the injured spinal cord

To effectively address the complex post-SCI inflammatory environment, this study proposed a concept of “spatiotemporal delivery”. Initially, on the “temporal” dimension, this study investigated and deciphered post-SCI sequential recruitment of neutrophils and macrophages. Subsequently, in the “spatial” dimension, it underscored the sensing and targeting of the inflammatory microenvironment within the region of SCI by neutrophils and macrophages. To validate whether the NMm-camouflaged nanozyme platform could achieve this “spatiotemporal delivery”, a mouse model constructed by injecting pPB-siRNA_Cy5_ and NMm-pPB-siRNA_Cy5_ into the tail veins of mice with SCI, and the IVIS technology was employed to assess their *in vivo* distribution and the intensity of Cy5 fluorescence in major organs. Notably, based on the results of scRNA-seq described above, the injection was implemented on the first day (Day 1), third day (Day 3), and seventh day (Day 7) post-SCI to mimic the sequential recruitment of neutrophils and macrophages. After injections, IVIS detection was conducted at 2 h, 4 h, 8 h, and 16 h, respectively. Consequently, compared to the pPB-siRNA_Cy5_ group, at post-SCI Day 1, the NMm-pPB-siRNA_Cy5_ group showed relatively higher fluorescence intensity at the injury site at all time points (Fig. 4F–K), with similar results observed at post-SCI Day 3 and post-SCI Day 7 (Fig. 4F–K). Therefore, the NMm system possesses significant capabilities of “temporal” targeting, achieving targeted delivery from 1 to 7 days post-SCI, meeting the therapeutic needs at different time points of secondary SCI. Furthermore, through analyses with IVIS at the final time point after spinal cord dissection, the fluorescence intensity at the NMm-pPB-siRNA_Cy5_ treated site of SCI was significantly higher than that of the pPB-siRNA_Cy5_ treated group (Fig. 4L, and Fig. S7, A to C). Subsequently, through the dissection and quantitative analysis of major organs in mice, pPB-siRNA_Cy5_ and NMm-pPB-siRNA_Cy5_ were observed to be primarily accumulated in the liver, followed by the spleen and lungs (Fig. 4M, and Fig. S7, A to C), confirming to the *in vivo* distribution characteristics of most nanoparticles. Interestingly, compared to the pPB-siRNA_Cy5_ injection, NMm-pPB-siRNA_Cy5_ injection reduced signals in the liver and lungs on post-SCI Day 1 (Fig. 4M, and Fig. S7, A to C); reduced signals in the spleen, lungs, and kidneys on post-SCI Day 3 (Fig. 4M, and Fig. S7, A to C); and reduced signals in the spleen on post-SCI Day 7 (Fig. 4M, and Fig. S7, A to C). This could be attributed to the better targeting of NMm-pPB-siRNA_Cy5_ to the spinal cord, thereby reducing distribution in other organs. To further substantiate the “spatiotemporal delivery” capability at the cellular level, we performed immunofluorescence co-localization analysis on longitudinal spinal cord sections harvested 16 h after intravenous administration on days 1, 3, and 7 post-SCI. Consistent with the infiltration dynamics of immune cells, the NMm-pPB-siRNA_Cy5_ (pseudo-colored yellow) effectively accumulated in the lesion area upon injection during the acute to subacute phases (Day 1 to Day 7) (Fig. S8A). Notably, the nanoparticles exhibited co-localization with both Iba1+ microglia/macrophages (red) and NeuN + neurons (green) across these time points (Fig. S8C), demonstrating that the hybrid membrane coating effectively mimics the recruitment behavior of neutrophils and macrophages to target the inflammatory microenvironment. In contrast, the pPB-siRNA_Cy5_ group showed significantly reduced retention and cellular uptake within the lesion. Quantitative analysis further confirmed that the fluorescence intensity of the NMm-pPB-siRNA_Cy5_ group was significantly higher than that of the pPB-siRNA_Cy5_ group at all evaluated time points (Fig. S8, A and B). Collectively, these multi-time-point cellular level data provide evidence for the ‘spatiotemporal delivery' concept: the nanozyme platform maintains consistent temporal retention during the disease progression and achieves precise spatial coverage of the key cellular components within the lesion.

### 2.8 In vitro and *in vivo* gene silencing efficiency

Our subsequent experiments were performed to evaluate the target silencing capability of NMm-pPB-siRNA^TRAF6^ nanozyme platform against TRAF6. Three siRNA sequences: siRNA^TRAF6^_002, siRNA^TRAF6^_003, and siRNA^TRAF6^_004 (Fig. S9A) were designed to ensure high transfection efficiency. Compared to the control group siRNA^NC^, the expression levels of TRAF6 were reduced to 0.593 ± 0.120, 0.102 ± 0.047, and 0.774 ± 0.030 times by siRNA^TRAF6^_002, siRNA^TRAF6^_003, and siRNA^TRAF6^_004, respectively, with the highest efficiency of silencing observed in siRNA^TRAF6^_003 (Fig. S9B). Therefore, siRNA^TRAF6^_003, unless specifically indicated, was selected for subsequent investigations. To further verify the silencing effect of NMm-pPB-siRNA^TRAF6^ nanozyme platform on TRAF6 expression, BMDMs were incubated with various samples (i.e., free siRNA^TRAF6^, PB, pPB-siRNA^NC^, pPB-siRNA^TRAF6^, and NMm-pPB-siRNA^TRAF6^), followed by quantitative analysis of TRAF6 mRNA levels through qRT-PCR. Both pPB-siRNA^TRAF6^ and NMm-pPB-siRNA^TRAF6^ significantly reduced TRAF6 mRNA levels (Fig. S9C). It can be speculated that siRNA could form RISC in macrophages, thereby decreasing the expression of target gene, inspiring our further exploration of the capability of this nanozyme platform to silence TRAF6 levels *in vivo*. In *vivo* experiments, the above samples were injected intravenously into a mouse model of SCI on days 1, 3, 5, and 7 after modeling. After 7 days of treatment, spinal cord tissues were collected for detecting TRAF6 expression levels through immunofluorescence, with the use of NeuN to mark neurons and Iba1 to mark macrophages/microglia. It was observed with significant reduction in TRAF6 fluorescence intensity in neurons in the pPB-siRNA^TRAF6^ and NMm-pPB-siRNA^TRAF6^ treatment groups (Fig. S10, A and B), with the effect of NMm-pPB-siRNA^TRAF6^ surpassing that of pPB-siRNA^TRAF6^ (Fig. S10, A and B). This could be attributed to the specific “spatiotemporal delivery” provided by the NMm system *in vivo*. Similar results were observed in the analysis of macrophages/microglia (Fig. S11, A and B), indicating that our NMm-pPB-siRNA^TRAF6^ could effectively target and silence TRAF6 expression in neurons and macrophages/microglia within spinal cord tissues. Finally, WB results of TRAF6 protein levels were consistent with those of immunofluorescence (Fig. S12, A and B). Compared to the Sham group, the levels of TRAF6 were significantly elevated in the SCI group, but remarkably reduced in the pPB-siRNA^TRAF6^ and NMm-pPB-siRNA^TRAF6^ treatment group, whereas free siRNA and PB did not show this effect (Fig. S12, A and B). These trends further supported the efficacy and potential of our nanozyme platform in targeting and silencing TRAF6.

### 2.9 NMm-pPB-siRNA^TRAF6^ alleviate oxidative stress *in vitro* and *in vivo*

**Fig. 5:**
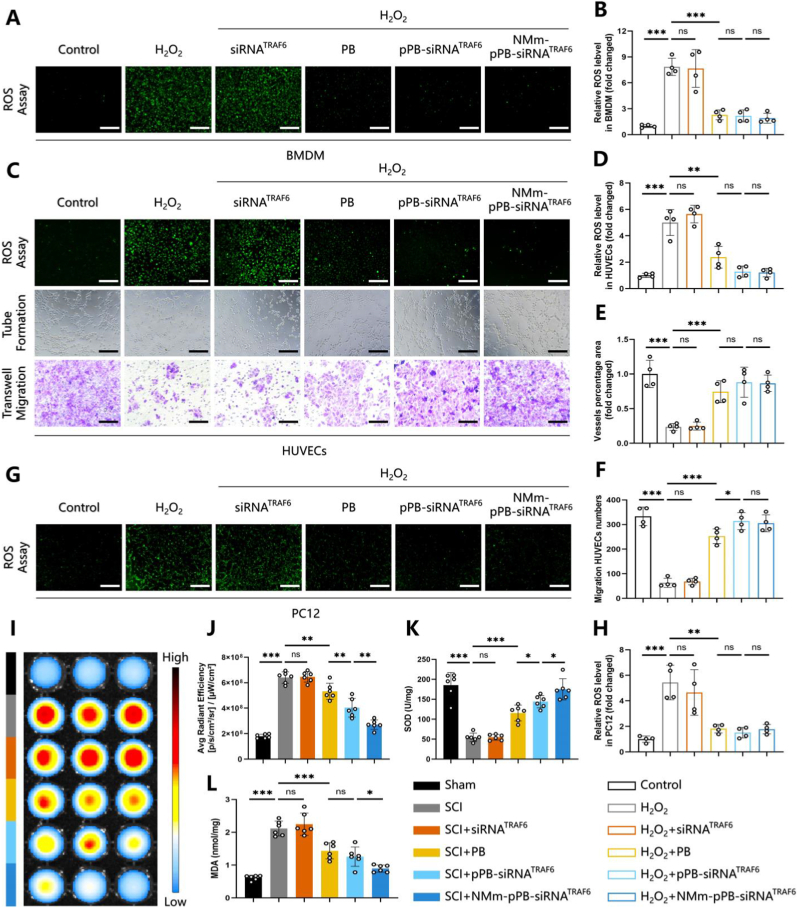
NMm-pPB-siRNA^TRAF6^ alleviated oxidative stress *in vitro* and *in vivo*. (A and B) DCFH-DA fluorescence microscopy of BMDM incubated with H_2_O_2_, H_2_O_2_ and siRNA^TRAF6^, H_2_O_2_ and PB, H_2_O_2_ and pPB-siRNA^TRAF6^, or H_2_O_2_ and NMm-pPB-siRNA^TRAF6^ (n = 4, mean ± SD). (C and D) DCFH-DA fluorescence microscopy of HUVECs incubated with H_2_O_2_, H_2_O_2_ and siRNA^TRAF6^, H_2_O_2_ and PB, H_2_O_2_ and pPB-siRNA^TRAF6^, or H_2_O_2_ and NMm-pPB-siRNA^TRAF6^ (n = 4, mean ± SD). (E) Measurement of vessel percentage area by the angiogenic analysis plugin on Image J (n = 4, mean ± SD). (F) Measurement of migrated numbers of HUVECs by Image J (n = 4, mean ± SD). (G and H) DCFH-DA fluorescence microscopy of PC12 incubated with H_2_O_2_, H_2_O_2_ and siRNA^TRAF6^, H_2_O_2_ and PB, H_2_O_2_ and pPB-siRNA^TRAF6^, or H_2_O_2_ and NMm-pPB-siRNA^TRAF6^ (n = 4, mean ± SD). (I and J) Detection of the fluorescence intensity of DHE in spinal cord tissues after treatment on post-SCI Day 7 using the IVIS system (n = 6, mean ± SD). (K and L) Detection of SOD and MDA levels in spinal cord tissues after treatment on post-SCI Day 7 (n = 6, mean ± SD). n represents the number of biologically independent samples. P values are shown in graphs with significance levels denoted as ∗P < 0.05, ∗∗P < 0.01, and ∗∗∗P < 0.001. Scale bars, 300 μm (A to C).

ROS and oxidative stress are two pivotal players in the pathogenesis of SCI, and SCI may be improved to some extent by mitigating oxidative stress [55]. Our previous experiments have confirmed the strong antioxidant enzyme–mimicking activities of PB (including CAT, SOD, POD, and GPx) and its potent ROS-scavenging capability. Building on these findings, we further investigated the ability of the NMm-pPB-siRNA TRAF6 nanozyme platform to protect the injured spinal cord from oxidative damage, and conducted corresponding *in vitro* oxidative stress experiments to validate its antioxidative performance. After treatment of BMDMs (Fig. 5A and B), PC12 (Fig. 5G and H), and HUVECs (Fig. 5C and D) with H_2_O_2_, a post-SCI oxidative stress environment was stimulated to assess ROS levels using DCFH-DA staining. Compared to the H_2_O_2_ treatment group, PB, pPB-siRNA^TRAF6^, and NMm-pPB-siRNA^TRAF6^ significantly reduced ROS levels (Fig. 5A–H), with all the three treatments demonstrating comparable efficacy in mitigating cellular oxidative stress (Fig. 5A–H). Therefore, our constructed NMm-pPB-siRNA^TRAF6^ could effectively control oxidative stress across different cell types, exhibiting an ability to adapt to the complex cellular damage environment of SCI [56]. Generally, following SCI, there may be vascular damage and dysfunction of blood-spinal cord barrier, in turn triggering inflammation and ischemia, potentially causing extensive spinal cord neural tissue damage [57]. The elimination of ROS and continuous release of oxygen may be feasible solutions for promoting endothelial cell tubule formation and angiogenesis [10]. Furthermore, our study also evaluated the impact of NMm-pPB-siRNA^TRAF6^ on the migration and tube formation abilities of HUVECs under oxidative stress. Consequently, compared to the H_2_O_2_ treatment, NMm-pPB-siRNA^TRAF6^ significantly improved the migration and tube formation abilities of HUVECs (Fig. 5C–F, and Fig. S13, A and B). Following *in vitro* confirmation of the anti-oxidative efficacy, we further explored the capability of NMm-pPB-siRNA^TRAF6^ to control post-SCI oxidative stress *in vivo*. The fluorescent probe DHE was adopted to detect ROS in spinal cord tissue and the IVIS system to measure the fluorescence levels of DHE products, which are directly proportional to the levels of ROS in the spinal cord tissue. It was found that the levels of ROS significantly increased after SCI, but reduced after treatments with PB, pPB-siRNA^TRAF6^, and NMm-pPB-siRNA^TRAF6^ (Fig. 5I and J). Among these, the NMm-pPB-siRNA^TRAF6^ group showed the lowest fluorescence levels of DHE products (Fig. 5I and J), possibly attributable to its *in vivo* targeting ability and silencing effect on TRAF6. Additionally, measurement of SOD (anti-oxidative capacity) and MDA (the degree of lipid peroxidation and oxidative stress in tissue) levels in spinal cord further demonstrated the superior anti-oxidative stress capability of NMm-pPB-siRNA^TRAF6^ (Fig. 5, K and L). Overall, NMm-pPB-siRNA^TRAF6^ could serve as a promising ROS scavenger for treating SCI.

### 2.10 NMm-pPB-siRNA^TRAF6^ enhanced autophagy in neurons and macrophages after SCI

**Fig. 6:**
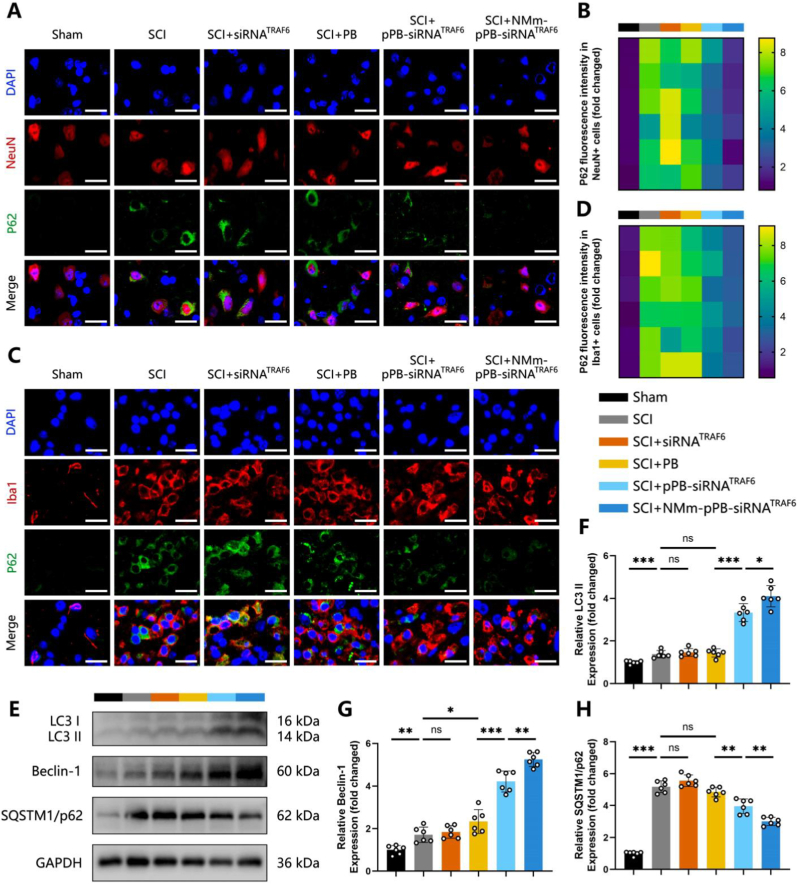
NMm-pPB-siRNA^TRAF6^ enhanced autophagy in neurons and macrophages. (A and B) Double-immunofluorescence staining and quantitative fluorescence intensity analysis of P62 and NeuN in spinal cords of the Sham, SCI, SCI + siRNA^TRAF6^, SCI + PB, SCI + pPB-siRNA^TRAF6^, SCI + NMm-pPB-siRNA^TRAF6^ groups on post-SCI Day 7 (n = 6, mean ± SD). (C and D) Double-immunofluorescence staining and quantitative fluorescence intensity analysis of P62 and Iba1 in spinal cords of the Sham, SCI, SCI + siRNA^TRAF6^, SCI + PB, SCI + pPB-siRNA^TRAF6^, SCI + NMm-pPB-siRNA^TRAF6^ groups on post-SCI Day 7 (n = 6, mean ± SD). (E to H) Western blot analysis and relative quantification of LC3, Beclin-1 and SQSTM1/P62 protein levels in the spinal cords of the Sham, SCI, SCI + siRNA^TRAF6^, SCI + PB, SCI + pPB-siRNA^TRAF6^, SCI + NMm-pPB-siRNA^TRAF6^ groups on post-SCI Day 7 (n = 6, mean ± SD). n represents the number of biologically independent samples. P values are shown in graphs with significance levels denoted as ∗P < 0.05, ∗∗P < 0.01, and ∗∗∗P < 0.001. Scale bars, 20 μm (A and C).

Antioxidants and nanozymes effective in eliminating excess ROS both extracellularly and within the cytoplasm. These two representative substances have been extensively applied in the research and treatment of SCI and other neuroinflammatory diseases in recent decades [9,24,58]. However, a commonly overlooked aspect is that they do not target the root source of ROS generation. In other words, the intracellular levels of ROS may quickly revert to their original state once these catalysts are expelled [25], implying that ROS is just temporarily cleared by such treatments, without addressing the root cause. ROS may be incessantly generated in such a continuous presence of dysfunctional cells (pyroptosis neurons and inflammatory M1 macrophages), negating the initial therapeutic effects. It underlines the necessity for more comprehensive strategies to correct cellular dysfunction, rather than controlling ROS merely. Autophagy refers to a cellular process that degrades and recycles damaged or unnecessary cellular components via the lysosomal pathway [26,59]. Our previous studies have documented the crucial role of autophagy in the recovery from SCI by promoting the clearance of damaged cells and cellular debris [60,61]. Notably, TRAF6 has been highly concerned regarding their role in the response of the CNS to traumatic injuries [30]. In prior rat models of traumatic brain injury and ischemic stroke, there was a significant increase in the level of TRAF6, which inhibited autophagy and accelerate the disease progression [[31], [32], [33], [34]]. On these bases, our study intended to develop an NMm-pPB-siRNA^TRAF6^ nanozyme platform to silence TRAF6, thereby restoring autophagy and fundamentally eliminating dysfunctional cells for effective post-SCI repair. Our first step aimed to examine the expression levels of autophagy-related proteins (Beclin-1 and LC3) and autophagy substrate proteins (SQSTM1/p62) on post-SCI Day 7. According to immunofluorescence, the number of LC3 puncta within NeuN-marked neurons was slightly increased in the SCI group (Fig. S14, A and B). However, treatment with free siRNA^TRAF6^ and PB did not show significant changes in LC3 puncta compared to the SCI group (Fig. S14, A and B). Conversely, treatment with pPB-siRNA^TRAF6^ and NMm-pPB-siRNA^TRAF6^ resulted in further elevation of the number of LC3 puncta, especially in the NMm-pPB-siRNA^TRAF6^ group (Fig. S14, A and B), likely related to the “spatiotemporal delivery” capability of the NMm system. Moreover, pPB-siRNA^TRAF6^ and NMm-pPB-siRNA^TRAF6^ treatments significantly reduced p62 expression in neurons within the region of SCI (Fig. 6A and B), with the lowest fluorescence intensity found after NMm-pPB-siRNA^TRAF6^ treatment (Fig. 6A and B). Consistent with the results of immunofluorescence, further analysis by WB of autophagy-related gene proteins corroborated that NMm-pPB-siRNA^TRAF6^ treatment significantly increased LC3 II and Beclin-1 expression levels (Fig. 6E–H), while reducing p62 expression (Fig. 6E–H). Iba1 labeling also revealed similar outcomes in macrophages/microglia within the spinal cord tissue (Fig. 6C and D, and Fig. S15, A and B). However, PB alone was ineffective in enhancing autophagy in neurons and macrophages after SCI. In contrast, the introduction of siRNA^TRAF6^ (pPB-siRNA^TRAF6^) enhanced the autophagy effect in neurons and macrophages correspondingly. The level of autophagy was further elevated with the encapsulation of NMm by pPB-siRNA^TRAF6^ (NMm-pPB-siRNA^TRAF6^), resulting in the alleviation of impaired autophagic flux. To provide direct visual evidence of autophagic flux modulation, we utilized the mCherry-GFP-LC3 reporter system in PC12 cells under oxidative stress. As shown in Fig. S16, H_2_O_2_ treatment led to a massive accumulation of yellow puncta (GFP+/mCherry+) and a paucity of red puncta compared to the control, indicating a severe blockage of autophagic flux due to lysosomal dysfunction. Treatment with the non-targeting control (NMm-pPB-siRNA^NC^) partially alleviated this phenotype, likely attributed to the intrinsic ROS-scavenging capability of the PB; however, it failed to effectively restore the flux. In contrast, treatment with NMm-pPB-siRNA^TRAF6^ significantly decreased yellow puncta and markedly increased red puncta (autolysosomes) (Fig. S16A). Furthermore, the addition of the lysosomal inhibitor Bafilomycin A1 to the NMm-pPB-siRNA^TRAF6^ treatment group abolished the appearance of red puncta and reverted the phenotype to yellow puncta accumulation. These results confirm that our NMm-pPB-siRNA^TRAF6^ restores the impaired autophagic flux.

### 2.11 NMm-pPB-siRNA^TRAF6^ attenuated pyroptosis by promoting autophagy in neurons after SCI

**Fig. 7:**
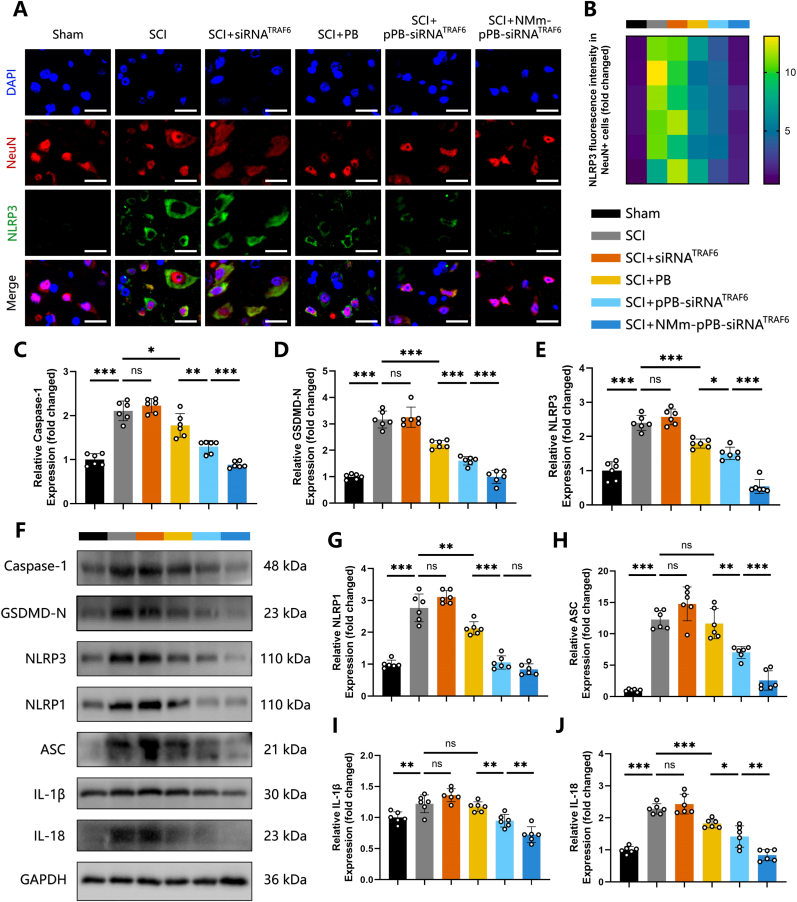
NMm-pPB-siRNA^TRAF6^ attenuated pyroptosis in neurons. (A and B) Double-immunofluorescence staining and quantitative fluorescence intensity analysis of NLRP3 and NeuN in spinal cords of the Sham, SCI, SCI + siRNA^TRAF6^, SCI + PB, SCI + pPB-siRNA^TRAF6^, SCI + NMm-pPB-siRNA^TRAF6^ groups on post-SCI Day 7 (n = 6, mean ± SD). (C to J) Western blot analysis and relative quantification of Caspase-1, GSDMD-N, NLRP3, NLRP1, ASC, IL-1β and IL-18 protein levels in spinal cords of the Sham, SCI, SCI + siRNA^TRAF6^, SCI + PB, SCI + pPB-siRNA^TRAF6^, SCI + NMm-pPB-siRNA^TRAF6^ groups on post-SCI Day 7 (n = 6, mean ± SD). n represents the number of biologically independent samples. P values are shown in graphs with significance levels denoted as ∗P < 0.05, ∗∗P < 0.01, and ∗∗∗P < 0.001. Scale bars, 20 μm (A).

Recent evidence has confirmed the value of pyroptosis, a pro-inflammatory Caspases-dependent programmed cell death, in post-SCI neuroinflammation [62]. Inflammatory stimulation may induce the assembly of NLRP3 inflammasome complex, comprising NLRP3, ASC, and Pro-Caspase-1, etc. Activation of this complex may further promote the cleavage of Pro-Caspase-1 into its active form, Caspase-1, leading to subsequent cleavage of GSDMD, releasing its N-terminal domain to perforate the cell membrane, thus triggering pyroptosis [63]. In mouse and cellular models of Parkinson's disease, PB, as a pyroptosis inhibitor, has been found to alleviate neurodegeneration [23], primarily by protecting microglia and neurons from the damage induced by 1-methyl-4-phenyl-1,2,3,6-tetrahydropyridine. However, we know little about the ability of PB to mitigate neuronal pyroptosis in SCI. In particular, exosomes derived from human umbilical cord mesenchymal stem cells have been found to enable the activation of autophagy and regulation of pyroptosis by silencing TRAF6 expression, thereby alleviating neuropathic pain [33]. In view of the above, the present study further evaluated the effectiveness of the NMm-pPB-siRNA^TRAF6^ nanozyme platform in mitigating neuronal pyroptosis. Based on immunofluorescence staining, neurons in the SCI group exhibited strong fluorescence signals of NLRP3 (Fig. 7A and B) and GSDMD-N (Fig. S17, A and B), indicating an exacerbation in neuronal pyroptosis resulted from SCI (Fig. 7A and B, and Fig. S17, A and B). Compared to the SCI group, PB, pPB-siRNA^TRAF6^, and NMm-pPB-siRNA^TRAF6^ group all revealed reduced expressions of NLRP3 and GSDMD-N (Fig. 7A and B, and Fig. S17, A and B). Consistent with literature reports, PB exhibits superior control over ROS and mitigation of oxidative stress, thus alleviating neuronal pyroptosis to a certain extent. The effectiveness of pPB-siRNA^TRAF6^ may be attributed to the further delivery of siRNA^TRAF6^, while the superior performance of NMm-pPB-siRNA^TRAF6^ is related to the targeted delivery capability of the NMm system to inflamed spinal cord tissue. Further examination of pyroptosis-related proteins in spinal cord tissue using WB revealed similar trends in the expression of Caspase-1, GSDMD-N, NLRP3, NLRP1, ASC, IL-1β, and IL-18 as those of immunofluorescence staining (Fig. 7C–J). These findings validate the effectiveness of NMm-pPB-siRNA^TRAF6^ as a pyroptosis inhibitor in protecting neurons from SCI. In addition, the autophagy inhibitor 3-MA [64] was used to further explore the role of autophagy in the *anti*-pyroptosis effect of NMm-pPB-siRNA^TRAF6^. As a result, the addition of 3-MA weakened the inhibitory effect of NMm-pPB-siRNA^TRAF6^ on post-SCI neuronal pyroptosis (Fig. S18, A to D, and Fig. S19, A to H). To further validate the *anti*-pyroptotic effects in an *in vitro* setting, we employed LPS-stimulated PC12 cells to mimic the neuroinflammatory environment [65]. As shown in Fig. S20, LPS stimulation markedly upregulated the expression of key pyroptosis markers, including NLRP3, Caspase-1, GSDMD-N, IL-1β, and IL-18. Consistent with our *in vivo* results, treatment with NMm-pPB-siRNA^TRAF6^ significantly suppressed the expression of these pyroptosis-related proteins, exhibiting markedly superior efficacy compared to the PB and pPB-siRNA^TRAF6^ groups (Fig. S20, A and B). In short, our NMm-pPB-siRNA^TRAF6^ could mitigate neuronal pyroptosis by promoting autophagy, providing protective effects for SCI.

### 2.12 NMm-pPB-siRNA^TRAF6^ regulated M2 macrophage polarization by promoting autophagy after SCI

**Fig. 8:**
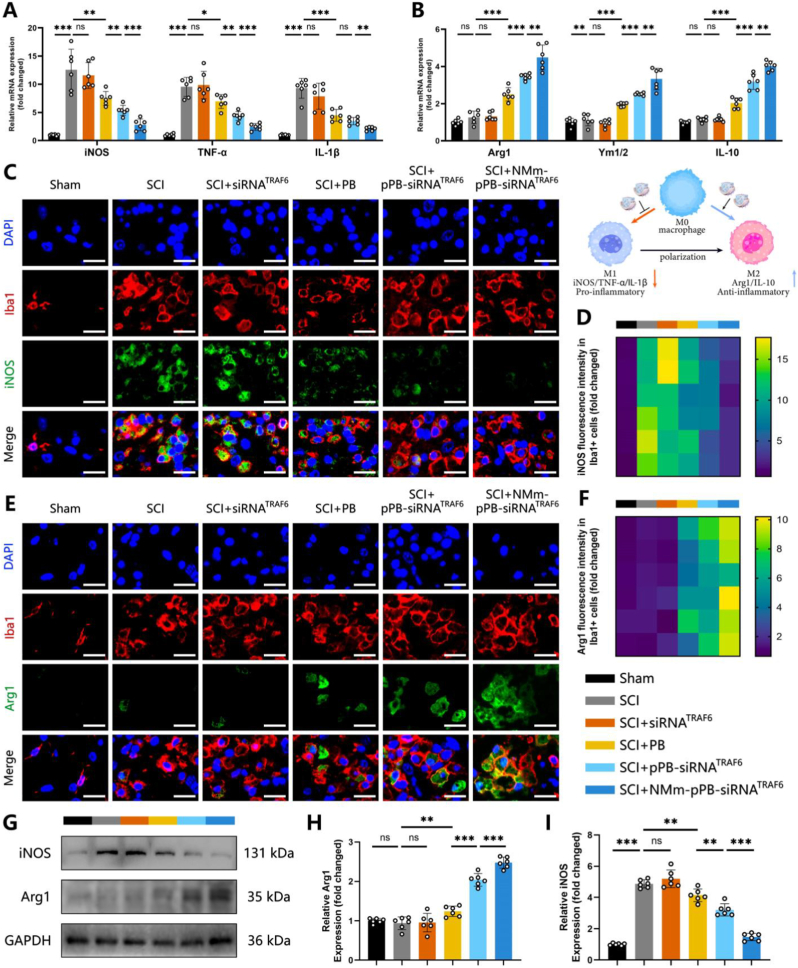
NMm-pPB-siRNA^TRAF6^ regulated M2 macrophage polarization. (A and B) Measurement of the expression levels of M1-related genes (iNOS, TNF-α, and IL-1β) and M2-related genes (Arg1, Ym1/2, and IL-10) in spinal cords on post-SCI Day 7 by qRT-PCR (n = 6, mean ± SD). (C and D) Double-immunofluorescence staining and quantitative fluorescence intensity analysis of iNOS and Iba1 in spinal cords of the Sham, SCI, SCI + siRNA^TRAF6^, SCI + PB, SCI + pPB-siRNA^TRAF6^, SCI + NMm-pPB-siRNA^TRAF6^ groups on post-SCI Day 7 (n = 6, mean ± SD). (E and F) Double-immunofluorescence staining and quantitative fluorescence intensity analysis of Arg1 and Iba1 in spinal cords of the Sham, SCI, SCI + siRNA^TRAF6^, SCI + PB, SCI + pPB-siRNA^TRAF6^, SCI + NMm-pPB-siRNA^TRAF6^ groups on post-SCI Day 7 (n = 6, mean ± SD). (G to I) Western blot analysis and relative quantification of iNOS and Arg1 protein levels in spinal cords of the Sham, SCI, SCI + siRNA^TRAF6^, SCI + PB, SCI + pPB-siRNA^TRAF6^, SCI + NMm-pPB-siRNA^TRAF6^ groups on post-SCI Day 7 (n = 6, mean ± SD). n represents the number of biologically independent samples. P values are shown in graphs with significance levels denoted as ∗P < 0.05, ∗∗P < 0.01, and ∗∗∗P < 0.001. Scale bars, 20 μm (C and E).

Macrophages/microglia can be categorized into pro-inflammatory (M1) and anti-inflammatory (M2) phenotypes. The M1 phenotype may induce secondary injury and promote the progression of SCI, whereas the M2 phenotype can initiate tissue repair [66]. Hence, intervening in macrophage activity by inhibiting M1 macrophage recruitment and promoting M2 macrophage polarization may hold promise for improving post-SCI inflammatory response [67]. Consequently, we sought to determine whether NMm-pPB-siRNA^TRAF6^ treatment could potentially induce a phenotypic shift from M1 to M2 in macrophages/microglia after SCI. qRT-PCR was employed to examine the expression of M1-associated pro-inflammatory markers (i.e., iNOS, TNF-α, and IL-1β) and M2-associated anti-inflammatory markers (i.e., Arg1, Ym1/2, and IL-10). Compared to the SCI group, the PB, pPB-siRNA^TRAF6^, and NMm-pPB-siRNA^TRAF6^ groups showed significantly higher M2 gene expression levels, and obviously lower M1 gene expression levels (Fig. 8A and B). Immunofluorescence results demonstrated decreased iNOS fluorescence signal expression (Fig. 8C and D) and increased Arg1-related fluorescence signal expression (Fig. 8E and F) in the PB, pPB-siRNA^TRAF6^, and NMm-pPB-siRNA^TRAF6^ groups compared to the SCI group. Moreover, pPB-siRNA^TRAF6^ and NMm-pPB-siRNA^TRAF6^, especially NMm-pPB-siRNA^TRAF6^, outperformed PB alone in promoting the M1-to-M2 transition. These findings were further confirmed by WB analysis (Fig. 8G–I). Therefore, PB may facilitate the transition from M1 to M2 by controlling ROS and alleviating oxidative stress. pPB-siRNA^TRAF6^ could further enhance this transition by silencing TRAF6, while NMm-pPB-siRNA^TRAF6^ demonstrated optimal regulatory effects, supported by its “spatiotemporal delivery” capability. Subsequently, 3-MA was utilized for mechanism interpretation to clarify the role of autophagy in this process. Immunofluorescence (Fig. S21, A to D) and WB experimental results (Fig. S22, A to C) both showed that the addition of 3-MA weakened the effect of NMm-pPB-siRNA^TRAF6^ on promoting the polarization of M2 macrophage, as evidenced by an increase in iNOS expression and a decrease in Arg1 expression (Fig. S21, A to D, and Fig. S22, A to C). In addition, we further validated the immunomodulatory effects in BV2 microglial cells. Following LPS stimulation, BV2 cells exhibited a typical M1 pro-inflammatory phenotype, characterized by elevated iNOS expression and reduced Arg1 expression (Fig. S23, A and B). Treatment with NMm-pPB-siRNA^TRAF6^ successfully reversed this trend, significantly downregulating iNOS while upregulating the M2 phenotype marker Arg1. These *in vitro* findings are consistent with our *in vivo* observations. Altogether, NMm-pPB-siRNA^TRAF6^ could regulate M2 macrophage polarization by promoting autophagy to exert a protective effect for SCI ultimately.

### 2.13 NMm-pPB-siRNA^TRAF6^ enhanced long-term motor function recovery and accelerated neuronal regeneration

**Fig. 9:**
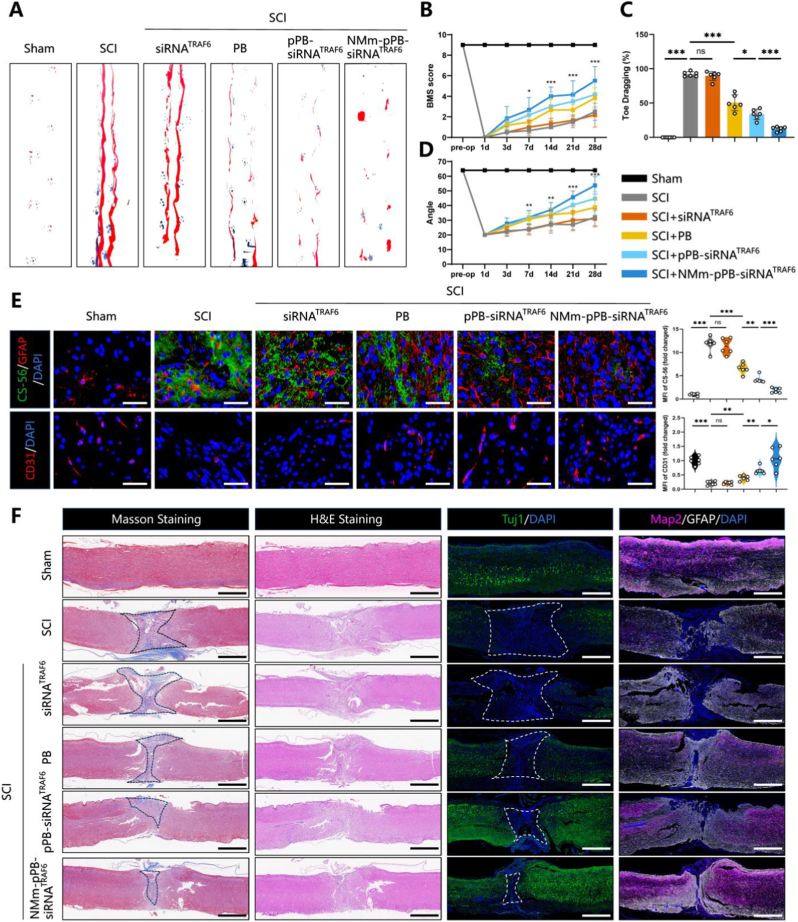
NMm-pPB-siRNA^TRAF6^ enhanced long-term motor function recovery and accelerated neuronal regeneration. (A) Photographs of mouse footprints of the Sham, SCI, SCI + siRNA^TRAF6^, SCI + PB, SCI + pPB-siRNA^TRAF6^, SCI + NMm-pPB-siRNA^TRAF6^ groups at Day 28. Blue: fore paw print; Red: hind paw print. (B and D) BMS results and inclined plane test for the Sham, SCI, SCI + siRNA^TRAF6^, SCI + PB, SCI + pPB-siRNA^TRAF6^, SCI + NMm-pPB-siRNA^TRAF6^ groups on days 0, 1, 3, 7, 14, 21, and 28 (n = 6, mean ± SD). ∗P < 0.05, ∗∗P < 0.01, and ∗P < 0.001 versus the SCI group. (C) Toe dragging (%) analysis of the Sham, SCI, SCI + siRNA^TRAF6^, SCI + PB, SCI + pPB-siRNA^TRAF6^, SCI + NMm-pPB-siRNA^TRAF6^ groups on Day 28 (n = 6, mean ± SD). (E) Immunofluorescence staining and quantitative fluorescence intensity analysis of CS-56 and CD31 in spinal cords of the Sham, SCI, SCI + siRNA^TRAF6^, SCI + PB, SCI + pPB-siRNA^TRAF6^, SCI + NMm-pPB-siRNA^TRAF6^ groups on Day 28 (n = 6, mean ± SD). (F) H&E and Masson staining of the longitudinal spinal cord sections from the Sham, SCI, SCI + siRNA^TRAF6^, SCI + PB, SCI + pPB-siRNA^TRAF6^, SCI + NMm-pPB-siRNA^TRAF6^ groups on Day 28. Immunofluorescence staining and quantitative fluorescence intensity analysis of Tuj1 and Map2 in spinal cords of the Sham, SCI, SCI + siRNA^TRAF6^, SCI + PB, SCI + pPB-siRNA^TRAF6^, SCI + NMm-pPB-siRNA^TRAF6^ groups on Day 28 (n = 6, mean ± SD). n represents the number of biologically independent samples. P values are shown in graphs with significance levels denoted as ∗P < 0.05, ∗∗P < 0.01, and ∗∗∗P < 0.001. Scale bars, 40 μm (E), 625 μm (Black, F), and 600 μm (White, F).

Our subsequent investigation focused on further evaluation of the long-term therapeutic potential of NMm-pPB-siRNA^TRAF6^ in SCI, particularly its effects on promoting neuronal regeneration and enhancing long-term motor function recovery. Accordingly, this study employed a suite of analytical methods including functional scoring, histological examination, immunofluorescence, and qRT-PCR to carry out a comprehensive assessment 28 days post-SCI. Footprint analysis, Basso Mouse Scale (BMS) scoring, and inclined plane tests were performed to gain deeper insights into the role of NMm-pPB-siRNA^TRAF6^ in motor function recovery. Specifically, footprint analysis revealed that compared to the SCI control group, PB, pPB-siRNA^TRAF6^, and NMm-pPB-siRNA^TRAF6^ groups showed significant improvements in hindlimb function at 28 days post-treatment (Fig. 9, A and C). In particular, the NMm-pPB-siRNA^TRAF6^ group displayed the lowest toe drag ratio and most pronounced gait function recovery (Fig. 9, A and C). Subsequently, BMS scores and inclined plane test results at various time points (0 d, 1 d, 3 d, 7 d, 14 d, 21 d, and 28 d) revealed significant improvements in both BMS scores and inclined plane angles in PB, pPB-siRNA^TRAF6^ and NMm-pPB-siRNA^TRAF6^ treated mice (Fig. 9, B and D), especially in the NMm-pPB-siRNA^TRAF6^ group (Fig. 9, B and D). Furthermore, H&E and Masson staining of the injured spinal cord regions indicated that, compared to the Sham group, the SCI group exhibited remarkably increased area of glial scar formation (Fig. 9F), while long-term treatment with PB, pPB-siRNA^TRAF6^, and NMm-pPB-siRNA^TRAF6^ resulted in highly narrowed area of glial scarring (Fig. 9F), particularly in the NMm-pPB-siRNA^TRAF6^ group (Fig. 9F). Given the classification of sensory, motor, or interneurons for neurons in the spinal cord based on function, analysis of these neuronal subtypes may serve as indicators for post-treatment evaluation [68]. According to immunofluorescence, the numbers of Tuj1 positive (neonatal neurons) and Map2 positive (mature neurons) cells were higher in the PB, pPB-siRNA^TRAF6^, and NMm-pPB-siRNA^TRAF6^ groups than those in the SCI group (Fig. 9F). The quantity of Tuj1-positive and Map2-positive cells in the pPB-siRNA^TRAF6^ group exceeded those in the PB group (Fig. 9F), while the number of Tuj1-positive and Map2-positive cells was the highest in the SCI region of NMm-pPB-siRNA^TRAF6^ treated mice (Fig. 9F), with widespread detection throughout the injury area (Fig. 9F). Chondroitin sulfate proteoglycans (CSPG)-positive staining, identified specifically by CS-56 staining for the glycosaminoglycan component of native CSPG, which represents areas of post-SCI scar formation, was significantly lower in the PB, pPB-siRNA^TRAF6^, and NMm-pPB-siRNA^TRAF6^ groups compared to the SCI group (Fig. 9E), with the smallest CSPG-positive staining area found in the NMm-pPB-siRNA^TRAF6^ group especially (Fig. 9E). Therefore, it could be inferred that NMm-pPB-siRNA^TRAF6^ nanozyme platform could effectively reduce scar formation through the regulation of inflammation during the acute injury phase of SCI, minimizing physical barriers to neural growth and angiogenesis. Further investigation of CD31 expression via immunofluorescence revealed enhanced expression in the NMm-pPB-siRNA^TRAF6^ group (Fig. 9E). This enhancement could indicate a significantly enhanced angiogenesis owing to the clearance of ROS and sustained oxygenation. Then, qRT-PCR was further conducted to determine gene expression at the mRNA level within the injured area. The NMm-pPB-siRNA^TRAF6^ group was detected with obviously increased expression of markers for immature neurons (Tuj1, Dcx, and Stmn1), mature neurons (Map2), interneurons (Htr3a, Calb1, and Gad2), sensory neurons (Sox10), motor neurons (Chat and Mnx1), and blood vessels (CD31 and Vcam1) (Fig. S24 and A to L). Therefore, NMm-pPB-siRNA^TRAF6^ can also enhance long-term motor function recovery, in addition to accelerating neuronal regeneration, offering an ideal platform for the treatment of SCI. It is important to acknowledge that autophagy plays a dual role in SCI [69]. While moderate autophagy promotes neuroprotection by clearing damaged organelles and suppressing inflammation [59], excessive or prolonged autophagy can lead to autophagic cell death and hinder tissue recovery [70]. In our study, we specifically targeted the acute to subacute phase of SCI, a window characterized by impaired autophagic flux and lysosomal dysfunction**.** Nevertheless, future studies are needed to further define the optimal intervention period and intensity of autophagy modulation to maximize therapeutic benefits.

### 2.14 Safety profile of NMm-pPB-siRNA^TRAF6^

Finally, our study established short-term (Day 7) and long-term (Day 28) experiments to confirm the biosafety of free siRNA, PB, pPB-siRNA^TRAF6^, and NMm-pPB-siRNA^TRAF6^ treatments. Initially, we analyzed the blood biochemical parameters of each group during the short-term experiment. There was no significant differences in blood biochemical parameters among the different treatment groups (Fig. S25, A to H). Subsequently, in the 28-day long-term assessment, major organs (heart, liver, spleen, lung, and kidney) of mice were examined to identify their biosafety. Histological changes were assessed by H&E staining of the major organs in each group (Fig. S26A), without the observation of evident histopathological changes (Fig. S26A). Collectively, PB, pPB-siRNA^TRAF6^, and NMm-pPB-siRNA^TRAF6^ nanoparticles demonstrated good biosafety in the treatment of SCI.

## 3 Conclusion

Previous application of antioxidants or nanozymes to clear ROS in SCI produces unsatisfactory and incomplete treatment. ROS may be generated continuously by persist dysfunctional cells such as pyroptotic neurons and inflammatory macrophages). Consequently, this study proposes for the first time a strategy that combines detoxifying ROS with modulating cell dysfunction. Autophagy is essential for addressing secondary SCI given its pivotal role in maintaining intracellular homeostasis by eliminating dysfunctional cells. Herein, we introduce an innovative nanozyme platform that utilizes engineered PB nanozymes in conjunction with siRNA^TRAF6^ (pPB-siRNA^TRAF6^), aimed at directly clearing ROS and promoting autophagy. By metaphorically opening an “umbrella” to isolate ROS, this approach can both shield from ROS, and also transition “from rain to sunny” by clearing dysfunctional cells to address the root cause. Moreover, inspired by the sequential recruitment of neutrophils and macrophages to the site of SCI, and further combined with single-cell sequencing analysis, we adopted a hybrid membrane coating strategy. We camouflaged pPB-siRNA^TRAF6^ with Nm and Mm, termed NMm-pPB-siRNA^TRAF6^, achieving “spatiotemporal” delivery for SCI treatment. NMm-pPB-siRNA^TRAF6^ can retain the time-ordered recruitment characteristics of neutrophils-macrophages following SCI, exhibiting “temporal” targeting property; meanwhile, it can also maintain their chemotactic ability towards the injury site, realizing “spatial” targeting delivery. Our experiments demonstrate that NMm-pPB-siRNA^TRAF6^ can alleviate neuronal pyroptosis by promoting neuronal autophagy; it can regulate macrophage polarization by enhancing macrophage autophagy, ultimately improving long-term motor function recovery and accelerating neuronal regeneration. Lastly, our research group develop this nanozyme platform with abilities of clearing ROS and correcting underlying cellular dysfunctions, exhibiting promising clinical potential in treating SCI and other neuroinflammatory diseases. Additionally, the use of hybrid membrane coating technology expands the potential of nanozyme platforms to regulate complex disease microenvironments, providing more comprehensive insights into the targeted delivery of diseases, the preparation, and application of composite biomaterials.

## 4 Methods and materials

### 4.1 Materials

All materials are listed with their sources upon first mention. Primer sequences used for qPCR are detailed in Supplementary Table 1, and all antibodies used in this study are detailed in Supplementary Table 2.

### 4.2 Synthesis of prussian blue nanozymes

Prussian blue nanozymes (PB) were synthesized according to the methods described in previous studies with some modifications [71]. Initially, 116.13 mg of K3 [Fe(CN)6] (Sigma-Aldrich, USA) and 3 g of Polyvinyl pyrrolidone (PVP, Sigma-Aldrich, USA) were dissolved in 40 mL of 0.01M HCl solution (Sigma-Aldrich, USA), followed by stirring at room temperature for 0.5 h to obtain a clear solution. Subsequently, the mixture was transferred to a three-neck flask and heated in an oil bath at 80 °C for 20 h. The product, PB, was then obtained after centrifugation.

### 4.3 Preparation of PEI modified prussian blue nanozymes

Polyethylenimine (PEI, Sigma-Aldrich, USA) modified PB (pPB) are synthesized through electrostatic interactions between cationic PEI and negatively charged PB, resulting in nanoparticles. In brief, a PEI solution (2 ml, 20 mg/ml, pH = 5) is gently added to a PB dispersion (5 ml), stirred for 1 h, and then the product is purified three times using ultrafiltration (100 kDa, MWCO). Subsequently, it is stored at 4 °C.

### 4.4 siRNA loading

To achieve siRNA loading, siRNA solution (20 μM, 5 μl) was added to varying volumes (0, 1, 2, 4, 8, 16, 32 μl) of pPB. The mixture was then gently mixed in a 37 °C water bath for 15 min to allow for the full binding of siRNA molecules with pPB. The binding capability of pPB to siRNA was determined using agarose gel electrophoresis. In brief, after centrifugation, the supernatant was placed in a 2 % agarose gel and electrophoresed in TBE buffer at a constant voltage of 45 V for 30 min. To evaluate the loading capacity, siRNA was stained with GoldView. Cy5-labeled siRNA (siRNA_Cy5_) was used for characterization experiments and *in vivo* imaging assays, while siRNA^TRAF6^ was utilized for functional studies. SiRNA^TRAF6^, siRNA_Cy5_ and siRNA^NC^ were bought from RiboBio, China.

### 4.5 Macrophage membranes extraction

RAW 264.7 cells were selected for the extraction of macrophage membranes. RAW 264.7 cells were acquired from the Cell Bank of the Chinese Academy of Sciences in Shanghai and cultured in DMEM (Gibco, USA) supplemented with 1 % penicillin-streptomycin (Gibco, USA) and 10 % fetal bovine serum (FBS, Gibco, USA) under conditions of 5 % CO_2_ at 37 °C. Macrophage membranes were extracted following a protocol previously established [72]. In brief, collected cells were resuspended in a solution containing 30 mM Tris-HCl (pH 7.4), 0.5 % (w/v) BSA, 75 mM sucrose, 0.5 mM EDTA, and 225 mM mannitol. The suspension was then subjected to ultrasonication using an ultrasonic homogenizer at 30 % power for seven cycles, with a mix of protease and phosphatase inhibitors added. The mixture was centrifuged at 2000 g for 10 min at 4 °C to remove cell nuclei. After repeating the centrifugation three times, membranes were separated by subsequent centrifugation at 21,000*g* for 30 min at 4 °C. The separated membranes were lyophilized, weighed to determine the yield obtained through this process, and stored at −80 °C for further analysis.

### 4.6 Neutrophil-like membranes extraction

HL-60 cells (human promyelocytic leukemia cells) were acquired from the Cell Bank of the Chinese Academy of Sciences in Shanghai and cultured in IMDM medium (Gibco, USA) supplemented with 20 % FBS (Gibco, USA) and 1 % penicillin/streptomycin (Gibco, USA), under conditions of 5 % CO2 at 37 °C. After five days of culture in 1.5 % DMSO (Sigma-Aldrich, USA), neutrophils exhibiting chemotactic properties were considered successfully differentiated. The extraction of neutrophil-like cell membranes was carried out following a previously established protocol. Briefly, after cell collection, they were resuspended in a solution containing 30 mM Tris-HCl pH 7.4, 0.5 % (w/v) BSA, 75 mM sucrose, 0.5 mM EDTA, and 225 mM mannitol, followed by ultrasonication using an ultrasonic homogenizer at 30 % power for seven cycles, with a mix of protease and phosphatase inhibitors added. The mixture was then centrifuged at 2000 g for 10 min at 4 °C to remove cell nuclei. After repeating the centrifugation three times, membranes were separated by subsequent centrifugation at 21,000*g* for 30 min at 4 °C. The separated membranes were lyophilized, weighed to determine the yield obtained through this process, and stored at −80 °C for further analysis.

### 4.7 Membrane coated

Neutrophil-like cell membranes, macrophage membranes, and nanoparticles were uniformly mixed in a solution at a mass ratio of 1:1:2. The mixture was then subjected to ultrasonication for 30 min. Subsequently, the mixture was extruded using a mini extruder (Avanti Polar Lipids, AL) through polycarbonate porous membranes of 1000 nm, 400 nm, and 200 nm sequentially (Whatman, UK). The resulting nanoparticles were centrifuged and washed three times with PBS for further use. To visualize the cell membranes, neutrophil-like cell membranes were labeled with PKH67 (3 μM, Sigma-Aldrich, USA), and macrophage membranes with Dil (3 μM, Thermo Fisher Scientific, USA). Finally, the fluorescence of the neutrophil-like cell membranes and macrophage membranes post-extrusion was observed using confocal laser scanning microscopy (CLSM, Nikon, Japan).

### 4.8 Characterization methods

The morphology of the synthesized nanoparticles was observed using scanning electron microscopy (SEM, Hitachi-S4800 FESEM) and transmission electron microscope (TEM; FEI Talos F200S G2, USA). At room temperature, the hydrodynamic diameter, zeta potential, and polydispersity index (PDI) of the nanoparticles were determined by dynamic light scattering (DLS; Zetasizer Nano ZS ZEN3600, Malvern, UK). The crystalline forms were characterized using an X-ray diffractometer with powder samples (XRD, D8 ADVANCE, Brooker, Germany). Raman spectroscopy was performed using a confocal laser Raman system (inVia, Renishaw, China) and a He−Ne laser (632.8 nm, with an excitation line power of 17 mW). UV–visible–near-infrared (UV–vis–NIR) absorption spectra were obtained with a spectrophotometer (CARY 5000, USA), scanning the range of 200–800 nm. The Fourier-transform infrared (FTIR) spectra of the nanoparticles were measured using an FTIR spectrometer (Tensor II, Bruker, Germany), with a wavenumber range of 400–4000 cm^−1^. Elemental composition and chemical bonding were identified through X-ray photoelectron spectroscopy (XPS, Thermo Fisher ESCALAB 250X).

### 4.9 Hydroxyl radical (·OH) scavenging activity

The ability of PB to scavenge hydroxyl radicals was assessed using a hydroxyl radical scavenging assay kit (Solarbio, China). Hydroxyl radicals were generated through the Fenton reaction by H2O2/Fe^2+^, which oxidized Fe^2+^ to Fe^3+^ in the phenanthroline-Fe^2+^ aqueous solution, leading to a decrease in absorbance at 536 nm. By measuring the inhibition rate of the decrease in absorbance at 536 nm by different concentrations of PB (3.125, 6.25, 12.5, 25, 50, and 100 μg/ml), the capability of PB to scavenge hydroxyl radicals was reflected.

### 4.10 Hydrogen peroxide (H_2_O_2_) scavenging activity

To evaluate the scavenging ability of PB on hydrogen peroxide, solutions of PB at various concentrations (3.125, 6.25, 12.5, 25, 50, and 100 μg/ml) were incubated in 2 ml of PBS buffer containing 200 μM H_2_O_2_ at 37 °C for 24 h. The residual concentration of H_2_O_2_ was determined by measuring the absorbance at 405 nm using a hydrogen peroxide assay kit (Solarbio, China), and the percentage of H_2_O_2_ scavenged was calculated.

### 4.11 Superoxide anion radical (O_2_•-) scavenging activity

To assess the ability of PB to scavenge superoxide anions, in brief, solutions of PB at various concentrations (3.125, 6.25, 12.5, 25, 50, and 100 μg/ml) were incubated with an excess of superoxide anions. The residual superoxide anions were measured using a superoxide anion scavenging assay kit (Solarbio, China). The superoxide anions were generated by the AP-TEMED system and reacted with hydroxylamine hydrochloride to produce NO_2_-. The interaction of NO_2_- with sulfanilamide and α-naphthylamine resulted in the formation of a red azo compound, which exhibits a characteristic absorption peak at 530 nm. The scavenging ability of the samples for superoxide anions is inversely correlated with the absorbance value at 530 nm.

### 4.12 Superoxide dismutase (SOD) assay

Superoxide dismutase (SOD) serves not only as a scavenger of superoxide anions but also as a primary enzyme for the generation of H_2_O_2_, playing a crucial role in biological antioxidant systems. The SOD activity of PB was assessed using an SOD activity assay kit (Sigma, USA). Superoxide anions were generated through a reaction system involving xanthine and xanthine oxidase. O_2_•- reacts with WST-1 to produce a water-soluble yellow formazan, which exhibits an absorption peak at 450 nm. SOD can scavenge O_2_•-, thereby inhibiting the formation of formazan; the deeper the yellow color of the reaction solution, the lower the SOD activity, and vice versa, indicating higher activity. Hence, the production of formazan dye is inversely related to SOD activity. After incubation with various concentrations of PB solutions (3.125, 6.25, 12.5, 25, 50, and 100 μg/ml), the absorbance at 450 nm was measured.

### 4.13 Peroxidase (POD) assay

Peroxidase (POD) refers to a group of enzymes that transform hydrogen peroxide into water during the substrate oxidation process. Guaiacol is selected as the POD substrate to evaluate the POD-like activity at various concentrations of PB (3.125, 6.25, 12.5, 25, 50, and 100 μg/ml). POD can catalyze the oxidation of guaiacol by H_2_O_2_, resulting in the formation of a tea-brown 4-methoxyphenol. The product exhibits a characteristic absorption peak at 470 nm. The activity of peroxidase can be characterized by measuring the change in absorbance values using a UV–vis–NIR spectrophotometer.

### 4.14 Catalase (CAT) assay

According to the manufacturer's instructions, the catalase (CAT) activity was determined using a hydrogen peroxide enzyme activity assay kit (Solarbio, China) across various concentrations of PB (3.125, 6.25, 12.5, 25, 50, and 100 μg/ml). Ammonium molybdate rapidly inhibits the decomposition of H_2_O_2_ by PB, and the residual H_2_O_2_ reacts with ammonium molybdate to form a yellow complex, exhibiting a strong absorption peak at 405 nm. The absorbance is directly proportional to the concentration of hydrogen peroxide. By measuring the amount of residual H_2_O_2_ in the reaction system, the quantity of H_2_O_2_ decomposed by CAT catalysis is determined, thereby reflecting the CAT activity of the PB.

### 4.15 Glutathione peroxidase (GPx) assay

Glutathione peroxidase (GPx) is capable of catalyzing the reduction of reduced glutathione (GSH) to oxidized glutathione (GSSG), thereby reducing toxic hydrogen peroxide to harmless hydroxyl compounds. This process protects the biological membrane from damage by ROS, maintaining normal cellular functions. GPx catalyzes the oxidation of GSH by H_2_O_2_, resulting in the formation of GSSG. The reaction between GSH and 5,5′-dithiobis-(2-nitrobenzoic acid) yields the yellow compound 2-nitro-5-thiobenzoic acid, which exhibits a characteristic absorption peak at 412 nm. By measuring the rate of decrease in absorbance at varying concentrations of PB treatments (3.125, 6.25, 12.5, 25, 50, and 100 μg/ml), the activity of GPx can be characterized.

### 4.16 Single cell RNA sequencing analysis

The single-cell RNA sequencing dataset GSE205037, sourced from the GEO database, incorporates samples from naive and days 3 and 7 post-SCI. Quality control and dimensionality reduction of the single-cell data were conducted using the “Seurat” package, with conditions set to nFeature_RNA >200 & nFeature_RNA <2500 & %Mt < 10. Subsequent steps involved normalization, calculation of mean-variance results, and scaling regression for UMIs and mitochondrial content. Principal component analysis was performed using the “RunPCA” function. The “Harmony” package was utilized to mitigate batch effects and to integrate single-cell data from multiple samples. Clustering was accomplished through the “FindNeighbors” and “FindClusters” functions, with UMAP employed to visualize the clustering outcomes. Dominant cell types were identified using the “SingleR” package and presented in UMAP format, and proportion plots were generated using the “ggplot2″ package.

### 4.17 Cell culture

The preparation of murine bone marrow-derived monocytes (BMDMs) was carried out using a previously described method [66]. Specifically, this method involved the extraction of bone marrow cells from the tibiae and femora of C57BL/6 mice, followed by their culture in DMEM medium (Gibco, USA) containing 10 % L929 cell supernatant and 10 % fetal bovine serum (FBS, Gibco, USA), under conditions of 37 °C and 5 % CO2 for 7 days. To obtain M1-polarized macrophages, BMDMs were cultured with 100 ng/mL of lipopolysaccharide (LPS) and 20 ng/mL of interferon-gamma (IFN-γ); for the generation of M2-polarized macrophages, they were cultured with 20 ng/mL of interleukin-4 (IL-4) and 20 ng/mL of interleukin-13 (IL-13). Human umbilical vein endothelial cells (HUVECs) were obtained from the Shanghai Cell Bank of the Chinese Academy of Sciences and cultured in endothelial cell medium (Sciencell, USA) supplemented with 10 % FBS (Gibco, USA). PC-12 cells (high differentiation) were purchased from Procell Life Science & Technology Company (China) and cultured in RPMI-1640 medium (Sigma-Aldrich, USA) containing 10 % FBS (Gibco, USA).

### 4.18 Target ability analysis

Following the description previously provided [10], the inflammatory targeting of NMm-pPB-siRNA_Cy5_ was tested using transwell chambers (Corning, USA). In the experiment, HUVECs cells were cultured in the upper chamber of the transwell, while BMDMs or PC12 were cultured in the lower chamber. pPB-siRNA_Cy5_ and NMm-pPB-siRNA_Cy5_ were added to the upper chamber. After 24 h, cells on the upper chamber were removed with a cotton swab. Subsequently, BMDMs or PC12 were collected from the lower chamber and analyzed using confocal laser scanning microscopy (CLSM, Nikon, Japan) and flow cytometry (FCM, CytoFLEX, USA).

### 4.19 In vivo imaging assay

In vivo imaging studies were conducted using pPB-siRNA_Cy5_ and NMm-pPB-siRNA_Cy5_. On days 1, 3, and 7 post-SCI, pPB-siRNA_Cy5_ and NMm-pPB-siRNA_Cy5_ were administered intravenously through the tail vein. Imaging of the mice was performed at 2-, 4-, 8-, and 16-h post-injection using the *in vivo* imaging system (IVIS, PerkinElmer, USA), aiming to evaluate the distribution of fluorescent signals within the spinal cord region. Following the final imaging time point, the mice were euthanized by cervical dislocation, and the heart, liver, spleen, lungs, kidneys, and spinal cord were extracted for fluorescent imaging analysis. The analysis of Cy5-related fluorescent signals was conducted using the Living Image software (PerkinElmer, USA). To assess cellular targeting, pPB-siRNA_Cy5_ and NMm-pPB-siRNA_Cy5_ were injected on days 1, 3, and 7 post-SCI. Spinal cords were harvested 16 h later, sectioned, and stained with *anti*-NeuN and *anti*-Iba1 antibodies.

### 4.20 ROS assay

BMDM, PC12, and HUVECs were seeded in 24-well plates and allowed to adhere overnight. Following treatment with the corresponding nanoparticles, the cells were incubated in the dark with a 10 μM solution of 2′,7′-dichlorofluorescin diacetate (DCFH-DA) (Beyotime, China) at 37 °C for 30 min. The treated cells were then washed three times with PBS solution (pH 7.4) to remove residual materials, and finally, cellular observation was conducted using a fluorescence microscope (BZ-X800, Keyence, Japan).

### 4.21 Western blot assay

Proteins were extracted from cells or spinal cord tissues. Total protein was extracted using RIPA lysis buffer (Thermo Fisher Scientific, USA), which contains both protease and phosphatase inhibitors (Sigma-Aldrich, USA). The protein content was then quantified using the BCA Protein Assay Kit (Beyotime, China). Subsequently, the proteins were mixed with 5x SDS-PAGE loading buffer and boiled for 10 min to denature the proteins. These samples were stored at −80 °C. Each sample contained 30 μg of protein, diluted in SDS, separated on a 10 % SDS-PAGE, and then transferred to a PVDF membrane (0.22 μm, Merck Millipore, USA). The PVDF membrane was blocked with 5 % skim milk for 2 h at room temperature, then incubated overnight at 4 °C with the primary antibody. After incubation, the membrane was washed three times with TBST, each time for 5 min, and then incubated with the appropriate HRP-conjugated secondary antibody for an additional 2 h at room temperature. Protein visualization was achieved through chemiluminescence. Protein imaging and quantification were performed using the ChemiDoc XRS+ and Image Lab V3.0 software (Bio-Rad; USA). The primary antibodies used are listed in Table S2.

### 4.22 RNA extraction and qRT-PCR analysis

We purified total RNA (tRNA) from cells and spinal cord tissue using TRIzol reagent (Thermo Fisher Scientific, USA) and quantified RNA content with the Nanodrop 2000 spectrophotometer. Subsequently, cDNA synthesis was performed using the RevertAid First Strand cDNA Synthesis Kit from Takara. Quantitative polymerase chain reaction (qPCR) was carried out using LightCycler® 96 real-time PCR system (Roche) and SYBR Green PCR Kit (Takara). Gene expression data analysis was conducted using the 2-ΔΔCq method. Detailed information about the primers used is provided in Table S1, which were synthesized by Sangon Biotech (China).

### 4.23 Animals

In female C57BL/6 mice, we established an experimental model of spinal cord injury (SCI). Each mouse, matched in age and weight, was randomly assigned to various experimental groups. All animal experiments were conducted in accordance with the Regulations of the People's Republic of China on the Administration of Experimental Animals and were approved by the Animal Care and Use Committee of Wenzhou Medical University (wydw2023-0363). Based on established methods [60], a mouse SCI model was constructed. Initially, anesthesia was administered through intraperitoneal injection using 1 % pentobarbital sodium. Following anesthesia, a standard laminectomy at the T9-T10 vertebrae was performed to expose the dura mater of the spinal cord. Subsequently, moderate SCI was induced using a spinal cord impactor device, which involved dropping a 10 g weight bar, 3.0 mm in diameter, from a 20 mm height directly onto the surface of the exposed spinal cord. This procedure was conducted in accordance with the recommendations of the equipment manufacturer (W.M. KECK+, Model III, USA). Muscle and skin were subsequently sutured with 4–0 silk sutures using a layered suturing technique. Post-SCI, manual bladder expression was performed twice daily until the bladder reflex recovered. Except for *in vivo* imaging analysis (n = 4, where n represents the number of biological replicates), the group size for all other animal experiments was n = 6. For *in vivo* imaging analysis, mice were randomly divided into three groups: Control, pPB-siRNA_Cy5_, and NMm-pPB-siRNA_Cy5_ groups, with specific experimental methods detailed in the “In Vivo Imaging Analysis” section. In short-term experiments, mice were randomly divided into six groups: sham operation (anesthesia and laminectomy only, without causing SCI); SCI (SCI induced, with PBS injected intravenously as a control); SCI + siRNA^TRAF6^ (SCI induced, with siRNA^TRAF6^ injected intravenously, 2.5 nmol); SCI + PB (SCI induced, with PB injected intravenously, 40 mg/kg); SCI + pPB-siRNA^TRAF6^ (SCI induced, with pPB-siRNA^TRAF6^ injected intravenously, where PB was 40 mg/kg, siRNA was 2.5 nmol); SCI + NMm-pPB-siRNA^TRAF6^ (SCI induced, with NMm-pPB-siRNA^TRAF6^ injected intravenously, where PB was 40 mg/kg, siRNA was 2.5 nmol). A total of four injections were administered on days 1, 3, 5, and 7 post-SCI. To explore the effect of autophagy regulation by the NMm-pPB-siRNA^TRAF6^ group on SCI repair, mice were randomly divided into three groups: SCI (SCI induced, with PBS injected intravenously as a control, and physiological saline injected intraperitoneally as a 3-MA control); SCI + NMm-pPB-siRNA^TRAF6^ (SCI induced, with NMm-pPB-siRNA^TRAF6^ injected intravenously, where PB was 40 mg/kg, siRNA was 2.5 nmol, and physiological saline injected intraperitoneally as a 3-MA control); SCI + NMm-pPB-siRNA^TRAF6^+3-MA (SCI induced, with NMm-pPB-siRNA^TRAF6^ injected intravenously, where PB was 40 mg/kg, siRNA was 2.5 nmol, and 3-MA, 15 mg/kg, dissolved in physiological saline, injected intraperitoneally). A total of four injections were administered on days 1, 3, 5, and 7 post-SCI. Half an hour before the tail vein injection of nanoparticles, 3-MA was administered via intraperitoneal injection. In long-term experiments, mice were divided into six groups, identical to the short-term experimental grouping. A total of ten injections were administered, with injection points on days 1, 3, 5, and 7 post-SCI during the first week, followed by two injections per week until the fourth week (28 days). At the end of the experiment, mice were euthanized with an overdose of pentobarbital sodium, and tissue samples were collected on day 7 (short-term experiments) or 28 (long-term experiments) post-SCI for histological examination.

### 4.24 Evaluation of functional behavior

After SCI, Basso Mouse Scale (BMS) and inclined plane tests were conducted on Day 0, 1, 3, 7, 14, 21, and 28. The BMS score ranges from 0 to 9, where 0 indicates complete paralysis and 9 represents full motor function. The inclined plane test was utilized to determine the maximum angle at which mice could maintain balance on the testing apparatus for at least 5 s without falling. Both experiments were conducted in an open field. Gait analysis was performed at Day 28, with the mice's forelimbs and hindlimbs dyed blue and red, respectively, and toe drag rates measured using footprint analysis.

### 4.25 DHE assay

DHE tissue reactive oxygen species assay kit was utilized to determine the levels of ROS in spinal cord tissue followed the manufacturer's instructions. The specific steps are as follows: Firstly, fresh spinal cord tissue samples were taken and washed with PBS. Then, 50 mg of spinal cord tissue was weighed and added to 1 ml of homogenization buffer A, thoroughly homogenized using a glass homogenizer. Subsequently, centrifugation was conducted at 100×*g* for 3 min at 4 °C, the pellet was discarded, and the supernatant was retained. 200 μl of the homogenate supernatant was mixed with 2 μl of DHE probe, then placed in a black 96-well plate and incubated at 37 °C in the dark for 30 min. Finally, fluorescence intensity was detected using the IVIS system (PerkinElmer, USA) at excitation wavelengths of 488–535 nm and an emission wavelength of 610 nm. Analysis of the fluorescence signal was performed using Living Image software (PerkinElmer, USA).

### 4.26 Immunofluorescence assay

Spinal cord specimens were collected and dissected for immunofluorescence staining. The sections were dewaxed, dehydrated in a gradient, and then treated with 10.2 mM sodium citrate buffer at 95 °C for 20 min. Subsequently, the sections were permeabilized with 0.1 % (v/v) PBS-Triton X-100 for 10 min. Next, the sections were blocked with PBS containing 10 % (v/v) bovine serum albumin (BSA) for 1 h. Then, they were incubated overnight with primary antibodies at 4 °C (all primary antibodies used in immunofluorescence are detailed in Table S2). Following this, the sections were washed three times with PBS for 5 min each at room temperature and incubated with the corresponding labeled secondary antibodies for 1 h at room temperature. Finally, the images were captured and evaluated using a confocal microscope (Nikon, Japan). The fluorescence intensity of each neuron or macrophage for the respective markers was estimated using ImageJ software. The IF images of LC3 puncta in each neuron or macrophage were determined manually in a double-blind manner.

### 4.27 H&E staining and masson staining

Mice were anesthetized with 2 % (w/v) sodium pentobarbital and perfused with PBS saline to prepare spinal cord tissues. Subsequently, the spinal cord tissues containing the injury (centered around the injury site) were dissected and fixed in 4 % (w/v) paraformaldehyde for 48 h. The samples were then embedded in paraffin, and corresponding longitudinal sections were prepared. The prepared tissue sections were dewaxed and rehydrated, followed by staining with H&E and Masson staining solutions (Servicebio, China) according to the manufacturer's instructions. Finally, these sections were imaged using a digital pathology slide scanner (KF-PRO-005, 376 KFBIO, China).

### 4.28 Statistical analysis

Statistical comparisons between two independent groups were conducted using an unpaired two-tailed *t*-test. For multiple comparisons, a one-way analysis of variance (ANOVA) with a post-hoc Tukey test was utilized. Each ‘n' represents the number of biologically independent samples. Unless otherwise specified, statistical analyses and tests were carried out using GraphPad Prism v.9.0. P-values are provided in the figures, with significance levels denoted as ∗P < 0.05, ∗∗P < 0.01, and ∗∗∗P < 0.001.

## CRediT authorship contribution statement

**Hongyi Jiang:** Writing – original draft, Visualization, Validation, Project administration, Methodology, Investigation, Data curation, Conceptualization. **Liting Jiang:** Project administration, Methodology, Formal analysis, Data curation. **Tian Xia:** Resources, Project administration, Methodology. **Jiachen Yu:** Visualization, Software. **Yitian Bu:** Visualization, Validation, Software, Methodology. **Hanting Shen:** Investigation, Formal analysis, Data curation. **Liang Zhu:** Project administration, Methodology. **Chihao Lin:** Software, Data curation. **Yumeng Wang:** Methodology, Investigation, Formal analysis. **Yituo Chen:** Methodology. **Rongjie Liu:** Software. **Junfeng Shi:** Supervision. **Jilong Wang:** Visualization, Supervision, Funding acquisition. **Junjie Deng:** Writing – review & editing, Validation, Project administration, Funding acquisition. **Haixiao Liu:** Visualization, Validation, Software, Funding acquisition. **Xiaoyun Pan:** Writing – review & editing, Supervision, Funding acquisition, Data curation, Conceptualization.

## Ethical approval

The animal study was reviewed and approved by Wenzhou Medical University Animal Care and Use Committee (wydw2023-0363).

## Declaration of competing interest

The authors declare that they have no known competing financial interests or personal relationships that could have appeared to influence the work reported in this paper.

## Data Availability

Data will be made available on request.

## References

[1] Global regional (2019). National burden of neurological disorders, 1990-2016: a systematic analysis for the global burden of disease study 2016. Lancet Neurol..

[2] Fehlings M.G. (2017). A clinical practice guideline for the management of acute spinal cord injury: introduction, rationale, and scope. Glob. Spine J..

[3] Crispo J.A.G., Kuramoto L.K., Cragg J.J. (2023). Global burden of spinal cord injury: future directions. Lancet Neurol..

[4] Ahuja C.S. (2017). Traumatic spinal cord injury. Nat. Rev. Dis. Primers.

[5] Katoh H., Yokota K., Fehlings M.G. (2019). Regeneration of spinal cord connectivity through stem cell transplantation and biomaterial scaffolds. Front. Cell. Neurosci..

[6] Orr M.B., Gensel J.C. (2018). Spinal cord injury scarring and inflammation: therapies targeting glial and inflammatory responses. Neurotherapeutics : the journal of the American Society for Experimental NeuroTherapeutics.

[7] Hellenbrand D.J. (2021). Inflammation after spinal cord injury: a review of the critical timeline of signaling cues and cellular infiltration. J. Neuroinflammation.

[8] Xu T. (2023). Git1-PGK1 interaction achieves self-protection against spinal cord ischemia-reperfusion injury by modulating Keap1/Nrf2 signaling. Redox Biol..

[9] Zheng J. (2024). Engineered multifunctional zinc-organic framework-based aggregation-induced emission nanozyme for accelerating spinal cord injury recovery. ACS Nano.

[10] Xiong T. (2023). Multifunctional integrated nanozymes facilitate spinal cord regeneration by remodeling the extrinsic neural environment. Advanced science (Weinheim, Baden-Wurttemberg, Germany).

[11] You Y. (2024). In situ piezoelectric-catalytic anti-inflammation promotes the rehabilitation of acute spinal cord injury in synergy. Adv. Mater..

[12] Sterner R.C., Sterner R.M. (2022). Immune response following traumatic spinal cord injury: pathophysiology and therapies. Front. Immunol..

[13] Yan S., Zhang L., Wang S., Wu T., Gong Z. (2018). Inhibition of the ras/raf/extracellular signal-regulated kinase 1/2 signaling pathway by compounds of natural origin for possible treatment of spinal cord injury: an in silico approach. Exp. Ther. Med..

[14] Ji Z.S. (2022). Highly bioactive iridium metal-complex alleviates spinal cord injury via ROS scavenging and inflammation reduction. Biomaterials.

[15] Yu D. (2022). Hydrogen-bonded organic framework (HOF)-based single-neural stem cell encapsulation and transplantation to remodel impaired neural networks. Angew. Chem..

[16] Salvemini D., Riley D.P., Cuzzocrea S. (2002). SOD mimetics are coming of age. Nat. Rev. Drug Discov..

[17] Hart P.C. (2015). MnSOD upregulation sustains the warburg effect via mitochondrial ROS and AMPK-Dependent signalling in cancer. Nat. Commun..

[18] Shang L. (2023). Ultrasound-augmented multienzyme-like nanozyme hydrogel spray for promoting diabetic wound healing. ACS Nano.

[19] Guo Y. (2024). Multifunctional PtCuTe nanosheets with strong ROS scavenging and ROS-independent antibacterial properties promote diabetic wound healing. Adv. Mater..

[20] Yu P. (2023). Mimicking antioxidases and hyaluronan synthase: a zwitterionic nanozyme for photothermal therapy of osteoarthritis. Adv. Mater..

[21] Wang W. (2023). Trimanganese tetroxide nanozyme protects cartilage against degeneration by reducing oxidative stress in osteoarthritis. Advanced science (Weinheim, Baden-Wurttemberg, Germany).

[22] Nukolova N.V. (2018). Multilayer polyion complex nanoformulations of superoxide dismutase 1 for acute spinal cord injury. J. Contr. Release : official journal of the Controlled Release Society.

[23] Ma X. (2022). Prussian blue nanozyme as a pyroptosis inhibitor alleviates neurodegeneration. Adv. Mater..

[24] Liu Y. (2020). Integrated Cascade nanozyme catalyzes in vivo ROS scavenging for anti-inflammatory therapy. Sci. Adv..

[25] Li B. (2023). Single-atom nanocatalytic therapy for suppression of neuroinflammation by inducing autophagy of abnormal mitochondria. ACS Nano.

[26] Mizushima N., Komatsu M. (2011). Autophagy: renovation of cells and tissues. Cell.

[27] Kim K.H., Lee M.S. (2014). Autophagy--a key player in cellular and body metabolism. Nat. Rev. Endocrinol..

[28] Li Y. (2022). Impairment of autophagy after spinal cord injury potentiates neuroinflammation and motor function deficit in mice. Theranostics.

[29] Zhou K. (2020). TFE3, a potential therapeutic target for spinal cord injury via augmenting autophagy flux and alleviating ER stress. Theranostics.

[30] Dou Y., Tian X., Zhang J., Wang Z., Chen G. (2018). Roles of TRAF6 in central nervous system. Curr. Neuropharmacol..

[31] Dou Y. (2017). Tumor necrosis factor receptor-associated factor 6 participates in early brain injury after subarachnoid hemorrhage in rats through inhibiting autophagy and promoting oxidative stress. J. Neurochem..

[32] Chen K.H. (2020). Melatonin against acute ischaemic stroke dependently via suppressing both inflammatory and oxidative stress downstream signallings. J. Cell Mol. Med..

[33] Hua T. (2022). Huc-MSCs-derived exosomes attenuate inflammatory pain by regulating microglia pyroptosis and autophagy via the miR-146a-5p/TRAF6 axis. J. Nanobiotechnol..

[34] Yang Y. (2023). MiR-124 reduced neuroinflammation after traumatic brain injury by inhibiting TRAF6. Neuroimmunomodulation.

[35] Wang H. (2022). PEGylated Prussian blue nanoparticles for modulating polyethyleneimine cytotoxicity and attenuating tumor hypoxia for dual-enhanced photodynamic therapy. J. Mater. Chem. B.

[36] Liu Z. (2020). Advanced oxidation protein products induce microglia-mediated neuroinflammation via MAPKs-NF-κB signaling pathway and pyroptosis after secondary spinal cord injury. J. Neuroinflammation.

[37] Van Broeckhoven J., Sommer D., Dooley D., Hendrix S., Franssen A. (2021). Macrophage phagocytosis after spinal cord injury: when friends become foes. Brain : J. Neurol..

[38] Hou R. (2022). Prussian blue nanozyme promotes the survival rate of skin flaps by maintaining a normal microenvironment. ACS Nano.

[39] Feng L. (2021). Neutrophil-like cell-membrane-coated nanozyme therapy for ischemic brain damage and long-term neurological functional recovery. ACS Nano.

[40] Ye C. (2022). Prussian blue nanozyme normalizes microenvironment to delay osteoporosis. Adv. Healthcare Mater..

[41] Lee J.Y. (2016). Jmjd3 mediates blood-spinal cord barrier disruption after spinal cord injury by regulating MMP-3 and MMP-9 expressions. Neurobiol. Dis..

[42] Zuo Y. (2023). Controlled delivery of a neurotransmitter-agonist conjugate for functional recovery after severe spinal cord injury. Nat. Nanotechnol..

[43] Neirinckx V. (2014). Neutrophil contribution to spinal cord injury and repair. J. Neuroinflammation.

[44] Fleming J.C. (2006). The cellular inflammatory response in human spinal cords after injury. Brain : J. Neurol..

[45] Salvador A.F.M. (2023). Age-dependent immune and lymphatic responses after spinal cord injury. Neuron.

[46] Dehaini D. (2017). Erythrocyte-platelet hybrid membrane coating for enhanced nanoparticle functionalization. Advanced materials (Deerfield Beach, Fla.

[47] Zhuang J. (2020). Multimodal enzyme delivery and therapy enabled by cell membrane-coated metal-organic framework nanoparticles. Nano Lett..

[48] He H. (2018). Leutusome: a biomimetic nanoplatform integrating plasma membrane components of leukocytes and tumor cells for remarkably enhanced solid tumor homing. Nano Lett..

[49] Liu W.L. (2019). Expandable immunotherapeutic nanoplatforms engineered from cytomembranes of hybrid cells derived from cancer and dendritic cells. Adv. Mater..

[50] Kolaczkowska E., Kubes P. (2013). Neutrophil recruitment and function in health and inflammation. Nat. Rev. Immunol..

[51] Hou J. (2019). Accessing neuroinflammation sites: monocyte/Neutrophil-mediated drug delivery for cerebral ischemia. Sci. Adv..

[52] Tang Z. (2023). Neutrophil-mimetic, ROS responsive, and oxygen generating nanovesicles for targeted interventions of refractory rheumatoid arthritis. Small.

[53] Yin Y. (2022). Biomimetic neutrophil and macrophage dual membrane-coated nanoplatform with orchestrated tumor-microenvironment responsive capability promotes therapeutic efficacy against glioma. Chem. Eng. J..

[54] Liu Y. (2023). Neutrophil-membrane-coated biomineralized metal-organic framework nanoparticles for atherosclerosis treatment by targeting gene silencing. ACS Nano.

[55] Zrzavy T. (2021). Acute and non-resolving inflammation associate with oxidative injury after human spinal cord injury. Brain : J. Neurol..

[56] Munteanu C. (2022). Main cations and cellular biology of traumatic spinal cord injury. Cells.

[57] Yao C., Cao X., Yu B. (2021). Revascularization after traumatic spinal cord injury. Front. Physiol..

[58] Xu L. (2023). Nanozyme-integrated thermoresponsive in situ forming hydrogel enhances mesenchymal stem cell viability and paracrine effect for efficient spinal cord repair. ACS Appl. Mater. Interfaces.

[59] Levine B., Kroemer G. (2019). Biological functions of autophagy genes: a disease perspective. Cell.

[60] Chen Y. (2024). DADLE promotes motor function recovery by inhibiting cytosolic phospholipase A(2) mediated lysosomal membrane permeabilization after spinal cord injury. Br. J. Pharmacol..

[61] Zhang H. (2023). Elamipretide alleviates pyroptosis in traumatically injured spinal cord by inhibiting cPLA2-induced lysosomal membrane permeabilization. J. Neuroinflammation.

[62] Al Mamun A. (2021). Role of pyroptosis in spinal cord injury and its therapeutic implications. J. Adv. Res..

[63] Yu P. (2021). Pyroptosis: mechanisms and diseases. Signal Transduct. Targeted Ther..

[64] Zhang H. (2023). 3,4-Dimethoxychalcone, a caloric restriction mimetic, enhances TFEB-Mediated autophagy and alleviates pyroptosis and necroptosis after spinal cord injury. Theranostics.

[65] Wang Y., Liu X., Wang Q., Yang X. (2020). Roles of the pyroptosis signaling pathway in a sepsis-associated encephalopathy cell model. J. Int. Med. Res..

[66] Wang Y. (2023). Human adipose tissue lysate-based hydrogel for lasting immunomodulation to effectively improve spinal cord injury repair. Small.

[67] Rong Y. (2023). Engineered extracellular vesicles for delivery of siRNA promoting targeted repair of traumatic spinal cord injury. Bioact. Mater..

[68] Sharpee T.O. (2014). Toward functional classification of neuronal types. Neuron.

[69] Plaza-Zabala A., Sierra-Torre V., Sierra A. (2017). Autophagy and microglia: novel partners in neurodegeneration and aging. Int. J. Mol. Sci..

[70] Liu Y., Levine B. (2015). Autosis and autophagic cell death: the dark side of autophagy. Cell Death Differ..

[71] Bai H. (2021). Zwitterion-functionalized hollow mesoporous Prussian blue nanoparticles for targeted and synergetic chemo-photothermal treatment of acute myeloid leukemia. J. Mater. Chem. B.

[72] Xu W. (2021). Cancer cell membrane-coated nanogels as a redox/pH dual-responsive drug carrier for tumor-targeted therapy. J. Mater. Chem. B.

