# Supplementary material for: Spatiotemporal delivery of multifunctional nanozymes for neuroinflammation alleviation via autophagy modulation in spinal cord injury
Source: Mater Today Bio. 2025 Dec 23;36:102734. doi: 10.1016/j.mtbio.2025.102734 (PMC12813177; doi:10.1016/j.mtbio.2025.102734)
Supplement: Multimedia component 1 [file mmc1.docx]

Supplementary Information

Spatiotemporal Delivery of Multifunctional Nanozymes for Neuroinflammation Alleviation via Autophagy Modulation in Spinal Cord Injury

Hongyi Jiang^1,2,3,+^, Liting Jiang^1,2,3,+^, Tian Xia^4,+^, Jiachen Yu^1,2,3^, Yitian Bu^1,2,3^, Hanting Shen^1,2,3^, Liang Zhu^1,2,3^, Chihao Lin^1,2,3^, Yumeng Wang^4^, Yituo Chen^1,2,3^, Rongjie Liu^1,2,3^, Junfeng Shi^5^, Jilong Wang^1,2,3,4,*^, Junjie Deng^1,2,3,4,*^, Haixiao Liu^1,2,3,*^, Xiaoyun Pan^1,2,3,*^

1 Department of Orthopedics, The Second Affiliated Hospital and Yuying Children’s Hospital of Wenzhou Medical University, Wenzhou, Zhejiang Province, China

2 Key Laboratory of Orthopedics of Zhejiang Province, Wenzhou, Zhejiang Province, China

3 The Second Clinical School of Medicine, Wenzhou Medical University, Wenzhou, Zhejiang Province, China

4 Wenzhou Institute, University of Chinese Academy of Sciences, Wenzhou, Zhejiang, People's Republic of China.

5 The Affiliated XiangTan Central Hospital of Hunan University, School of Biomedical Sciences, Hunan University.

* Corresponding authors:

Jilong Wang, E-mail: wangjilong@ucas.ac.cn

Junjie Deng, E-mail: j.deng@ucas.ac.cn

Haixiao Liu, E-mail: spineliu@163.com

Xiaoyun Pan, E-mail: xiaoyunpan@wmu.edu.cn

Address: The Second Affiliated Hospital and Yuying Children’s Hospital of Wenzhou Medical University Wenzhou, Zhejiang, China.

+ These authors contributed equally to this work and share first authorship.

**The PDF file includes:**

Supplementary methods

Supplementary Fig. 1 to Supplementary Fig. 26

Supplementary Table 1 to Supplementary Table 2

**Supplementary methods**

**Cell membrane proteins characterization**

The protein content of cell membranes was measured using a BCA Protein Assay Kit (Beyotime, China). The expression of neutrophil membrane proteins (CXCL1, CXCL2, LFA-1) in differentiated and undifferentiated HL-60 cells was detected by Western blot, with the detailed experimental method referred to in the Western blot assay section, and the antibodies used are listed in the Table. S2. The specificity markers for macrophages (Integrin α4, Integrin β1, Integrin αVβ3, CCR2) were confirmed through Western blot and immunofluorescence. The detailed method for the Western blot experiment is referred to in the Western blot assay section, and the antibodies used are listed in the Table. S2. Immunofluorescence was performed according to previously established methods, with RAW 264.7 cells fixed in 4% PFA for 15 minutes, followed by permeabilization with 0.5% Triton X-100 (Sigma-Aldrich, USA) for 10 minutes. After blocking with 10% goat serum (Meilunbio, China) at 37°C for 1 h, primary antibodies including Integrin α4, Integrin β1, Integrin αVβ3, and CCR2 (see Table. S2 for details) were added and incubated overnight at 4°C. Subsequently, sections were stained with Alexa Fluor 488 and Alexa Fluor 594 conjugated secondary antibodies (Abcam, UK) at 37°C for 2 hours. Finally, cells were observed under a fluorescence microscope (BZX800, Keyence, Japan).

**ABTS scavenging activity**

The ABTS method can be used for the determination of the antioxidant capacity of both hydrophilic and lipophilic substances, making it one of the most widely used indirect detection methods. ABTS, or 2,2'-azino-bis(3-ethylbenzothiazoline-6-sulfonic acid), reacts with potassium persulfate to generate stable blue-green cationic ABTS radicals, exhibiting a maximum absorption peak at 734 nm. The antioxidant components contained could react with ABTS radicals, causing the reaction system to decolorize, and the absorbance at 734 nm decreases. Within a certain range, the change in absorbance is directly proportional to the degree of free radical scavenging. The steps for ABTS radical scavenging activity are as follows: solutions of PB at different concentrations (6.25, 12.5, 25, 50, and 100 μg/ml) are added to the ABTS radical solution and reacted in the dark for 30 minutes. Then, the ultraviolet absorption peak is measured using a UV-Vis-NIR spectrophotometer to calculate the ABTS clearance rate.

**DPPH scavenging activity**

1,1-Diphenyl-2-picrylhydrazyl (DPPH) radical is a very stable nitrogen-centered radical, serving as one of the important indicators of sample antioxidant capacity. The DPPH radical, with its single electron, presents a purple solution that exhibits strong absorption at 517 nm. When antioxidants are present, the DPPH radicals are scavenged, leading to a lighter color of the solution, and the absorbance at 517 nm decreases. Within a certain range, the change in absorbance is directly proportional to the degree of free radical scavenging. The steps for DPPH radical scavenging activity are as follows: solutions of PB at different concentrations (6.25, 12.5, 25, 50, and 100 μg/ml) are added to the DPPH solution and reacted in the dark for 30 minutes. Then, the solution's ultraviolet absorption is measured using a UV-Vis-NIR spectrophotometer to quantitatively calculate the DPPH clearance rate of different concentration solutions.

**Protein docking and visualization**

Utilizing the UniProt database (<https://www.uniprot.org/>), download the three-dimensional structural data of the target proteins in mice. Pair the proteins for docking in accordance with the specified requirements using ZDOCK 3.0.2, and upon completion, select the optimal docking result. Employ PyMol V2.4.0 software to annotate and display the binding sites of the docking complexes.

**Cell viability**

Cell Counting Kit-8 assay (CCK-8, Meilunbio, China) was employed following standard procedures to investigate the effects of various treatments on cell viability. The formula for calculating cell viability is as follows: Cell Viability (%) = (As - Ab) / (Ac - Ab) × 100%, where As represents the absorbance of the experimental wells (containing cells, culture medium, CCK-8 solution, and drug solution), Ac represents the absorbance of the control wells (containing cells, culture medium, and CCK-8 solution without the drug), and Ab represents the absorbance of the blank wells (containing only the culture medium and CCK-8 solution, without cells and drugs). The experiment was conducted in a 96-well plate, with each well seeded with 4 × 10^3^ cells. Subsequently, 100 µL of cell culture medium (containing 10 µL of CCK-8 solution) was added to each well at designated time points, followed by incubation at 37°C for 2 hours. Finally, the absorbance of the solutions in each well was measured at a wavelength of 450 nm using a microplate reader.

**Transwell migration assay**

In the Transwell migration assay, a 24-well Transwell plate (Corning, USA) was utilized. Approximately 3×10^4^ HUVECs were seeded into the upper chamber. Subsequently, each group received appropriate treatments. After 18 hours, the cells were fixed with 4% paraformaldehyde for 15 minutes. Following this, the cells were stained with crystal violet (Beyotime, China) for an additional 15 minutes. Images of the migrated cells were captured using an optical microscope (SZ61TR, Olympus, Japan), and cell number counting was conducted using Image J.

**Tube formation assay**

Matrigel matrix (Corning, USA) was utilized for the tubular structure formation experiment. Each well of the 48-well plate was filled with 150 μl of Matrigel matrix and appropriately mixed on ice to prevent premature solidification of the Matrigel. Subsequently, the plate was incubated at 37℃ for 1 hour to allow the Matrigel matrix to gel. Approximately 6×10^4^ HUVECs were then seeded into each well of the 48-well plate. After 8 hours, the tubular structures formed by the HUVECs were observed through an optical microscope (SZ61TR, Olympus, Japan).

**GFP-mCherry-LC3 assay**

Ad-mCherry-GFP-LC3 is an adenoviral vector that expresses the mCherry-GFP-LC3B fusion protein and can be used to monitor autophagic flux after infecting cells. Cells were seeded into 6-well plates and infected with the virus diluted in fresh medium at a multiplicity of infection (MOI) of 30. The cells were subsequently examined using a Nikon confocal microscope (Tokyo, Japan). Because GFP is rapidly quenched in the acidic environment of autolysosomes whereas mCherry remains stable, autophagosomes appear as yellow puncta (overlap of green and red), while autolysosomes appear as red-only puncta. An increase in autophagic flux is characterized by an elevated number of red puncta relative to yellow puncta, whereas impaired flux leads to an accumulation of yellow puncta due to blocked autophagosome–lysosome fusion or defective lysosomal degradation.

**In vivo toxicity evaluation**

In the short-term experiment (7 days post-SCI), mouse serum samples were obtained using standard procedures to assess bio-safety in vivo by evaluating blood biochemistry related indicators. In the long-term experiment (28 days post-SCI), mice were euthanized, and major organs (heart, liver, kidneys, lungs, and spleen) were collected for H&E staining to evaluate systemic pathological changes.

**Statistical analysis**

Statistical comparisons between two independent groups were conducted using an unpaired two-tailed t-test. For multiple comparisons, a one-way analysis of variance (ANOVA) with a post-hoc Tukey test was utilized. Each 'n' represents the number of biologically independent samples. Unless otherwise specified, statistical analyses and tests were carried out using GraphPad Prism v.9.0. P-values are provided in the figures, with significance levels denoted as *P<0.05, **P<0.01, and ***P<0.001.

**Supplementary Fig. 1 to Supplementary Fig. 26**

**
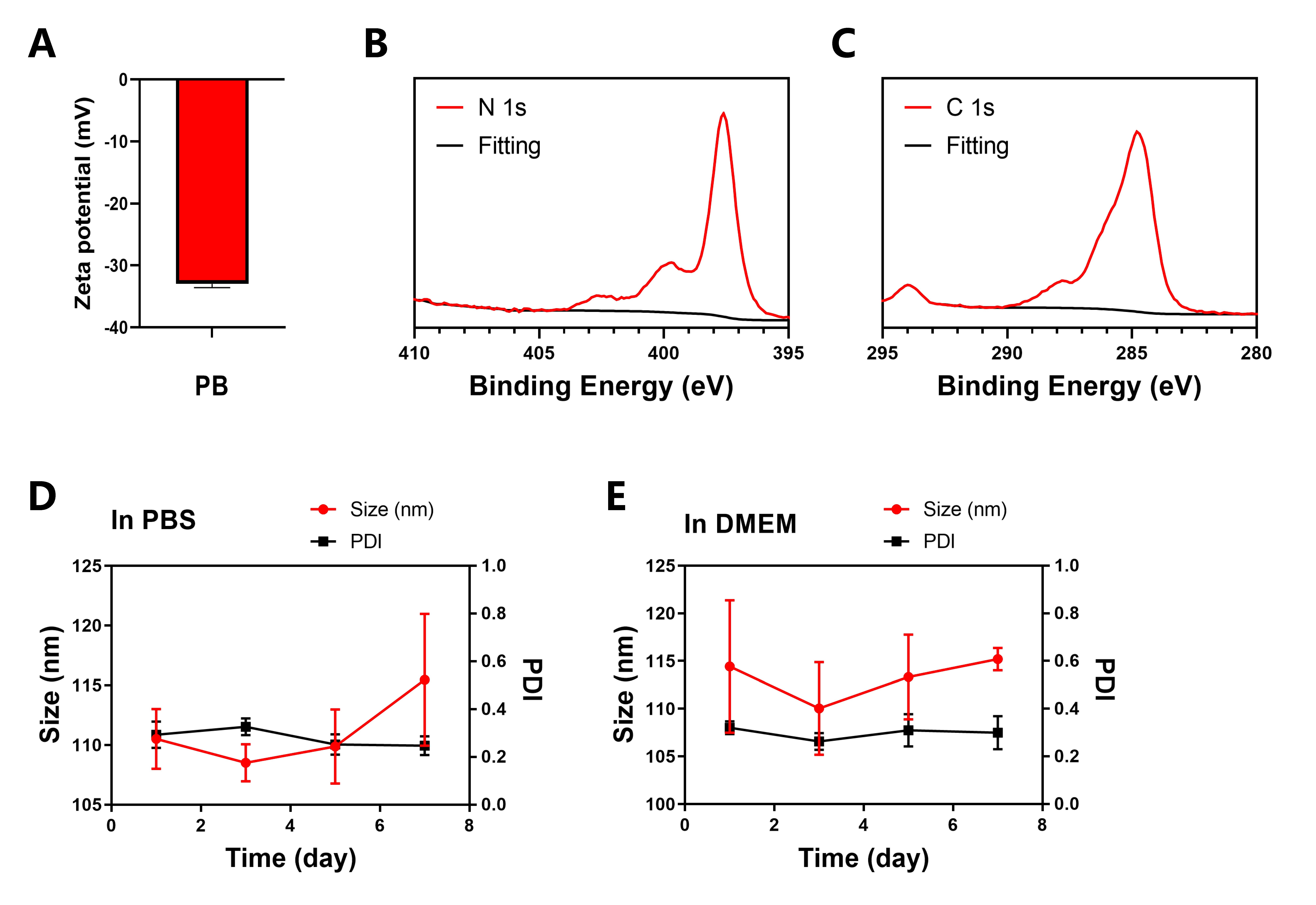
**

**Fig. S1** (A) Zeta potential of PB (n = 3, mean with SD). (B and C) XPS analysis of PB. (D) Stability of PB in PBS for seven days (n = 3, mean with SD). (E) Stability of PB in DMEM for seven days (n = 3, mean with SD). n represents the number of biologically independent samples. P values are shown in graphs with significance levels denoted as *P<0.05, **P<0.01, and ***P<0.001.


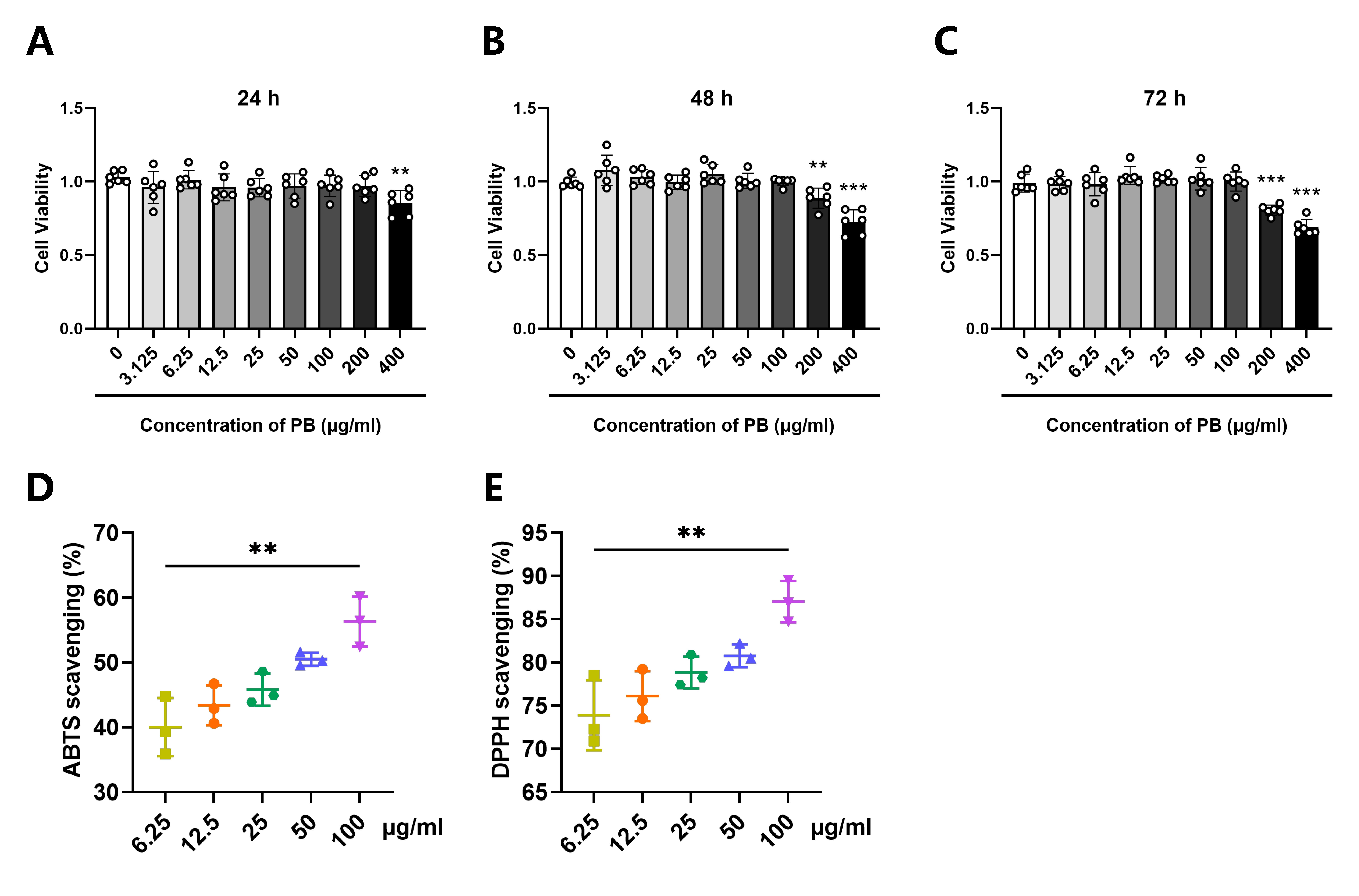


**Fig. S2** (A to C) Cell viability of BMDM treated with different concentrations of PB for 24, 48, 72 h (n = 6, mean with SD). (D) Quantitative analysis of ABTS scavenging capabilities after incubation with different concentrations of PB (n = 6, mean with SD). (E) Quantitative analysis of DPPH scavenging capabilities after incubation with different concentrations of PB (n = 6, mean with SD). n represents the number of biologically independent samples. P values are shown in graphs with significance levels denoted as *P<0.05, **P<0.01, and ***P<0.001.





**Fig. S3** (A) According to single-cell sequencing analysis, comparison of the proportions of different cells between the Naive group and the SCI Day 3 group. (B) According to single-cell sequencing analysis, comparison of the proportions of different cells between the Naive group and the SCI Day 7 group. (C) According to single-cell sequencing analysis, comparison of the proportions of different cells between the SCI Day 3group and the SCI Day 7 group.





**Fig. S4** (A) Heatmap of chemokine (C-X-C motif) ligand family (CXCL family) gene expression in spinal cord tissue at diverse periods (0, 1, 3, 7 day) after SCI (n = 3, mean with SD). (B) Heatmap of chemokine (C-C motif) ligand family (CCL family) gene expression in spinal cord tissue at diverse periods (0, 1, 3, 7 day) after SCI (n = 3, mean with SD). (C to G) Computer simulation image of protein docking between receptor-ligand. n represents the number of biologically independent samples. P values are shown in graphs with significance levels denoted as *P<0.05, **P<0.01, and ***P<0.001.


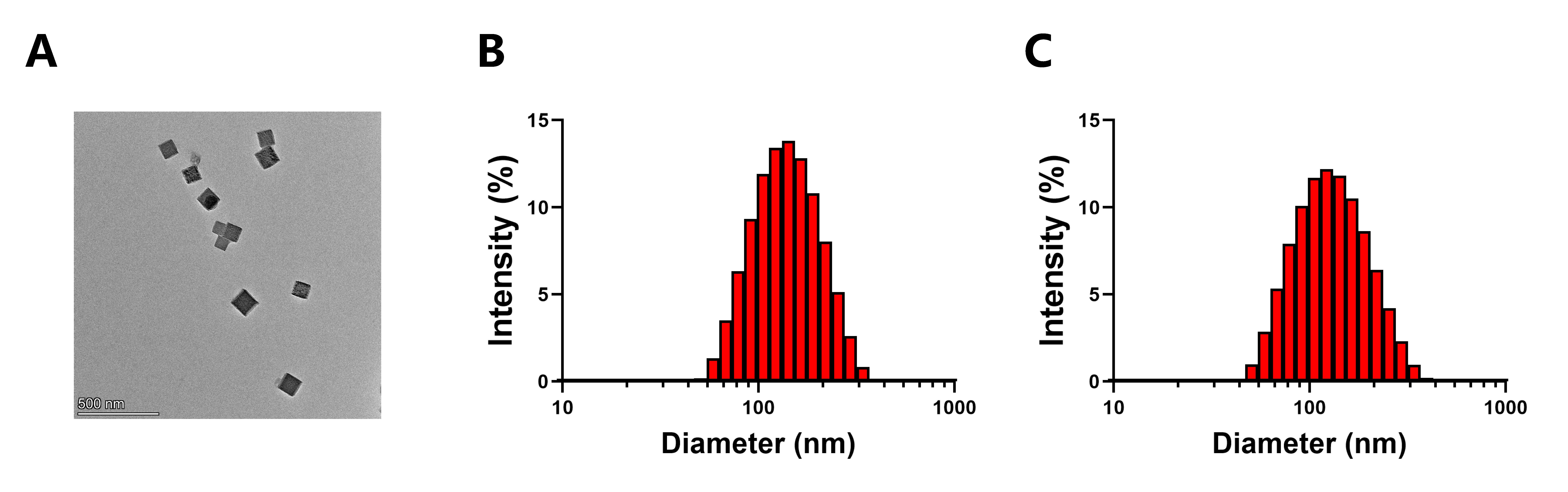


**Fig. S5** (A) TEM images of pPB. (B) Diameters of pPB (n = 3, mean with SD). (C) Diameters of pPB-siRNA^TRAF6^ (n = 3, mean with SD). n represents the number of biologically independent samples. P values are shown in graphs with significance levels denoted as *P<0.05, **P<0.01, and ***P<0.001.


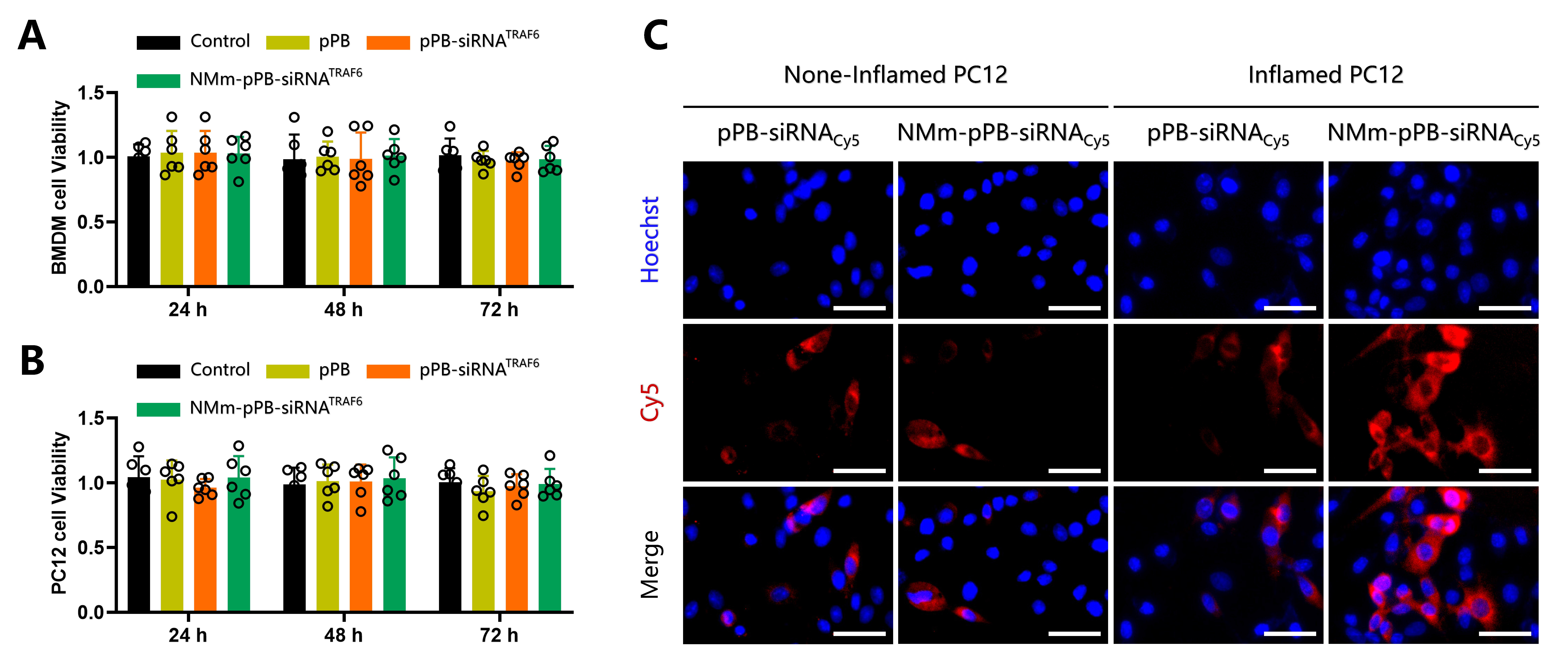


**Fig. S6** (A) Cell viability of BMDM treated with pPB, pPB-siRNA^TRAF6^, or NMm-pPB-siRNA^TRAF6^ (with PB concentration of 100 µg/mL) for 24, 48, 72 h (n = 6, mean with SD). (B) Cell viability of PC12 treated with pPB, pPB-siRNA^TRAF6^, or NMm-pPB-siRNA^TRAF6^ (with PB concentration of 100 µg/mL) for 24, 48, 72 h (n = 6, mean with SD). (C) Representative confocal images of PC12 incubated with pPB-siRNA_Cy5_ and NMm-pPB-siRNA_Cy5_ with stimulation using LPS (Inflamed PC12) or without stimulation using LPS (None-Inflamed PC12). n represents the number of biologically independent samples. P values are shown in graphs with significance levels denoted as *P<0.05, **P<0.01, and ***P<0.001. Scale bars, 50 μm (C).


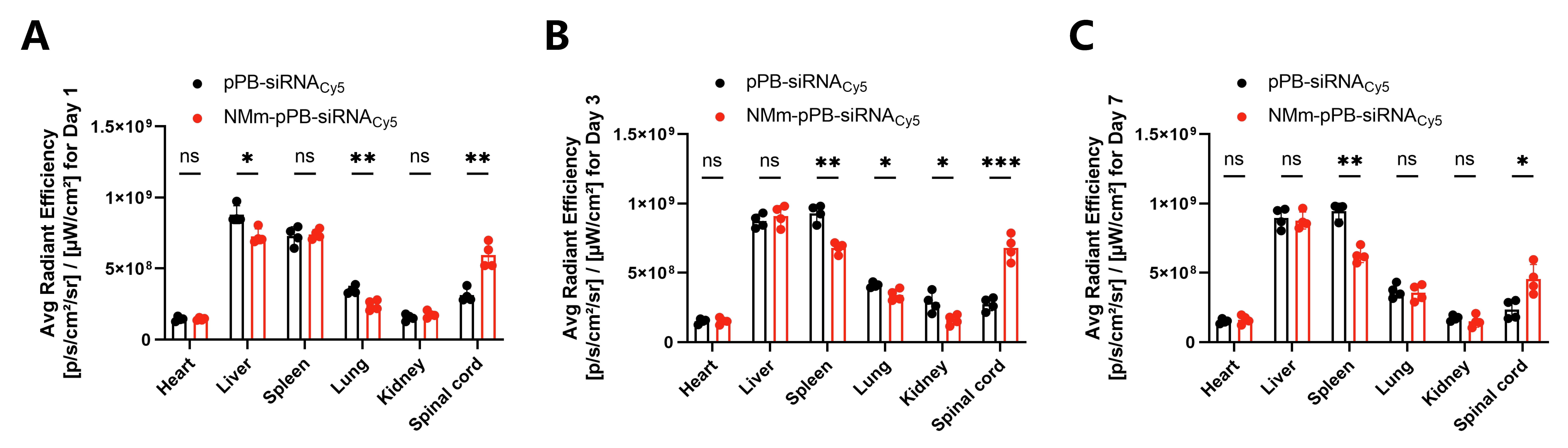


**Fig. S7** (A to C) Quantification of the fluorescence intensity of major organs (heart, liver, spleen, lungs, kidneys) and spinal cord on SCI Day 1,3, and 7 (n = 4, mean with SD). Mice were treated with pPB-siRNA_Cy5_ and NMm-pPB-siRNA_Cy5_ on SCI Day 1, 3, and 7. The spinal cord and major organs (heart, liver, spleen, lungs, kidneys) were dissected and analyzed using IVIS at the final time point for in vivo imaging. n represents the number of biologically independent samples. P values are shown in graphs with significance levels denoted as *P<0.05, **P<0.01, and ***P<0.001.





**Fig. S8** (A) Representative immunofluorescence images of longitudinal spinal cord sections from mice treated with pPB-siRNA_Cy5_ or NMm-pPB-siRNA_Cy5_ at days 1, 3, and 7 post-SCI. Nuclei were stained with DAPI (blue), neurons with NeuN (green), microglia/macrophages with Iba1 (red), and nanoparticles were labeled with Cy5 (pseudo-colored yellow). The white boxes indicate the lesion area in the spinal cord. (B) Quantitative analysis of the corresponding Cy5 fluorescence intensity in the lesion region at different time points (n = 3, mean ± SD). (C) High-magnification images of the regions, illustrating the co-localization distribution of Cy5-labeled nanoparticles (yellow) with Iba1+ (red) and NeuN+ (green) cells. The white arrows indicate nanoparticles internalized by the corresponding cells. n represents the number of biologically independent samples. P values are shown in graphs with significance levels denoted as *P<0.05, **P<0.01, and ***P<0.001. Scale bars, 700 µm (A) and 50 µm (C).


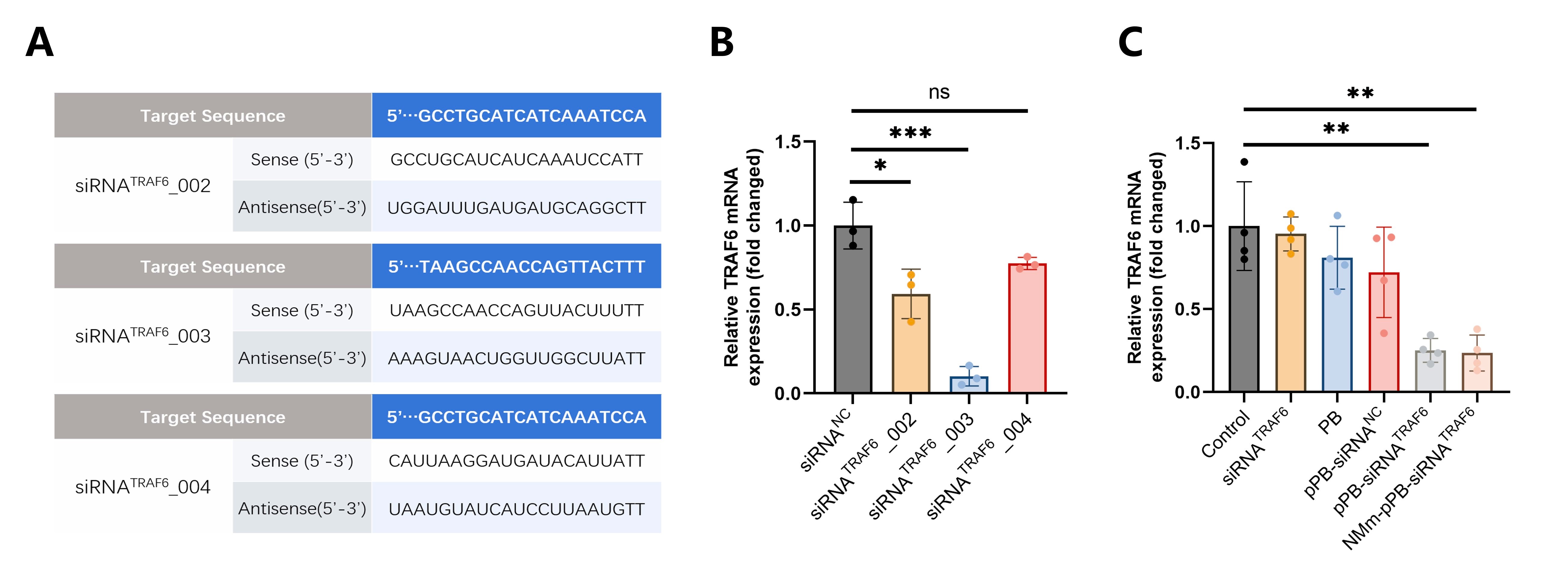


**Fig. S9** (A) Designed siRNA^TRAF6^ sequences (siRNA^TRAF6^_002, siRNA^TRAF6^_003, and siRNA^TRAF6^_004) that may have silencing effects. (B) Silencing efficiency of siRNA^TRAF6^_002, siRNA^TRAF6^_003, and siRNA^TRAF6^_004 in BMDMs was determined by measuring the TRAF6 gene expression by qRT-PCR (n = 3, mean with SD). Transfection was performed using Lipofectamine™ 3000, following the manufacturer's instructions. (C) Silencing efficiency of free siRNA^TRAF6^, PB, pPB-siRNA^NC^, pPB-siRNA^TRAF6^, and NMm-pPB-siRNA^TRAF6^ in BMDMs was determined by measuring the TRAF6 gene expression by qRT-PCR (n = 3, mean with SD). n represents the number of biologically independent samples. P values are shown in graphs with significance levels denoted as *P<0.05, **P<0.01, and ***P<0.001.


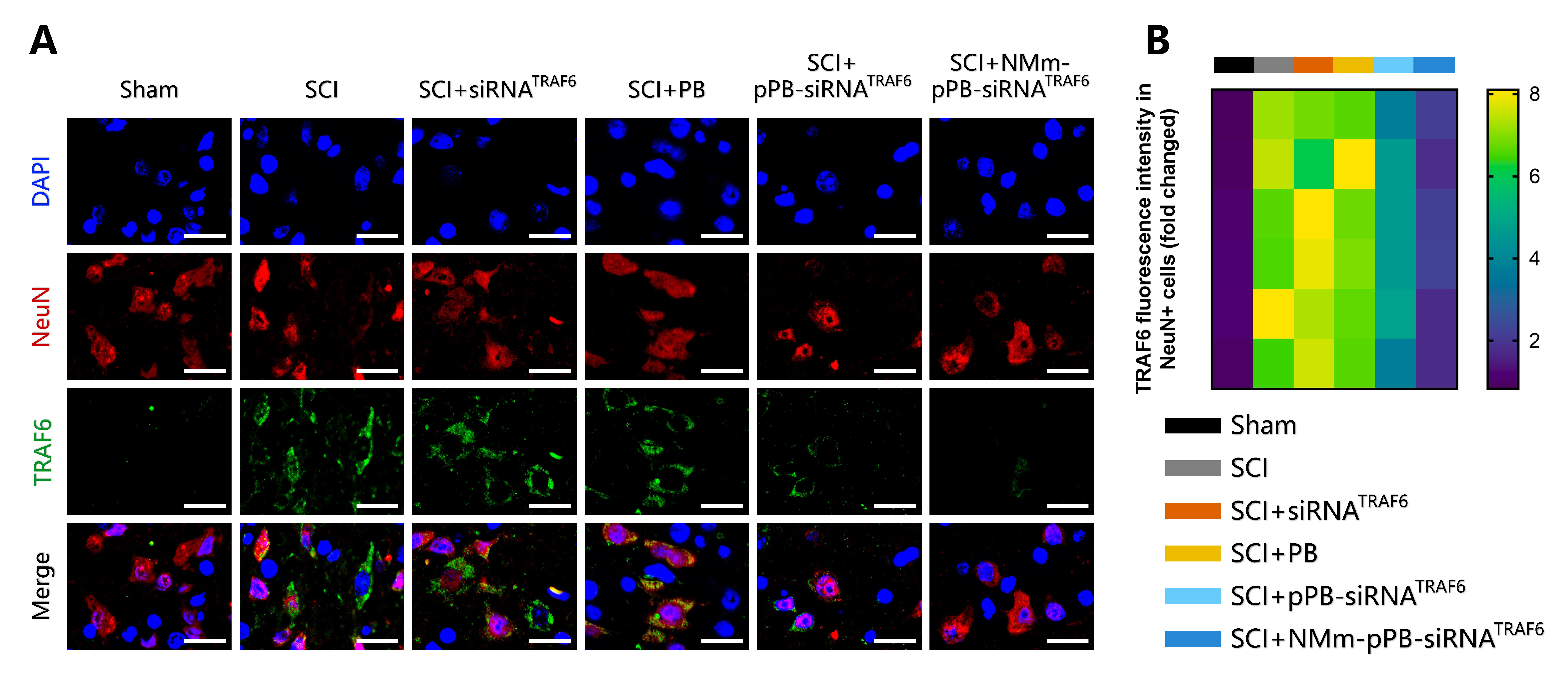


**Fig. S10** (A and B) Double-immunofluorescence staining and quantitative fluorescence intensity analysis of TRAF6 and NeuN in the spinal cords of the Sham, SCI, SCI+siRNA^TRAF6^, SCI+PB, SCI+pPB-siRNA^TRAF6^, SCI+NMm-pPB-siRNA^TRAF6^ groups at Day 7 (n = 6, mean with SD). n represents the number of biologically independent samples. P values are shown in graphs with significance levels denoted as *P<0.05, **P<0.01, and ***P<0.001. Scale bars, 20 μm (A).


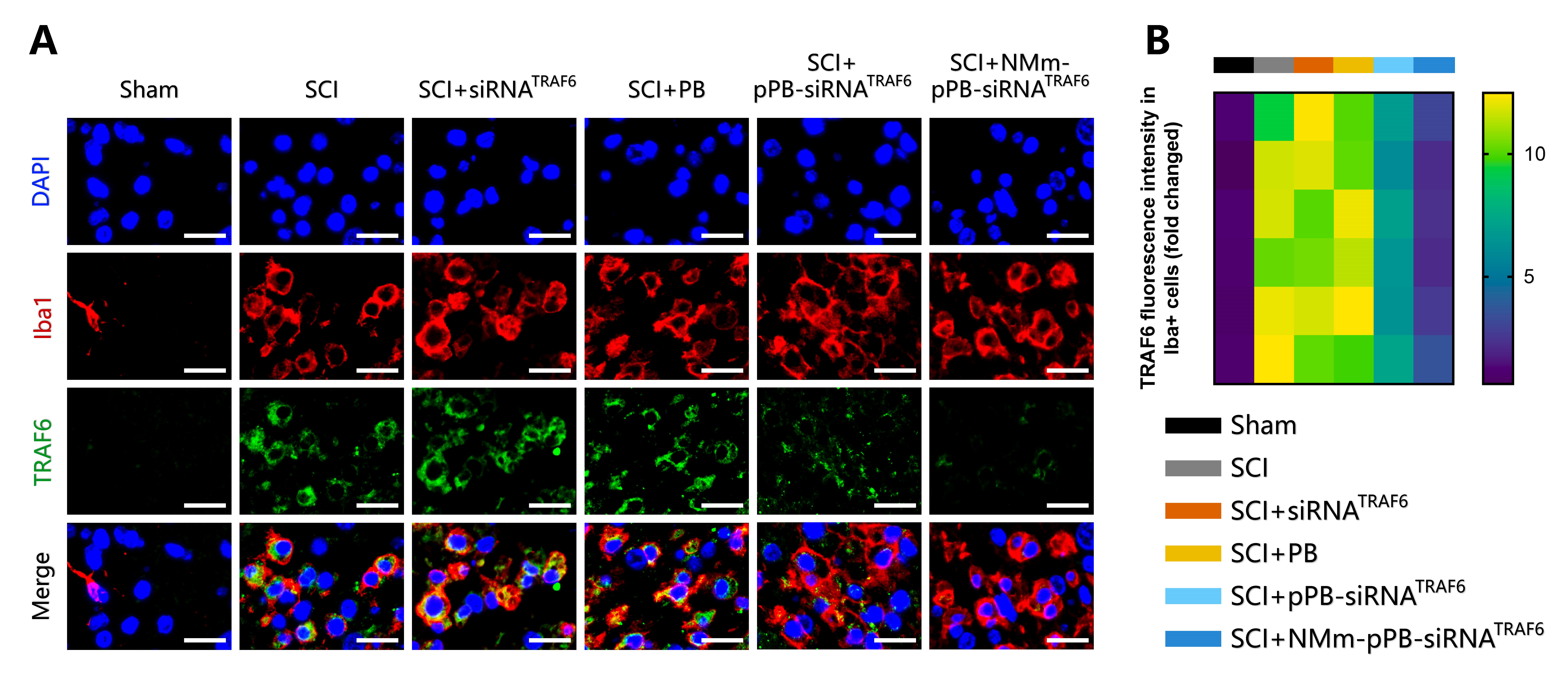


**Fig. S11** (A and B) Double-immunofluorescence staining and quantitative fluorescence intensity analysis of TRAF6 and Iba1 in the spinal cords of the Sham, SCI, SCI+siRNA^TRAF6^, SCI+PB, SCI+pPB-siRNA^TRAF6^, SCI+NMm-pPB-siRNA^TRAF6^ groups at Day 7 (n = 6, mean with SD). n represents the number of biologically independent samples. P values are shown in graphs with significance levels denoted as *P<0.05, **P<0.01, and ***P<0.001. Scale bars, 20 μm (A).


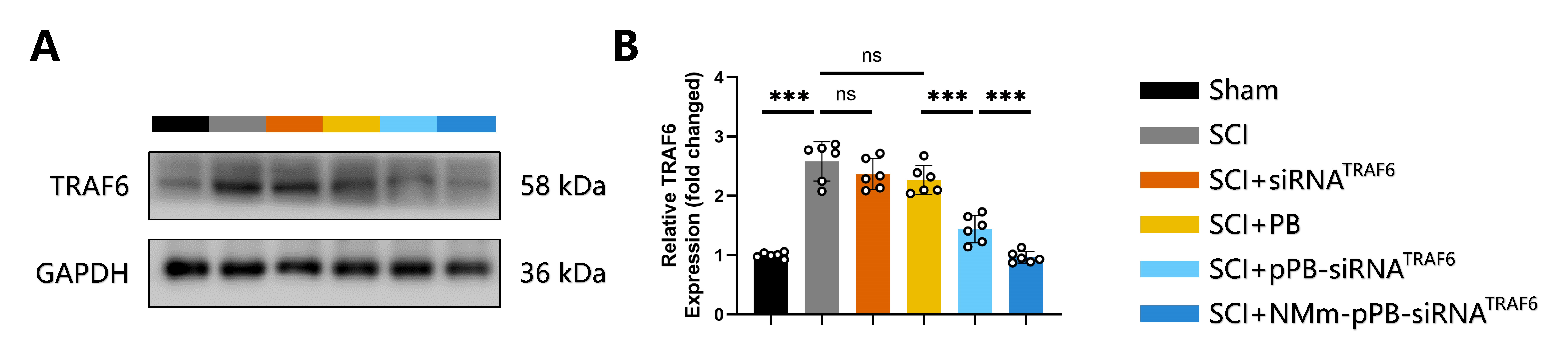


**Fig. S12** (A and B) Western blot analysis and relative quantification of TRAF6 protein levels in the spinal cords of the Sham, SCI, SCI+siRNA^TRAF6^, SCI+PB, SCI+pPB-siRNA^TRAF6^, SCI+NMm-pPB-siRNA^TRAF6^ groups at Day 7 (n = 6, mean with SD). n represents the number of biologically independent samples. P values are shown in graphs with significance levels denoted as *P<0.05, **P<0.01, and ***P<0.001.


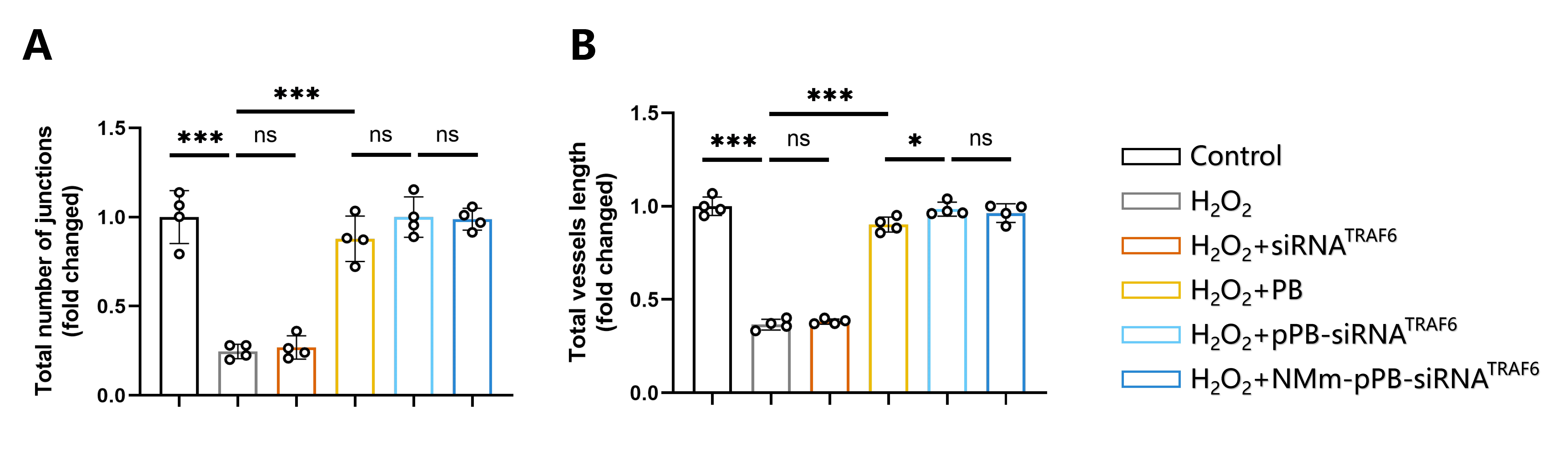


**Fig. S13** (A and B) Tube formation assay was performed to evaluate the tube formation ability of HUVECs under treatment of H_2_O_2_, H_2_O_2_ and siRNA^TRAF6^, H_2_O_2_ and PB, H_2_O_2_ and pPB-siRNA^TRAF6^, or H_2_O_2_ and NMm-pPB-siRNA^TRAF6^. Total number of junctions and total vessels length were measured by the angiogenic analysis plugin for Image J (n = 4, mean with SD). n represents the number of biologically independent samples. P values are shown in graphs with significance levels denoted as *P<0.05, **P<0.01, and ***P<0.001.


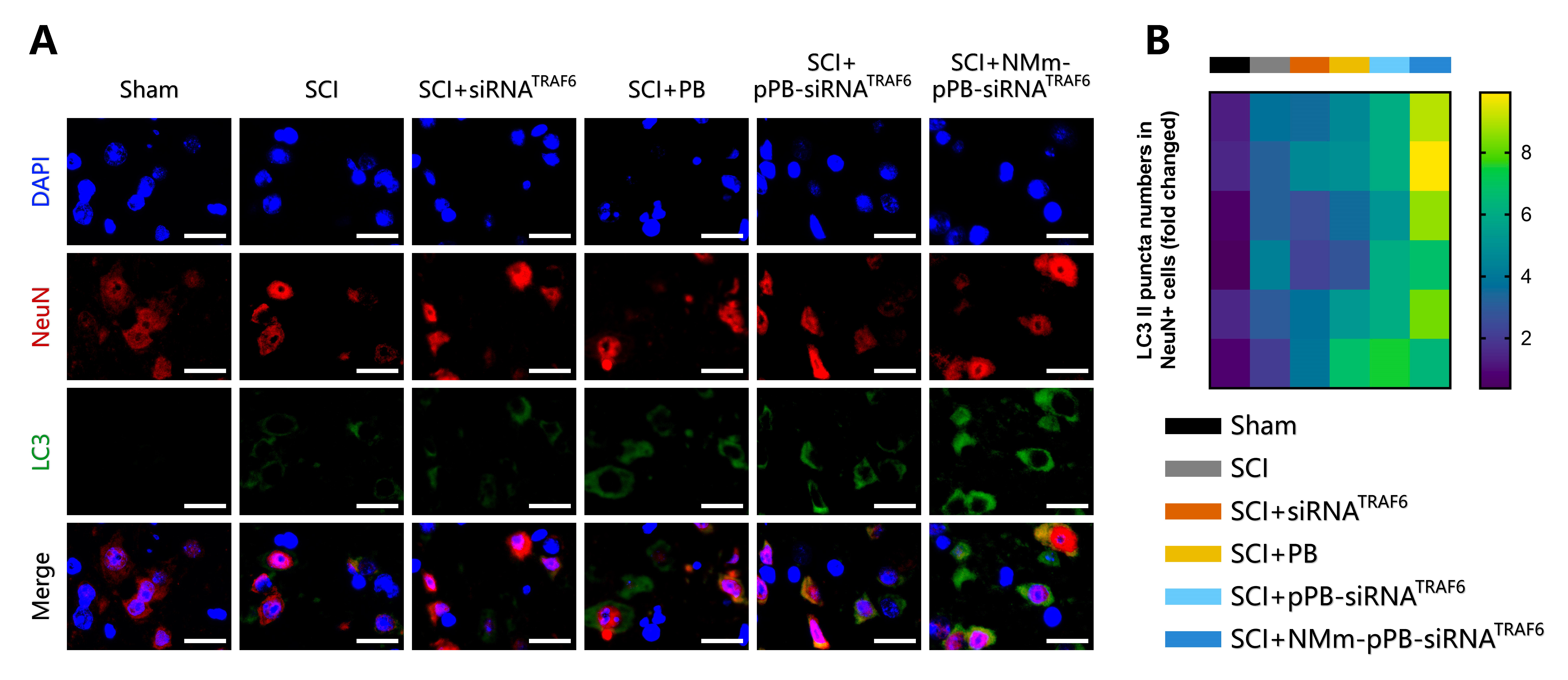


**Fig. S14** (A and B) Double-immunofluorescence staining and quantitative puncta numbers of LC3 and NeuN in the spinal cords of the Sham, SCI, SCI+siRNA^TRAF6^, SCI+PB, SCI+pPB-siRNA^TRAF6^, SCI+NMm-pPB-siRNA^TRAF6^ groups at Day 7 (n = 6, mean with SD). n represents the number of biologically independent samples. P values are shown in graphs with significance levels denoted as *P<0.05, **P<0.01, and ***P<0.001. Scale bars, 20 μm (A).


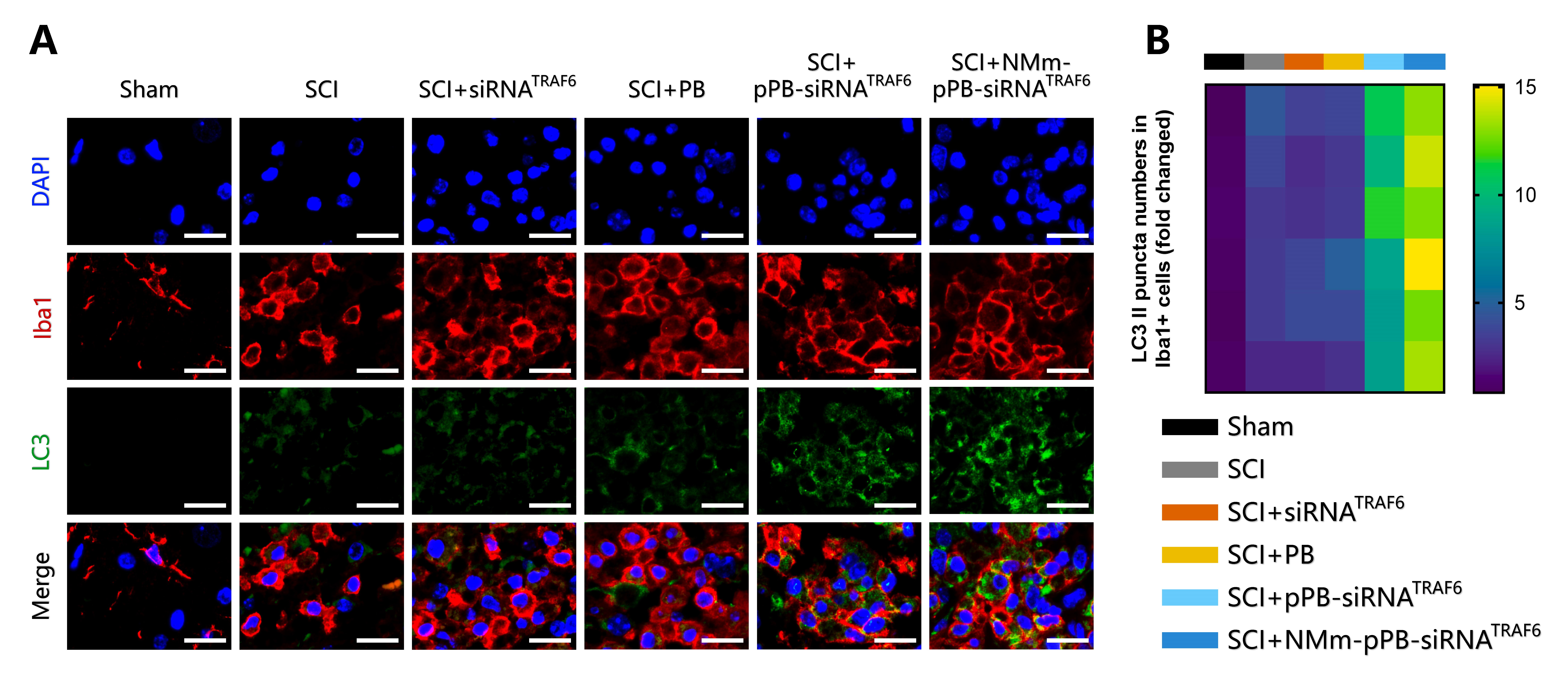


**Fig. S15** (A and B) Double-immunofluorescence staining and quantitative puncta numbers of LC3 and Iba1 in the spinal cords of the Sham, SCI, SCI+siRNA^TRAF6^, SCI+PB, SCI+pPB-siRNA^TRAF6^, SCI+NMm-pPB-siRNA^TRAF6^ groups at Day 7 (n = 6, mean with SD). n represents the number of biologically independent samples. P values are shown in graphs with significance levels denoted as *P<0.05, **P<0.01, and ***P<0.001. Scale bars, 20 μm (A).


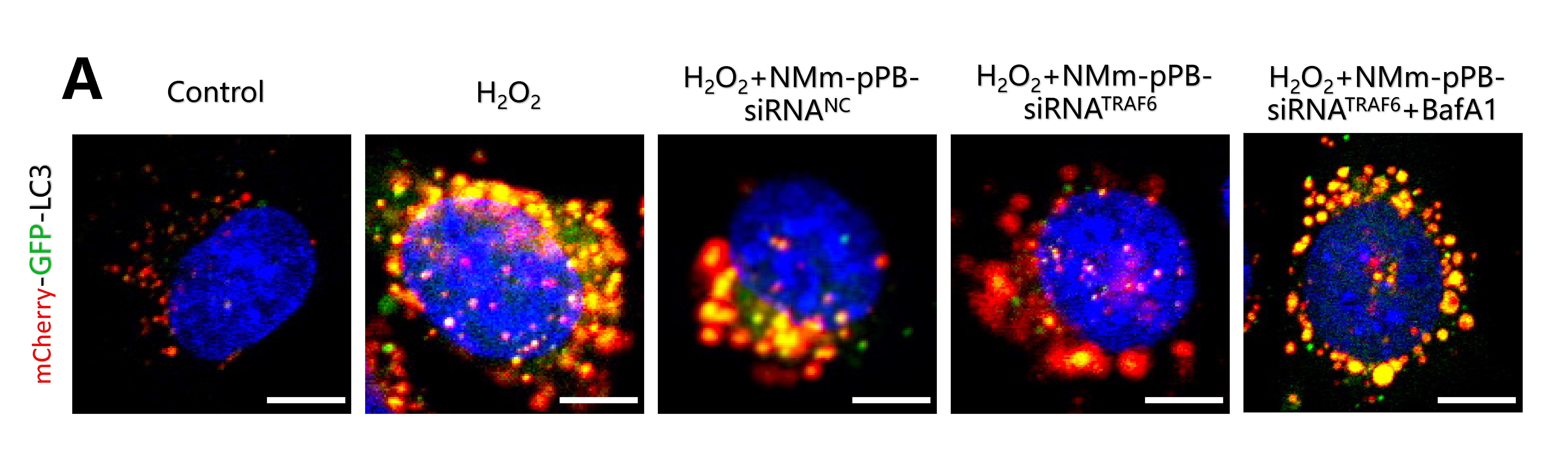


**Fig. S16** (A) Representative confocal fluorescence images of PC12 cells transfected with Ad-mCherry-GFP-LC3B. The cells were subjected to five experimental conditions: Control, H_2_O_2_, H_2_O_2_+NMm-pPB-siRNA^NC^, H_2_O_2_+NMm-pPB-siRNA^TRAF6^, and H_2_O_2_+NMm-pPB-siRNA^TRAF6^+Baf A1. Yellow puncta (GFP+/mCherry+) represent autophagosomes indicative of blocked flux or early autophagy, whereas red puncta (GFP-/mCherry+) represent autolysosomes indicative of smooth autophagic flux.


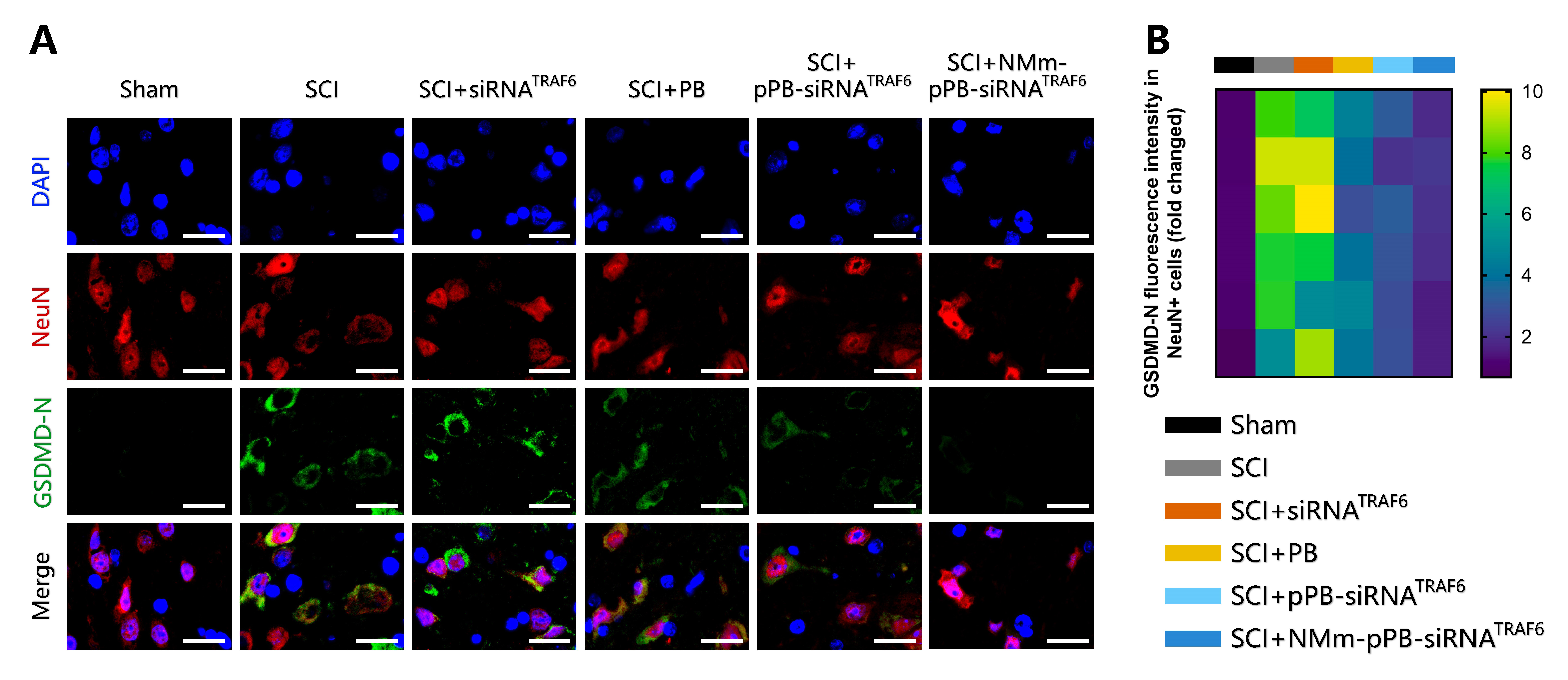


**Fig. S17** (A and B) Double-immunofluorescence staining and quantitative fluorescence intensity analysis of GSDMD-N and NeuN in the spinal cords of the Sham, SCI, SCI+siRNA^TRAF6^, SCI+PB, SCI+pPB-siRNA^TRAF6^, SCI+NMm-pPB-siRNA^TRAF6^ groups at Day 7 (n = 6, mean with SD). n represents the number of biologically independent samples. P values are shown in graphs with significance levels denoted as *P<0.05, **P<0.01, and ***P<0.001. Scale bars, 20 μm (A).


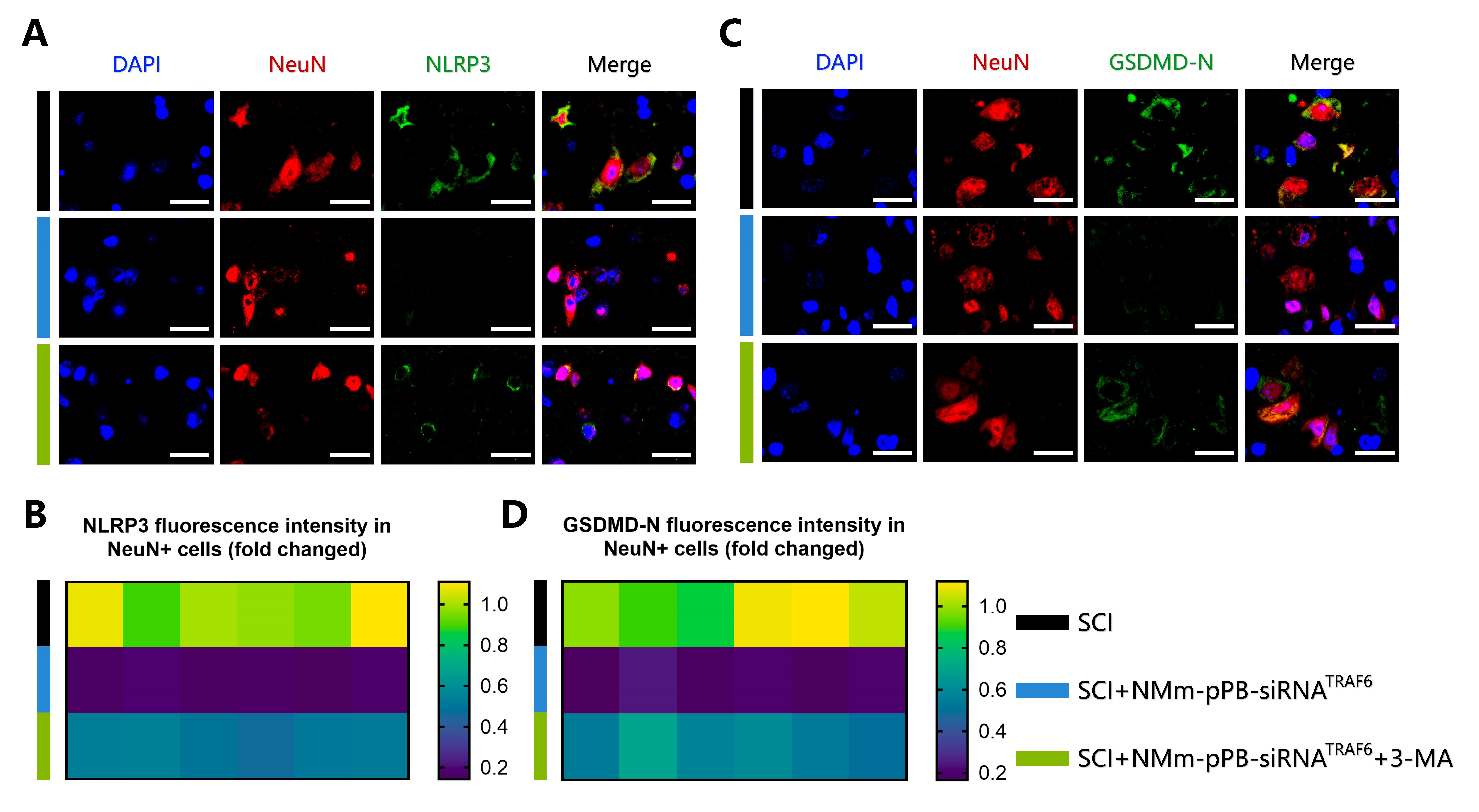


**Fig. S18** (A and B) Double-immunofluorescence staining and quantitative fluorescence intensity analysis of NLRP3 and NeuN in the spinal cords of the SCI, SCI+NMm-pPB-siRNA^TRAF6^, SCI+NMm-pPB-siRNA^TRAF6^+3-MA groups at Day 7 (n = 6, mean with SD). (C and D) Double-immunofluorescence staining and quantitative fluorescence intensity analysis of GSDMD-N and NeuN in the spinal cords of the SCI, SCI+NMm-pPB-siRNA^TRAF6^, SCI+NMm-pPB-siRNA^TRAF6^+3-MA groups at Day 7 (n = 6, mean with SD). Note, 3-MA was administered via intraperitoneal injection half an hour before the tail vein injection of NMm-pPB-siRNA^TRAF6^ nanoparticles. n represents the number of biologically independent samples. P values are shown in graphs with significance levels denoted as *P<0.05, **P<0.01, and ***P<0.001. Scale bars, 20 μm (A and C).


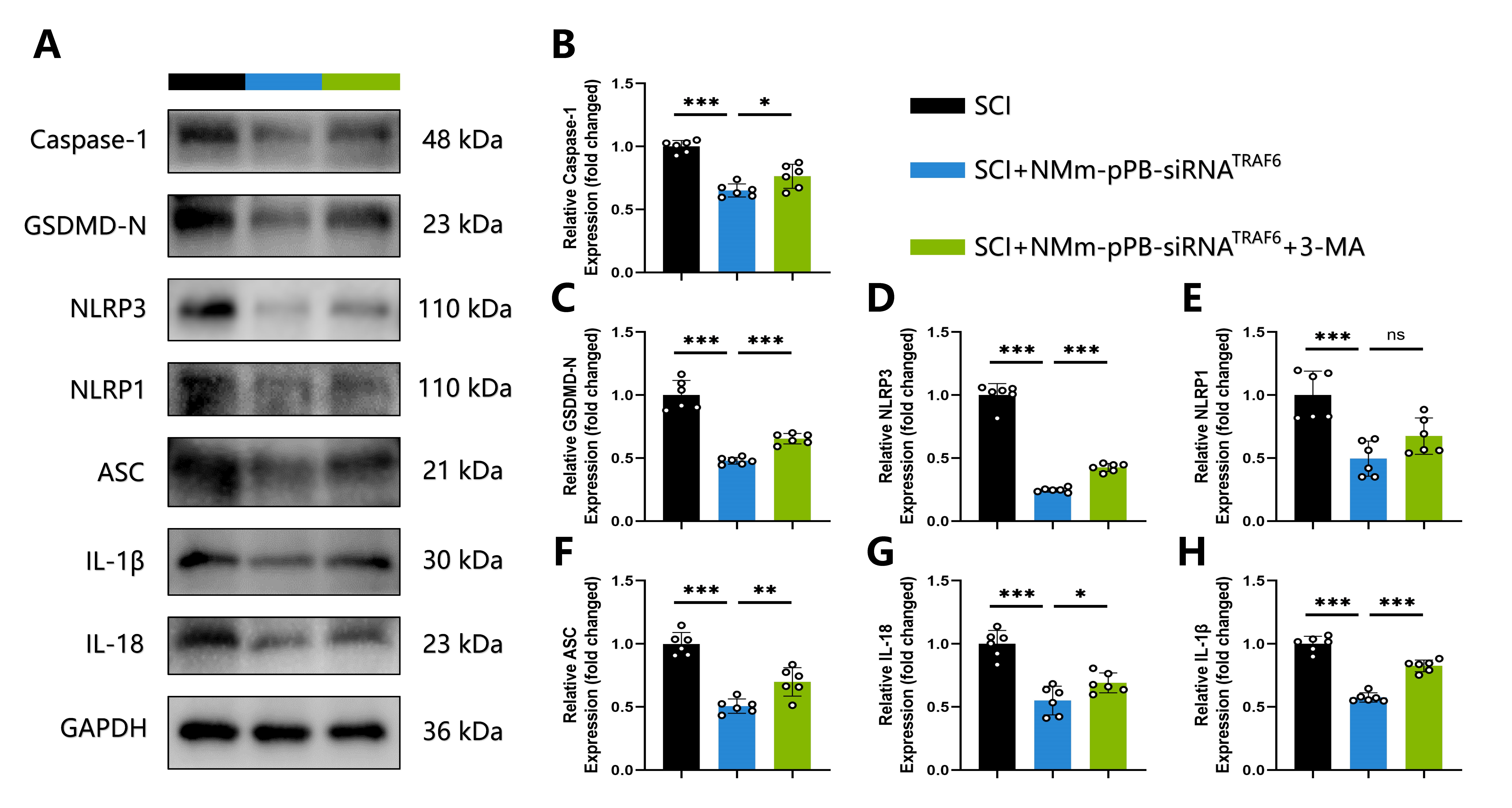


**Fig. S19** (A to H) Western blot analysis and relative quantification of Caspase-1, GSDMD-N, NLRP3, NLRP1, ASC, IL-1β and IL-18 protein levels in the spinal cords of the SCI, SCI+NMm-pPB-siRNA^TRAF6^, SCI+NMm-pPB-siRNA^TRAF6^+3-MA groups at Day 7 (n = 6, mean with SD). Note, 3-MA was administered via intraperitoneal injection half an hour before the tail vein injection of NMm-pPB-siRNA^TRAF6^ nanoparticles. n represents the number of biologically independent samples. P values are shown in graphs with significance levels denoted as *P<0.05, **P<0.01, and ***P<0.001.


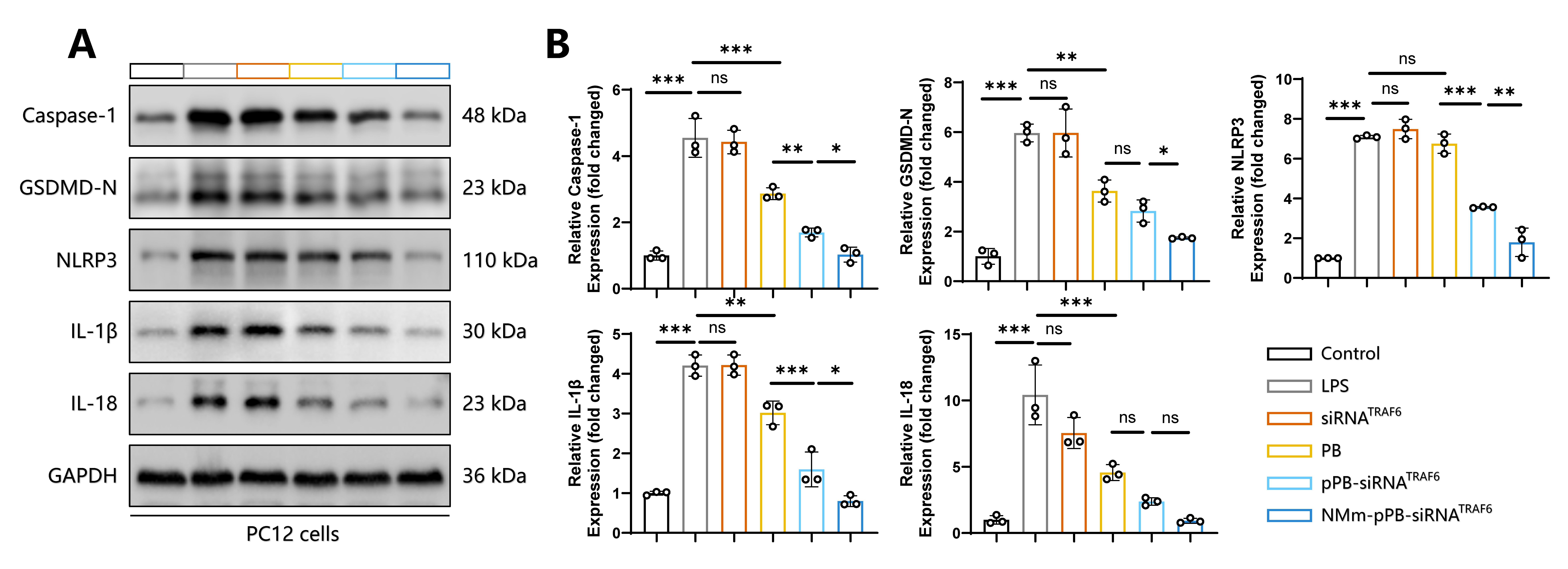


**Fig. S20** (A) Western blot analysis of pyroptosis-related proteins (Caspase-1, GSDMD-N, NLRP3, IL-1β, and IL-18) in PC12 cells. Cells were stimulated with LPS to induce inflammation and treated with free siRNA^TRAF6^, PB, pPB-siRNA^TRAF6^, or NMm-pPB-siRNA^TRAF6^. GAPDH was used as the loading control. (B) Relative quantification of protein levels (n = 3, mean ± SD). n represents the number of biologically independent samples. P values are shown in graphs with significance levels denoted as *P<0.05, **P<0.01, and ***P<0.001.

**
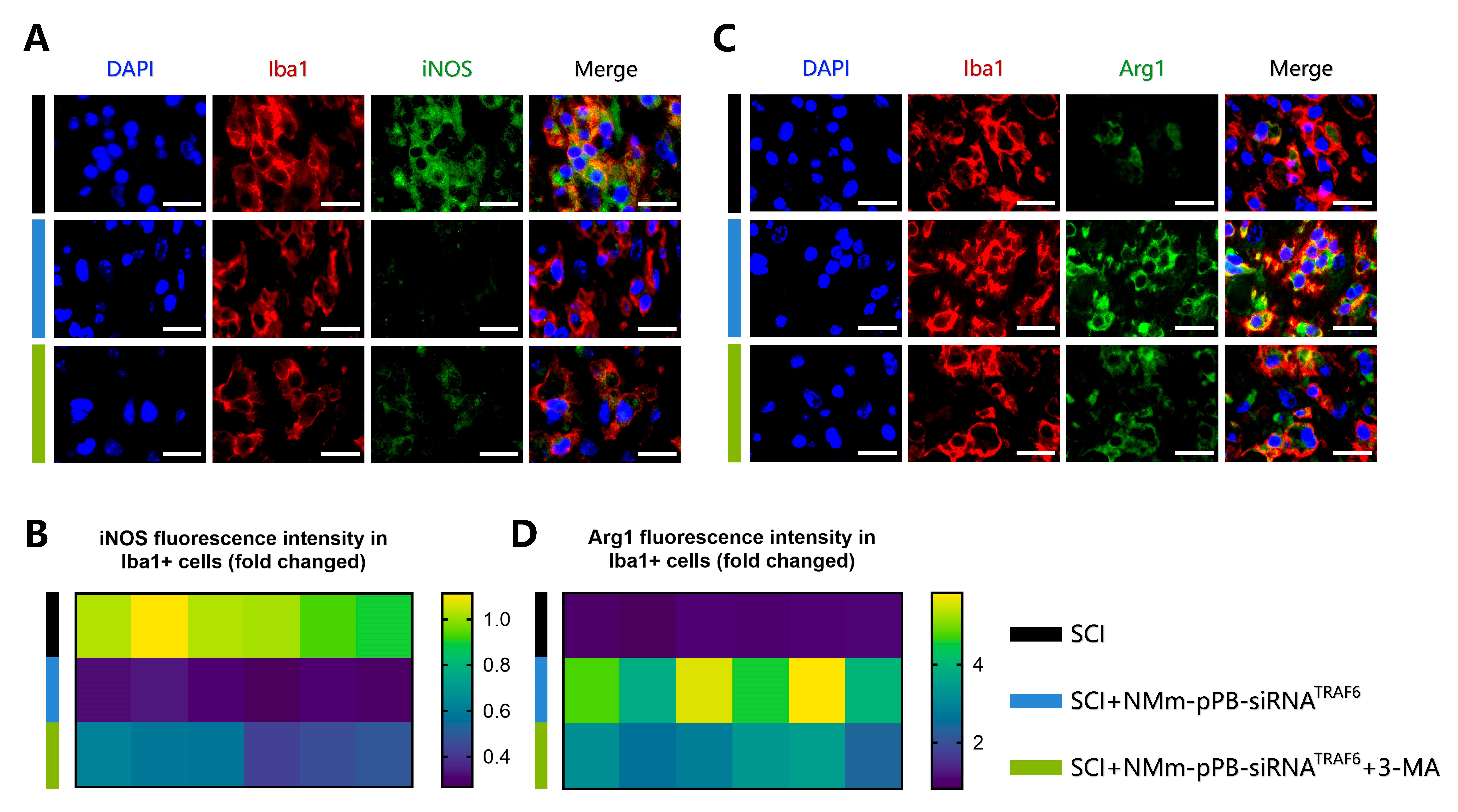
**

**Fig. S21** (A and B) Double-immunofluorescence staining and quantitative fluorescence intensity analysis of iNOS and Iba1 in the spinal cords of the SCI, SCI+NMm-pPB-siRNA^TRAF6^, SCI+NMm-pPB-siRNA^TRAF6^+3-MA groups at Day 7 (n = 6, mean with SD). (C and D) Double-immunofluorescence staining and quantitative fluorescence intensity analysis of Arg1 and Iba1 in the spinal cords of the SCI, SCI+NMm-pPB-siRNA^TRAF6^, SCI+NMm-pPB-siRNA^TRAF6^+3-MA groups at Day 7 (n = 6, mean with SD). Note, 3-MA was administered via intraperitoneal injection half an hour before the tail vein injection of NMm-pPB-siRNA^TRAF6^ nanoparticles. n represents the number of biologically independent samples. P values are shown in graphs with significance levels denoted as *P<0.05, **P<0.01, and ***P<0.001. Scale bars, 20 μm (A and C).


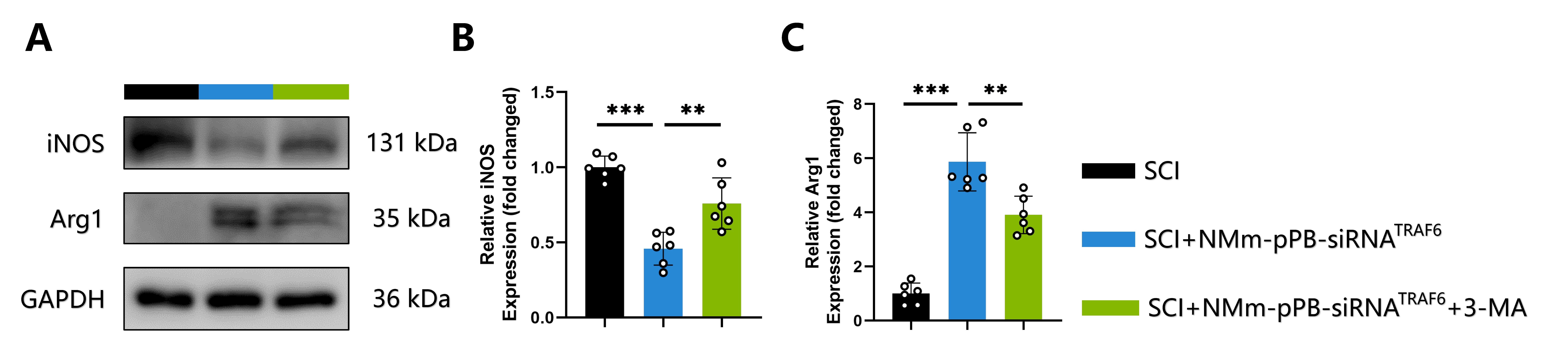


**Fig. S22** (A to C) Western blot analysis and relative quantification of iNOS and Arg1 protein levels in the spinal cords of the SCI, SCI+NMm-pPB-siRNA^TRAF6^, SCI+NMm-pPB-siRNA^TRAF6^+3-MA groups at Day 7 (n = 6, mean with SD). n represents the number of biologically independent samples. Note, 3-MA was administered via intraperitoneal injection half an hour before the tail vein injection of NMm-pPB-siRNA^TRAF6^ nanoparticles. n represents the number of biologically independent samples. P values are shown in graphs with significance levels denoted as *P<0.05, **P<0.01, and ***P<0.001.


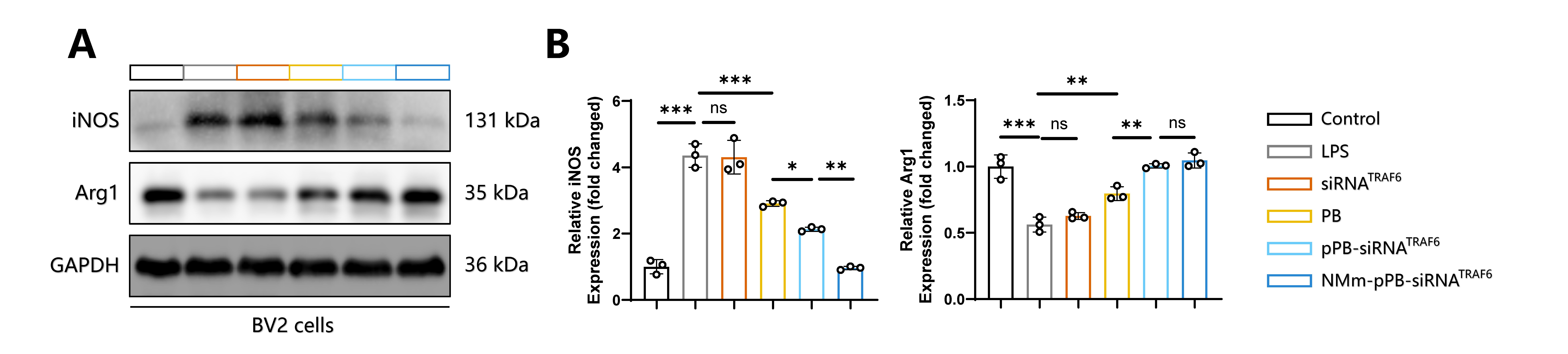


**Fig. S23** (A) Western blot analysis of the M1 phenotype marker (iNOS) and M2 phenotype marker (Arg1) in BV2 microglia cells. Cells were stimulated with LPS to induce inflammation and treated with free siRNA^TRAF6^, PB, pPB-siRNA^TRAF6^, or NMm-pPB-siRNA^TRAF6^. GAPDH was used as the loading control. (B) Relative quantification of protein levels (n = 3, mean ± SD). n represents the number of biologically independent samples. P values are shown in graphs with significance levels denoted as *P<0.05, **P<0.01, and ***P<0.001.





**Fig. S24** (A to C) qRT-PCR analysis of the gene expression of immature neuron markers (Tuj1, Dcx and Stmn1) (n = 6, mean with SD). (D) qRT-PCR analysis of the gene expression of mature neuron markers (Map2) (n = 6, mean with SD). (E to G) qRT-PCR analysis of the gene expression of interneuron markers (Htr3a, Calb1 and Gad2) (n = 6, mean with SD). (H) qRT-PCR analysis of the gene expression of sensory neuron markers (Sox10) (n = 6, mean with SD). (I and J) qRT-PCR analysis of the gene expression of motor neuron markers (Chat and Mnx1) (n = 6, mean with SD). (K and L) qRT-PCR analysis of the gene expression of blood vessel markers (CD31 and Vcam1) (n = 6, mean with SD). n represents the number of biologically independent samples. P values are shown in graphs with significance levels denoted as *P<0.05, **P<0.01, and ***P<0.001.


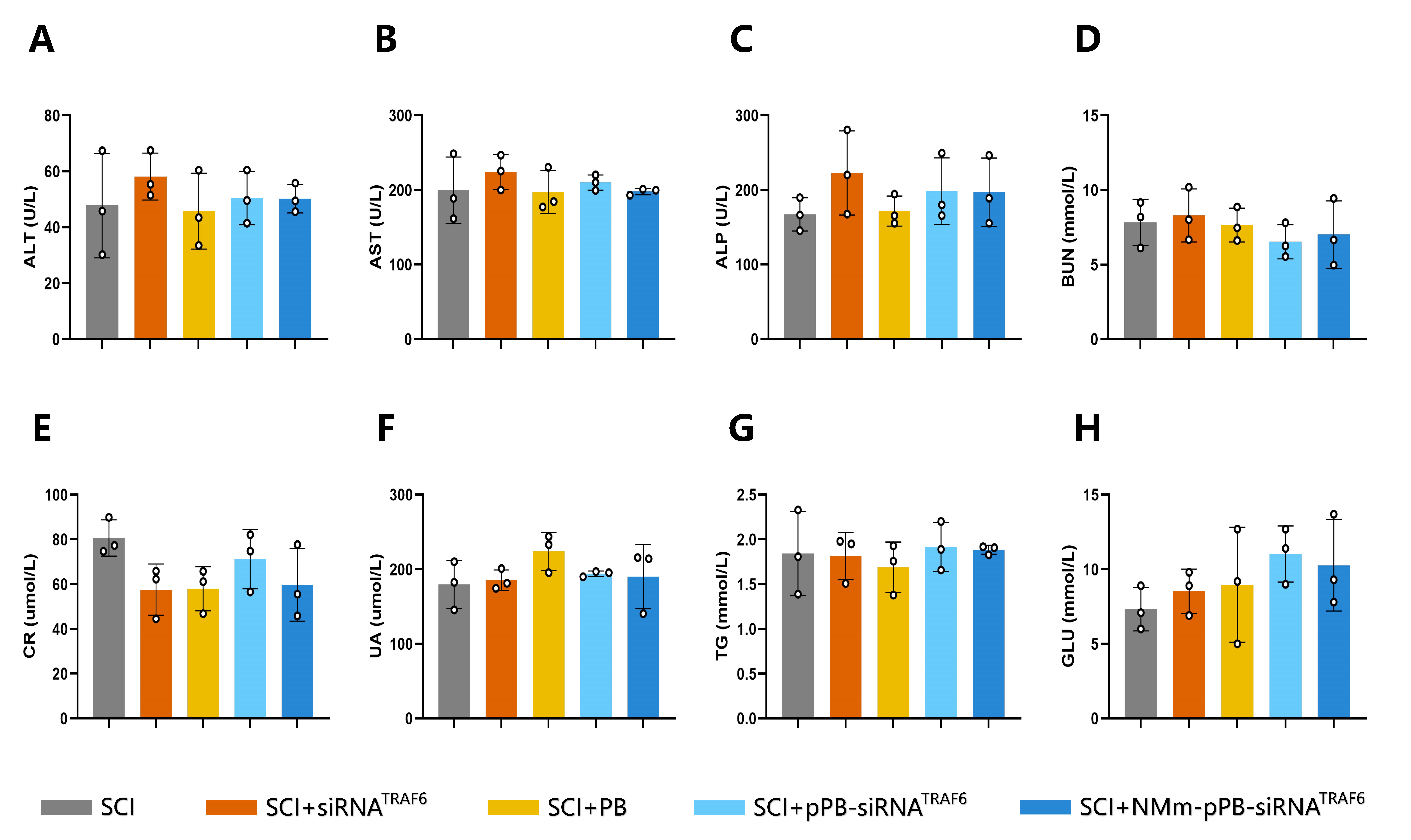


**Fig. S25** (A to C) Liver toxicity evaluated using values of alanine aminotransferase (ALT), aspartate aminotransferase (AST) and alkaline phosphatase (ALP) in the mouse serum (n = 3, mean with SD). (D to F) Kidney toxicity evaluated using blood urea nitrogen (BUN), creatinine (CR) and uric Acid (UA) in the mouse serum (n = 3, mean with SD). (G) Triglycerides (TG) was used to assess blood lipid levels (n = 3, mean with SD). (H) Glucose (GLU) was used to measure blood glucose levels (n = 3, mean with SD). Note, we obtained mouse serum samples using standard procedures for relevant testing at SCI Day 7 (short-term experiments). n represents the number of biologically independent samples. P values are shown in graphs with significance levels denoted as *P<0.05, **P<0.01, and ***P<0.001.





**Fig. S26** (A) H&E staining of histological sections major organs (heart, liver, spleen, lungs, kidneys) of the SCI, SCI+siRNA^TRAF6^, SCI+PB, SCI+pPB-siRNA^TRAF6^, SCI+NMm-pPB-siRNA^TRAF6^ groups at Day 28. Scale bars, 100 μm (A).

**Supplementary Table 1 to Supplementary Table 2**

Table. S1 The primers sequences used in this study (mouse).

| Genes | Forward primer sequence (5’-3’) | Reverse primer sequence (5’-3’) |
| --- | --- | --- |
| TRAF6 | CCGGGAGCTGACTGCCAAAA | GCGCATGCACAGCTTGTACC |
| CXCL1 | AGCACTCCAGACTCCAGCCA | GGGAGCTTCAGGGTCAAGGC |
| CXCL2 | GGCTACAGGGGCTGTTGTGG | ACCGCCCTTGAGAGTGGCTA |
| CXCL3 | CAGCCACACTCCAGCCTAGC | CCACAACAGCCCCTGTAGCC |
| CXCL5 | CAGCGGTTCCATCTCGCCAT | CAGCTCCGTTGCGGCTATGA |
| CXCL9 | AGGTGTACACAGGCCCCCAT | GGCCCTGTCCACACTTCCAC |
| CXCL10 | TTCCGGAAGCCTCCCCATCA | GCCCTCATTCTCACTGGCCC |
| CXCL12 | GAGCAGAGTCCGAGGAACGC | TGCGTTTGGAGGCAAGCAGA |
| CXCL13 | GCAACGCTGCTTCTCCTCCT | TCCCAGGGGGCGTAACTTGA |
| CXCL14 | CAGCACTACTGCTCCGCTCC | ACAGGGGAATGAGCTGGGGT |
| CXCL16 | CTGGCACCCAGATACCGCAG | GGTCTTGGCTTCCCCCACAC |
| CXCL17 | GGCCACCAAGGAAGCAGTGT | TGGGGTGCTCTTGGTGAGGA |
| CCL2 | AGAGCCAGACGGGAGGAAGG | GAACAGCAGGCCCAGAAGCA |
| CCL3 | CCTGCCACCTGCATAGCTCC | TGGGAGGGAGATGGGGGTTG |
| CCL4 | TGCGTGTCTGCCCTCTCTCT | AGGGCTCACTGGGGTTAGCA |
| CCL5 | CTCGTGCCCACGTCAAGGAG | TTCCCAGGACCGAGTGGGAG |
| CCL6 | GGAGGGGAACCCAGGTCTGT | AGTGGGTGACAATGCCTGCC |
| CCL7 | TCTCTGCCACGCTTCTGTGC | GATGGGCTTCAGCGCAGACT |
| CCL8 | CGCAGTGCTTCTTTGCCTGC | AGCACCCGAAGGGGGATCTT |
| CCL9 | GCCTGAAACCTGGCTGCTCA | CAGGTGACCACGTGTTGGCT |
| CCL11 | TCGGGAGAGCCTACAGAGCC | TTGGGATGGAGCCTGGGTGA |
| TUJ1 | GATGGAGTTCACCGAGGCCG | CAAAGCTGGGGGCAGTGTCA |
| DCX | GCAGCAACAGTGCTCAAGCC | GTTGAGAAGCACGCGCACAG |
| STMN1 | GAGCAGGGCTTTCCTTGCCA | CTTCTCCGCGAGCTGCTTCA |
| MAP2 | GCCGACGAGCGGAAAGATGA | AAATCCATTGGCGTTGCGGC |
| HTR3A | ACCCCAAGGGGCAGAGGTAG | GCCTGTCCAGCACGTATCCC |
| CALB1 | CGACGCTGACGGAAGTGGTT | TCGATGAAGCCGCTGTGGTC |
| GAD2 | GACCAGCGCCAGTCTAGCAG | CAGCTTGTTTCCGATGCCGC |
| SOX10 | GCCTGGATCTCCAAGCCACC | GCGATCTGGGAAGTGGACGG |
| CHAT | GCAGCCAGCCTCATCTCTGG | CACGATGACATGCTCGGGCT |
| MNX1 | GGTGCCAGCACCTTCCAACT | ACAGCTGCTGGCTGGTGAAG |
| CD31 | AGCTCTGGGAACGAGAGCCA | GGACGTGCACTGCCTTGACT |
| VCAM1 | CCTCGTGGGACTGGATTCGC | GTGTCGGCATCCGAGTGAGG |

Table. S2 Primary antibodies and secondary antibodies used in this study.

| Application | Antibodies | Name | Source | Identifier |
| --- | --- | --- | --- | --- |
| Immuno-  fluorescence  (Macrophages) | **Primary antibodies** | Rabbit monoclonal anti- Integrin α4 antibody | Cell Signaling Technology | #8440 |
|  |  | Rabbit polyclonal anti- Integrin β1 antibody | Proteintech | 12594-1-AP |
|  |  | Rabbit polyclonal anti- CCR2 antibody | Zenbio | 383757 |
|  |  | Mouse monoclonal anti-Integrin αvβ3 antibody | Santa Cruz | sc-7312 |
|  | **Secondary antibodies** | Goat Anti-Rabbit IgG H&L (Alexa Fluor® 488) | Abcam | ab150077 |
|  |  | Goat Anti-Mouse IgG H&L (Alexa Fluor® 594) | Abcam | ab150116 |
| Western blotting  (HL-60) | **Primary antibodies** | Rabbit polyclonal anti- CXCR1 antibody | Abcam | ab124344 |
|  |  | Rabbit polyclonal anti- CXCR2 antibody | Abcam | ab65968 |
|  |  | Rabbit polyclonal anti-LFA-1 antibody | Abcam | ab185723 |
|  |  | Rabbit monoclonal anti- Sodium Potassium ATPase antibody | Abcam | ab76020 |
|  | **Secondary antibodies** | HRP-conjugated Affinipure Goat Anti-Rabbit IgG(H+L) | Proteintech | SA00001-2 |
| Western blotting  (Macrophages) | **Primary antibodies** | Rabbit polyclonal  anti-CCR2 antibody | Zenbio | 383757 |
|  |  | Rabbit polyclonal  anti-Integrin α4 antibody | Proteintech | 19676-1-AP |
|  |  | Rabbit polyclonal  anti-Integrin β1 antibody | Proteintech | 12594-1-AP |
|  |  | Rabbit polyclonal anti-Integrin αvβ3 antibody | Bioss | BS-1310R |
|  |  | Rabbit monoclonal anti- Sodium Potassium ATPase antibody | Abcam | ab76020 |
|  | **Secondary antibodies** | HRP-conjugated Affinipure Goat Anti-Rabbit IgG(H+L) | Proteintech | SA00001-2 |
| Immuno-  fluorescence  (Tissue sections) | **Primary antibodies** | Rabbit monoclonal anti-SQSTM1/p62 antibody | Abcam | ab109012 |
|  |  | Rabbit monoclonal anti-LC3A/B antibody | Cell Signaling Technology | #12741 |
|  |  | Rabbit monoclonal anti-NLRP3 antibody | Abcam | ab270449 |
|  |  | Rabbit polyclonal anti-GSDMD antibody | Proteintech | 20770-1-AP |
|  |  | Rabbit monoclonal anti-iNOS antibody | Abcam | ab178945 |
|  |  | Rabbit polyclonal anti- Arginase-1 antibody | Proteintech | 16001-1-AP |
|  |  | Mouse monoclonal anti-NeuN antibody | Abcam | ab104224 |
|  |  | Mouse monoclonal anti-Iba1 antibody | Abcam | ab283319 |
|  |  | Mouse monoclonal anti- Chondroitin Sulfate antibody | Abcam | ab11570 |
|  |  | Rabbit monoclonal anti-GFAP antibody | Abcam | ab68428 |
|  |  | Mouse monoclonal anti-GFAP antibody | Proteintech | 60190-1-Ig |
|  |  | Rabbit monoclonal anti-CD31 antibody | Abcam | ab222783 |
|  |  | Rabbit polyclonal anti-beta III Tubulin antibody | Abcam | ab18207 |
|  |  | Rabbit polyclonal anti-MAP2 Tubulin antibody | Abcam | ab32454 |
|  | **Secondary antibodies** | Goat Anti-Rabbit IgG H&L (Alexa Fluor® 488) | Abcam | ab150077 |
|  |  | Goat Anti-Mouse IgG H&L (Alexa Fluor® 488) | Abcam | ab150113 |
|  |  | Goat Anti-Rabbit IgG H&L (Alexa Fluor® 594) | Abcam | ab150080 |
|  |  | Goat Anti-Mouse IgG H&L (Alexa Fluor® 594) | Abcam | ab150116 |
| Western blotting  (Tissue sections) | **Primary antibodies** | Rabbit monoclonal anti-LC3A/B antibody | Cell Signaling Technology | #12741 |
|  |  | Rabbit monoclonal anti-Beclin 1 antibody | Abcam | ab207612 |
|  |  | Rabbit monoclonal anti-SQSTM1/p62 antibody | Abcam | ab109012 |
|  |  | Rabbit monoclonal anti-Caspase-1 antibody | Cell Signaling Technology | #83383 |
|  |  | Rabbit monoclonal anti-GSDMD antibody | Abcam | ab219800 |
|  |  | Rabbit monoclonal anti-NLRP3 antibody | Abcam | ab270449 |
|  |  | Rabbit polyclonal anti-NLRP1 antibody | Affinity | #DF13187 |
|  |  | Rabbit monoclonal anti-ASC/TMS1 antibody | Cell Signaling Technology | #67824 |
|  |  | Rabbit monoclonal anti-IL-1 beta antibody | Abcam | ab234437 |
|  |  | Rabbit monoclonal anti-IL-18 antibody | Abcam | ab207323 |
|  |  | Rabbit monoclonal anti-iNOS antibody | Abcam | ab178945 |
|  |  | Rabbit polyclonal anti- Arginase-1 antibody | Proteintech | 16001-1-AP |
|  |  | Mouse monoclonal anti-GAPDH antibody | Abcam | ab8245 |
|  | **Secondary antibodies** | HRP-conjugated Affinipure Goat Anti-Rabbit IgG(H+L) | Proteintech | SA00001-2 |
|  |  | HRP-conjugated Affinipure Goat Anti-Mouse IgG(H+L) | Proteintech | SA00001-1 |
